# Asymmetric Synthesis of Methoxylated Ether Lipids: Total Synthesis of Polyunsaturated C18:3 Omega-3 and Omega-6 MEL Triene Derivatives

**DOI:** 10.3390/molecules29010223

**Published:** 2023-12-31

**Authors:** Svanur Sigurjónsson, Gudmundur G. Haraldsson

**Affiliations:** Chemistry Department, Science Institute, University of Iceland, Dunhaga 3, 107 Reykjavik, Iceland; svs29@hi.is

**Keywords:** asymmetric synthesis, methoxylated ether lipid (MEL), semi-hydrogenation, shark liver oil

## Abstract

The asymmetric synthesis of polyunsaturated triene C18:3 n-3 and C18:3 n-6 methoxylated ether lipids (MEL) of the 1-*O*-alkyl-*sn*-glycerol type is described as possible structural candidates for a triene C18:3 MEL of an unknown identity found in a mixture of shark and dogfish liver oil. Their C18:3 hydrocarbon chains constitute an all-*cis* methylene skipped n-3 or n-6 triene framework, along with a methoxyl group at the 2′-position and *R*-configuration of the resulting stereogenic center. The methoxylated polyenes are attached by an ether linkage to the *pro-S* hydroxymethyl group of the glycerol backbone. The syntheses were based on the polyacetylene approach that involves a semi-hydrogenation of the resulting triynes. Both syntheses were started from our previously described enantio- and diastereomerically pure isopropylidene-protected glyceryl glycidyl ether, a double-C3 building block that was designed as a head group synthon for the synthesis of various types of MELs.

## 1. Introduction

Non-polar ether lipids (ELs), triacylglycerols, and squalene are the three major lipid constituents present in the liver oil of shark and cartilaginous fish [1,2]. The ELs consist of 1-*O*-alkyl-*sn*-glycerols to which two fatty acyl groups are linked as carboxylate esters and, accordingly, they are often named as diacylglyceryl ethers (DAGEs). Numerous benefits on human and animal health are attributed to these ELs [3,4,5], but more so when their 1-*O*-hydrocarbon ether moiety is rendered with monounsaturation [6]. 

The methoxylated ether lipids (MELs) are quite a noteworthy subclass of the 1-*O*-alkyl-*sn*-glycerols. Their 1-*O*-hydrocarbon ether entity is substituted with a methoxyl group at the 2′-position and the resulting chiral center possesses the *R*-configuration [2,7,8,9]. The MELs are also present in the liver oil of elasmobranch fish and shark, where they commonly represent 2–4% of the 1-*O*-alkyl-*sn*-glycerols in the oil. The MELs occur widely in nature in low concentrations including mammals and humans and they have been observed to offer numerous biological activities of importance such as various antimicrobial and anticarcinogenic effects, and to stimulate the immune system [2,10,11].

**Figure 1:**
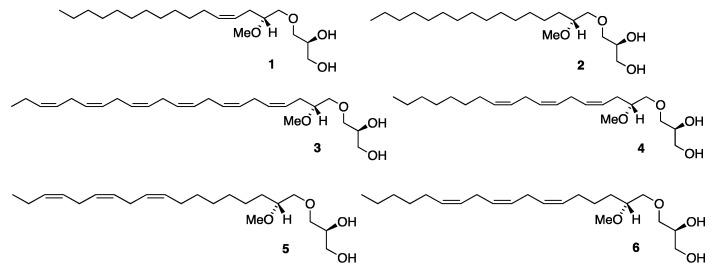
The structure of MELs **1**–**6**.

The two most prevalent components of the MEL fraction are the monounsaturated C16:1 MEL **1** with a *cis* double bond located at the 4’-position (n-12) of the olefin chain, and a saturated C16:0 MEL **2** (see Figure 1). Their C18:1 and C18:0 MEL homologues are also relatively common, and recently, we reported the discovery of a novel C18:1 n-9 MEL regioisomer [12]. Polyunsaturated MELs are also known and that includes an amazing docosahexaenoic acid (DHA)-like C22:6 n-3 MEL **3** (Figure 1) that was discovered in shark liver oil [8].

Our investigation of an MEL sample derived from a mixture of shark and dogfish liver oils revealed the presence of all six of the above-stated MEL derivatives in the natural oil [12]. They were all synthesized and the identity of the unsaturated MELs from the sample was unequivocally confirmed via comparison studies between their MS/MS spectra and those obtained from the synthesized compounds [12]. All syntheses were based on the application of a glyceryl glycidol ether key building block that was recently designed for such MEL asymmetric syntheses and derived from *R*-solketal as a chiral precursor [13,14].

In addition to the six above-described MELs, there was a triene C18:3 MEL of an unknown identity detected in the sample, as was clearly evident from its accurate mass analysis (HRMS (ESI) *m/z*: [M + Li]^+^ theoretical for C_22_H_40_O_4_Li 375.3081; found, 375.3077) [12,15]. We picked out the C18:3 n-8 MEL **4** for synthesis as the most likely structural candidate for the unknown MEL with the aim of having its MS/MS spectrum compared to that of the unknown one. The successful synthesis of the MEL **4** was described in a recent report, but since its MS/MS degradation spectrum failed to align with the spectrum obtained from the natural sample, other alternative structures had to be considered [16]. Other likely candidates were thought to be the C18:3 n-3 MEL **5**, an α-linolenic acid (ALA)-like derivative and the C18:3 n-6 MEL **6**, a γ-linolenic acid (GLA)-like derivative. Their asymmetric syntheses are described in the current report as based on the already established polyacetylene approach [14,16]. The structure of the MELs **1**–**6** is shown in Figure 1.

## 2. Results and Discussion

Hayashi and Takagi in 1982 reported the presence of a C18:3 MEL in cartilaginous fish liver oil without providing further information on its structure [17]. Our initial guess was that the C18:3 MEL most likely had the first double bond of a proposed methylene-interrupted triene framework located in the Δ4′-position, thus making it the C18:3 n-8 MEL **4**. Its synthesis was reported recently [16], but when the MS/MS degradation spectrum of the synthesized MEL **4** did not align with the spectrum obtained from the natural sample, alternative structures had to be considered. Other likely candidates were thought to be the C18:3 n-3 MEL **5**, possessing the n-3 methylene-interrupted triene framework identical to the one present in α-linolenic acid (ALA), or the C18:3 n-6 MEL **6**, with the corresponding n-6 triene framework as found in γ-linolenic acid (GLA). 

The synthesis of MELs **5** and **6** is reported in the current paper. Their convergent syntheses were based on the polyacetylene approach [18,19] already established in the syntheses of the MELs **3** [14] and **4** [16] by iterative copper-promoted coupling reactions of propargyl bromides with terminal alkynes [20,21] and stereoselective semi-hydrogenation [22] of the resulting triyne. As in the previous cases, the enantio- and diastereopure glyceryl glycidol ether (2*R*,2′*S*)-**7** [13] was used as a double-chiral precursor to control the stereochemistry of the C6 head group segment of the molecules. It was thought of interest to have the MELs **4–6** synthesized with possibilities to have them later screened for some interesting bioactivities, regardless of their structures to fit the undisclosed C18:3 MEL derivative.

### 2.1. Synthesis of MEL ***5***

**Scheme 1:**
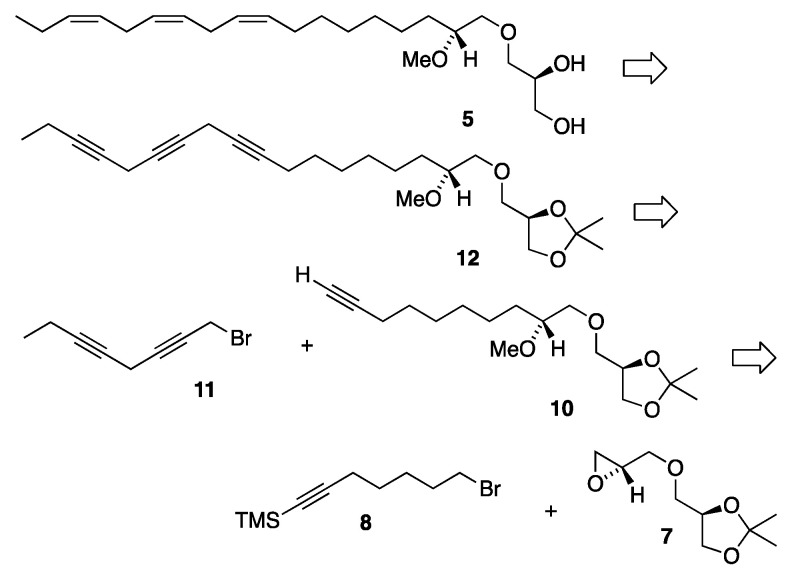
Retrosynthetic analysis for the synthesis of MEL **5**.

The second proposed structural candidate for the unknown C18:3 MEL was the C18:3 n-3 MEL **5** and its synthetic strategy is disclosed in the retrosynthetic analysis displayed in Scheme 1. This is a convergent synthesis involving a Z-selective semi-hydrogenation of the methylene-interrupted omega-3 triyne framework of the isopropylidene protected glyceryl ether **12**. Triyne **12** is disconnected into two subunit fragments, the monyne **10** possessing the glyceryl head part and the diyne **11** possessing the hydrocarbon tail segment. Since the first double bond in the 1-O-hydrocarbon chain was located at the Δ9′-position, we made use of the monoyne **10**, an already known intermediate from our previous synthesis of the 18:1 n-9 MEL [12]. Furthermore, the diyne **11** was also available from the recently reported synthesis of the n-3 polyunsaturated DHA-like MEL **3** [14]. The monoyne **10** is further disconnected into the key head piece **7** and the TMS protected monoyne bromide linker **8** from the previous synthesis of the 18:1 n-9 MEL [12].

**Scheme 2:**
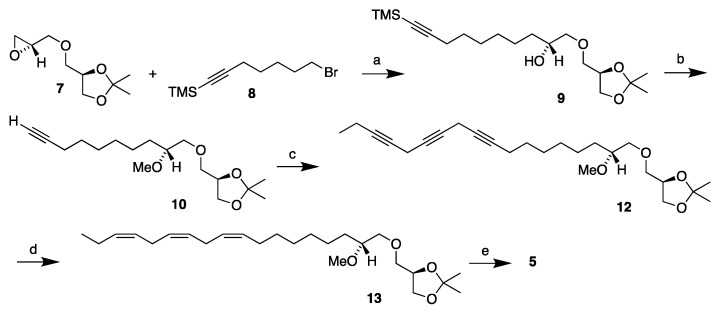
Synthesis of the MEL **5** starting from the head piece **7**. Reagents and conditions: (a) Mg, CuBr, THF, −10 °C (92%); (b) KOH, MeI, DMF, r.t. (79%); (c) 1-bromo-2,5-octadiyne **11**, CuI, NaI, Cs_2_CO_3_, DMF, r.t. (91%); (d) H_2_, Lindlar cat., quinoline, toluene, r.t. (40%); and (e) Amberlyst-15^®^, 96% EtOH, reflux (89%).

Based on the above retrosynthetic analysis, the synthesis of the MEL **5** is depicted in Scheme 2, starting from the head piece **7**. The synthesis of monoyne **10** has already been reported as stated above. It was accomplished in a 73% overall yield from the reaction of the head piece **7** in its reaction with the monoyne linker **8** and the subsequent one-pot TMS deprotection–methylation of the resulting alcohol [12]. The monoyne **10** was coupled to the diyne **11**, accessible from the previously stated synthesis of the polyunsaturated DHA-like MEL **3** [14] that established the desired methylene-interrupted triyne structure. This was accomplished by the copper-promoted coupling that was described in detail in our previous synthesis of the n-3 polyunsaturated DHA-like MEL, with cesium carbonate as a base [14]. This afforded the triyne **12** in a 91% yield. 

The triyne **12** was semi-hydrogenated in the same manner as described for the MEL **4** [16] by use of the Lindlar catalyst with quinoline in toluene at room temperature. The isopropylidene-protected triene **13** was afforded in a 78% yield after flash chromatography and then in a 51% yield after purification by use of argentation chromatography on silver nitrate-impregnated silica gel [14,16,23,24]. This means a 40% yield in all for the semi-hydrogenation step. Finally, like before, the triene **13** was refluxed in 96% ethanol in the presence of acidic Amberlyst-15^®^ ion exchange resin to remove the isopropylidene group [25], affording the MEL **5** in an 89% yield. The MEL **5** was thus obtained in a 24% overall yield as based on the head piece **7** over five steps (17% as based on the *R*-solketal), which is quite comparable to the yield obtained in the MEL **4** synthesis.

The over-hydrogenation of the triyne **12** in the semi-hydrogenation was significantly higher than for the corresponding step in the synthesis of the triene MEL **4** [16]. In that case, the isopropylidene-protected triene was obtained in a 98% yield with 10% contamination of over-hydrogenated byproducts. In the current case, the triene **13** was obtained in a 78% yield with 14% of over-hydrogenated byproducts present. As described before [14], the level of over-hydrogenation was accurately determined using ^1^H NMR analysis by comparing the integration of the olefin protons to that of the protons belonging to the glycerol moiety, with the olefin protons integrating 5.17 out of 6. After purification via argentation chromatography, the over-hydrogenated byproducts had dropped down to <0.5% (5.98/6) in the product.

This is comparable to what was accomplished in the corresponding step in the synthesis of the MEL **4,** where the final product was obtained free of over-hydrogenated byproducts and presumably *trans*-isomers also [16]. We are unable to accurately measure the *trans*-isomer content of the triene **13,** but from arguments based on comparison studies with the n-3 polyunsaturated fatty acids (PUFAs) provided in our previous synthesis of MEL **3** [14], we are confident that it remains very low and in no more than trace amounts in our current synthesis of the purified C18:3 n-8 MEL **5**. This will be further clarified below in relation to the synthesis of the MEL **6**. Again, the power of the argentation chromatography is nicely demonstrated by these results in terms of removing virtually all the over-hydrogenated and *trans*-isomer byproducts.

### 2.2. Synthesis of MEL ***6***

**Scheme 3:**
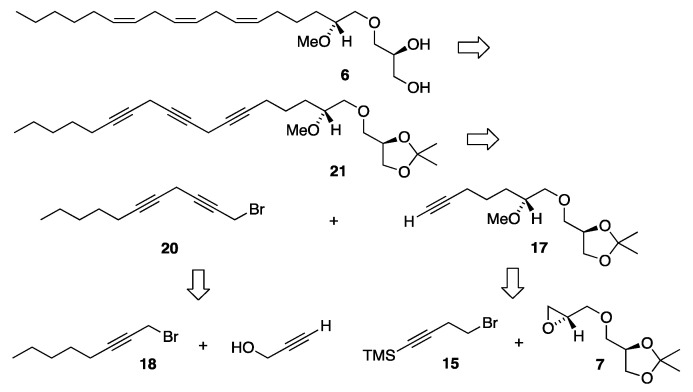
Retrosynthetic analysis for the synthesis of MEL **6**.

As neither of the n-8 and n-3 18:3 MELs **4** and **5** did align with the MS/MS spectra of the natural compound (see below), a third and final 18:3 MEL synthesis of an unusual n-6 variant was performed. The strategy for the C18:3 n-6 MEL **6** synthesis is disclosed in the retrosynthetic analysis shown in Scheme 3. This is also a convergent synthesis involving a Z-selective semi-hydrogenation of the methylene-interrupted omega-6 triyne framework of the isopropylidene-protected glyceryl ether **21**. Triyne **21** is disconnected into two subunit fragments, the monyne **17** possessing the glyceryl head part, and the diyne bromide **20** possessing the hydrocarbon tail segment. The monoyne **17** is further disconnected into the key head piece **7** and the TMS-protected monoyne bromide linker **15**.

**Scheme 4:**
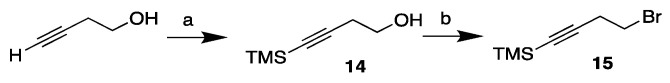
Synthesis of the TMS-protected monoyne bromide linker **15**. Reagents and conditions: (a) n-BuLi (2 eq), TMSCl (2 eq), THF, −78 °C, then 2 M HCl (74%); (b) PPh_3_, CBr_4_, CH_2_Cl_2_, 0 °C (96%).

To accomplish the intended n-6 methylene-skipped triene structure, a shorter linker was required since the first double bond in the 1-O-hydrocarbon chain was located at the Δ6*′*-position. Our synthesis of the monoyne linker **15**, which is a commercially available compound, is depicted in Scheme 4.

But-3-yn-1-ol was allowed to react with two equivalents of TMS-chloride and n-butyllithium in dry THF to afford the monoyne homopropargyl alcohol **14** in a 74% yield after the addition of 2.0 M hydrochloric acid and vigorous stirring. The alcohol was then brominated in an Appel-type reaction [26], affording the monoyne linker **15** in a 96% yield.

**Scheme 5:**
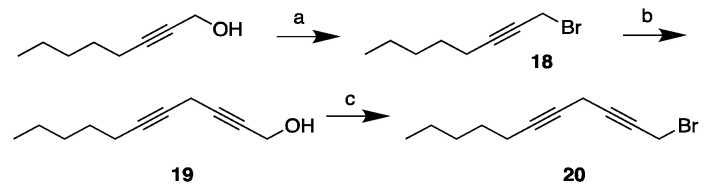
Synthesis of the diyne bromide tail fragment **20**. Reagents and conditions: (a) PPh_3_, CBr_4_, CH_2_Cl_2_, 0 °C (100%); (b) Propargyl alcohol, CuI, NaI, Cs_2_CO_3_, DMF, r.t. (74%); and (c) PPh_3_, CBr_4_, CH_2_Cl_2_, 0 °C (89%).

The synthesis of the n-6 diyne tail piece **20** is depicted in Scheme 5. Its synthesis was accomplished by the Appel-type bromination of oct-2-yn-1-ol, which afforded the monoyne **18** in a quantitative yield after flash chromatography. Copper coupling of the resulting monoyne **18** and propargyl alcohol gave the diyne alcohol **19** in a 74% yield. The diyne **20** was then afforded in a second Appel bromination reaction in an 89% yield after flash chromatography. This compound was prepared earlier in relation to the synthesis of arachidonic acid by a different approach [27].

**Scheme 6:**
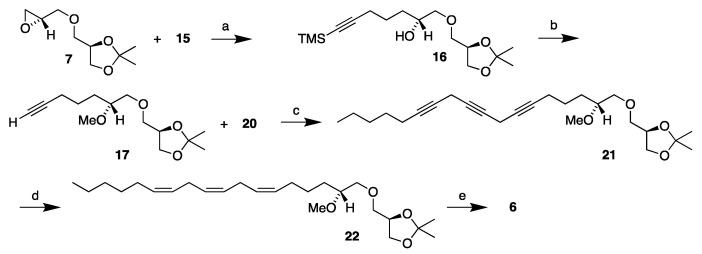
Synthesis of the MEL **6** starting from the head piece **7**. Reagents and conditions: Reagents and conditions: (a) Mg, CuBr, THF, -10 °C (76%); (b) KOH, MeI, DMF, r.t. (83%); (c) CuI, NaI, Cs_2_CO_3_, DMF, r.t. (94%); (d) H_2_, Lindlar cat., quinoline, toluene, r.t. (33%); and (e) Amberlyst-15^®^, 96% EtOH, reflux (100%).

The overall synthetic route to the n-6 MEL **6** is demonstrated in Scheme 6. Like the synthesis of the MEL **5**, the linker **15** was reacted with magnesium metal to obtain the corresponding Grignard reagent, which in turn was reacted with the head piece **7** in the presence of cuprous bromide to obtain the monoyne **16**. Initially, this reaction gave poor yields. This was because some amount of bromoform eluted with the linker **15** off the silica column. To resolve that, some further flash chromatography was applied until no bromoform residue was left and the reaction then proceeded smoothly, affording the monoyne **16** in a 76% yield.

The monoyne **16** was then, like in the cases of the MEL **3** synthesis [14] and monoyne **10** above, deprotected and methylated in the same operation using methyl iodide and potassium hydroxide. And, as before, the substrate would only methylate in part, but with the addition of molecular sieves, the monoyne **17** was afforded in an 83% yield. The monoyne **17** and diyne **20** were then allowed to undergo copper coupling with cesium carbonate as a base, to furnish the triyne **21** in a 94% yield. 

The high yield in the coupling reaction involving a monoyne head part and a diene tail part is noteworthy and warrants a comment. In previous cases involving a monoyne head part with the triple bond located at the Δ4′-position in a close vicinity to the isopropylidene-protected glyceryl head part, far lower yields were obtained, as was evident in the syntheses of both the hexaene MEL **3** [14] and triene MEL **4** [16], where a diyne head part was required for high yields. In the reactions of monoyne head groups **17** and **10** with the corresponding diyne tail parts, this was not a problem, and the reactions took place in excellent yields.

The key semi-hydrogenation step was conducted in the same manner as for the previous 18:3 MELs **4** and **5,** and the triene **22** was accomplished in a 78% yield after flash chromatography. Then, argentation chromatography gave a 43% yield, resulting in a 33% overall yield from the semi-hydrogenation. The semi-hydrogenation’s overall yields were lower for this product than the corresponding trienes of the other 18:3 MEL syntheses. This may be explained by poorer condition of the labile triyne **21** when it was submitted to the semi-hydrogenation, but the reaction was not optimized further. The final isopropylidene deprotection by aid of the acidic Amberlyst-15^®^ ion exchange resin in refluxing ethanol afforded the MEL **6** in a quantitative yield. Thus, the MEL **6** was obtained in a 20% yield from the key head piece **7** over five steps (14% total yield as based on the *R*-solketal).

The over-hydrogenation content of the crude product from the semi-hydrogenation obtained from the flash chromatography was 10%, significantly lower than that obtained for MEL **5** above, but comparable to that obtained in the MEL **4** synthesis. After the argentation chromatography purification, the over-hydrogenation contamination had dropped to 2% (5.86/6), which is significantly higher than that obtained for MELs **4** and **5**. The yield of the purified triene **22** was significantly lower than that obtained for the corresponding **13**, despite the lower purity. 

This is clearly less favorable than what was accomplished in the corresponding step in the synthesis of the MEL **4**, where the final product was obtained free of over-hydrogenated and *trans*-isomer byproducts [15], as well as MEL **5** also. The over-hydrogenation level of 2% indicates that there may still be some *trans*-isomers present (0.5–1%) in the purified triene **22** and therefore in the MEL **6** final product. This is estimated as based on our previous results obtained from the comparison of various long-chain n-3 PUFAs (C18:4, C20:5 and C22:6) prepared under identical semi-hydrogenation conditions as in the MEL syntheses and accurately determined via GLC analysis. This was described and used to estimate the *trans*-isomer byproducts in the synthesis of the hexaene MEL **3** [14] as well as to evaluate the purity of the triene MEL **4** [16].

### 2.3. HPLC-MS/MS Comparison of C18:3 MELs

Surprisingly, none of the above C18:3 MEL structures seemed to correspond to the one found in the natural shark and dogfish liver oil sample, since none of their MS/MS spectra matched. Figure S1 of the Supplementary Material shows the [M+Li]^+^ MS/MS fragmentation of the synthesized MELs **4**, **5** and **6** as compared to the corresponding unknown C18:3 MEL obtained from the shark and dogfish liver oil mixture. Therefore, based on this comparison, it is evident that the true structure of the polyunsaturated C18:3 MEL remains unknown.

### 2.4. Trends in Specific Optical Rotation Values among Unsaturated MELs

The specific optical rotation obtained for MELs **5** and **6** warrants a special comment. It is noteworthy that all the unsaturated MEL derivatives obtained from our syntheses so far, except one, displayed negative specific rotation values in ethanol. This includes C16:1 n-12 MEL **1**, C18:1 n-14 MEL, C22:6 n-3 MEL **3,** and C18:3 n-8 MEL **4,** where there commonly is a carbon–carbon double bond located in the Δ4′ position of the 1-*O*-hydrocarbon ether entity. The C18:1 n-9 MEL, on the other hand, displayed specific optical rotation opposite in direction also measured in ethanol. In that case, the double bond is in the Δ9′ position of the hydrocarbon chain. Similarly, the C18:3 n-3 MEL **5** and the C18:3 n-6 MEL **6** displayed positive specific optical rotation values. They possess the double bond closest to the chiral center located in the Δ9′ and Δ6′ positions of their hydrocarbon moiety, respectively. Table 1 reveals the specific optical rotation values for all our unsaturated MEL derivatives that have been synthesized.

## 3. Materials and Methods

### 3.1. General Information

^1^H and ^13^C nuclear magnetic resonance spectra were recorded on a Bruker Avance 400 spectrometer in deuterated chloroform as a solvent at 400.12 and 100.61 MHz, respectively. Chemical shifts (δ) are reported in parts per million (ppm) and the coupling constants (J) in Hertz (Hz). The following abbreviations are used to describe the multiplicity: s, singlet; d, doublet; t, triplet; q, quartet; quin, quintet; dd, doublet of doublets; and m, multiplet. Structural assignments were made with additional information from COSY and HSQC experiments. Infrared spectra were conducted on a Thermo Nicolet FT-IR iS10 Spectrophotometer on a KBr pellet (crystalline material) or as a neat liquid (oils). Melting points were determined on a Büchi 520 melting point apparatus and are uncorrected. The high-resolution mass spectra (HRMS) were acquired on a Bruker micrOTOF-Q mass spectrometer. All data analysis was performed on Bruker software. Optical activity measurements were performed on an Autopol^R^ V Automatic Polarimeter from Rudolph Research Analytical using a 40T-2.5-100-0.7 Temp Trol™ polarimetric cell with a 2.5 mm inside diameter, a 100 mm optical path length, and a 0.7 mL volume, with c referring to g sample/100 mL. The reactions that required heating were conducted in an aluminum block for a round-bottom flask installed on a magnetic stirrer hotplate equipped with a contact thermometer.

All chemicals and solvents were used without further purification unless otherwise stated. Quinoline (98%), propargyl alcohol (99%), but-3-yn-1-ol (97%), oct-2-yn-1-ol (97%), cuprous iodide (>99.5%), cuprous bromide (98%), sodium iodide (>99%), cesium carbonate (99.9%), tetrabromomethane (99%), triphenylphosphine (99%), trimethylsilyl chloride (≥99%), *n*-butyl lithium in cyclohexane (2.0 M), and palladium on calcium carbonate, 5% wt, poisoned with lead (Lindlar catalyst), were from Sigma-Aldrich (Steinheim, Germany). Molecular sieves 4A were obtained from Acros Organics (New Jersey, USA). Amberlyst-15^®^ ion exchange resin was obtained from Fluka (Buchs, Switzerland). Methyl iodide and potassium hydroxide were purchased from Merck (Darmstad, Germany). Dichloromethane and ethyl acetate were obtained as HPLC grade, as well as toluene, acetone (≥99.8%), and N,N-dimethylformamide (anhydrous, 99,8%) from Sigma-Aldrich (Steinheim, Germany), and petroleum ether, in a boiling range of 40–60 °C, tetrahydrofuran, diethyl ether, methanol, and ethanol (99.8%) were obtained from Honeywell (Seelze, Germany). Dichloromethane was dried over CaH_2_ under a dry nitrogen atmosphere. Tetrahydrofuran HPLC grade was obtained from Sigma-Aldrich (Steinheim) and dried over Na wire in the presence of benzophenone under a dry nitrogen atmosphere. Column chromatography was performed on Silica gel 60 (Silicycle, Quebec, Canada). Reactions were monitored by TLC on Silica gel 60 F254 (Silicycle, Quebec, Canada), with detection by quenching of fluorescence and/or with phosphomolybdic acid in methanol. Argentation column chromatography was performed with 10% Silver nitrate-impregnated silica gel (R23530B) (Silicycle, Quebec, Canada).

### 3.2. Synthesis of MEL ***5***

#### 3.2.1. Synthesis of (2′R)-1-O-(2′-Methoxyocta-9′,12′,15′-triyn-1-yl)-2,3-O-isopropylidene-sn-glycerol, **12**

To a stirred suspension of cuprous iodide (0.360 g, 1.89 mmol), sodium iodide (0.703 g, 4.73 mmol), and cesium carbonate (1.230 g, 3.78 mmol) in dry DMF (5 mL) under nitrogen atmosphere, 1-bromoocta-2,5-diyne **11** [14] (0.350 g, 1.89 mmol) was added in DMF (2 mL), followed by the methoxylated monoyne **10** [12] (0.282 g, 0.945 mmol) in DMF (2 mL). The resulting mixture was stirred vigorously for 41 h at room temperature. Then, the reaction mixture was quenched with a saturated ammonium chloride solution and stirred for 10 min. The mixture was then extracted three times with diethyl ether and the combined ether extracts washed with brine, dried over MgSO_4_, filtered, then concentrated in vacuo. The crude concentrate was purified via flash column chromatography using ethyl acetate/petroleum ether (1:3) as eluent affording to the triyne product **12** as a yellow oil (0.344 g, 91% yield). [α]^D^_20_ = −3.80 (c 2.0, ethanol). IR (NaCl, ν_max_/cm^−1^): 2981, 2935, 2860, 2213. ^1^H NMR (400 MHz, CDCl_3_) δ_H_: 4.29–4.23 (m, 1H, CH *sn*-2), 4.05 (dd, *J* = 8.3, 6.4 Hz, 1H, CH_2_ *sn*-3), 3.75 (dd, *J* = 8.3, 6.3 Hz, 1H, CH_2_ *sn*-3), 3.56 (dd, *J* = 10.0, 5.4 Hz, 1H, CH_2_ *sn*-1), 3.52 (dd, *J* = 10.3, 5.8 Hz, 1H, CH_2_-1′), 3.49 (dd, *J* = 10.0, 5.7 Hz, 1H, CH_2_ *sn*-1), 3.47 (dd, *J* = 10.3, 4.2 Hz, 1H, CH_2_-1′), 3.40 (s, 3H, OCH**_3_**), 3.35–3.28 (m, 1H, CH-2′), 3.15–3.12 (m, 4H, ≡CC*H***_2_**C≡), 2.20–2.12 (m, 4H, ≡CC*H***_2_**CH_2_/CH_3_), 1.53–1.25 (m, 10H, C*H***_2_**), 1.42 (s, 3H, C(CH**_3_**)_2_), 1.35 (s, 3H, C(CH**_3_**)_2_), 1.12 (t, *J* = 7.5 Hz, 3H, CH_2_C*H*_3_) ppm. ^13^C{H} NMR (101 MHz, CDCl_3_) δ_C_: 109.5, 82.3, 80.9, 80.3, 75.01, 74.96, 74.8, 73.94, 73.91, 73.3, 67.0, 57.8, 31.5, 29.4, 29.0, 28.8, 26.9, 25.6, 25.4, 18.8, 14.0, 12.5, 10.0, 9.9 ppm. HRMS (ESI) *m*/*z*: [M + Na]^+^ calcd for C_25_H_38_O_4_Na 425.2668; found, 425.2674.

#### 3.2.2. Synthesis of (2′R)-1-O-[(9′Z,12′Z,15′Z)-2′-Methoxyocta-9′,12′,15′-trien-1-yl]-2,3-O-isopropylidene-sn-glycerol, **13**

The Lindlar catalyst (0.500g) was placed into a dry two-necked round-bottom flask equipped with a magnetic stirrer under nitrogen at room temperature and the flask was sealed with a septum. Toluene (20 mL) was added to the flask with a syringe. A balloon filled with hydrogen gas was then mounted on a syringe and stuck through the septum. The mixture was then stirred while the hydrogen gas was blown through the flask to replace the nitrogen atmosphere with hydrogen. Then quinoline (0.055 g, 0.438 mmol) and triyne **12** (0.353 g, 0.877 mmol), dissolved in toluene (2 mL), were added with a syringe and the reaction was stirred vigorously while being monitored with TLC. When the reaction came to completion according to the TLC (75 min), the flask was promptly opened and the reaction mixture was filtered through a celite layer on a fritted disk using dichloromethane as the eluent. After removing the solvent in vacuo, the crude concentrate was purified via flash column chromatography using ethyl acetate/petroleum ether (1:4) as the eluent, affording the crude product **13** as a light-yellow liquid (0.280 g, 78% yield). Part of the product (0.043 g) was then applied to a 10% silver nitrate-impregnated silica gel column using acetone/petroleum ether (1:4) as the eluent, resulting in the purified product **12** as a faintly yellow liquid (0.022 g, 51% yield; overall in 40% yield). [α]^D^_20_ = −4.25 (c 1.6, ethanol). IR (NaCl, ν_max_/cm^−1^): 3011, 2932, 2857, 846. ^1^H NMR (400 MHz, CDCl_3_) δ_H_: 5.43–5.28 (m, 6H, =CH), 4.29–4.23 (m, 1H, CH *sn*-2), 4.05 (dd, *J* = 8.3, 6.4 Hz, 1H, CH_2_ *sn*-3), 3.76 (dd, *J* = 8.3, 6.3 Hz, 1H, CH_2_ *sn*-3), 3.56 (dd, *J* = 10.0, 5.4 Hz, 1H, CH_2_ *sn*-1), 3.52 (dd, *J* = 10.3, 5.8 Hz, 1H, CH_2_-1′), 3.49 (dd, *J* = 10.0, 5.8 Hz, 1H, CH_2_ *sn*-1), 3.48 (dd, *J* = 10.3, 4.4 Hz, 1H, CH_2_-1′), 3.40 (s, 3H, OCH_3_), 3.35–3.28 (m, 1H, CH-2′), 2.81 (t, *J* = 6.0 Hz, 4H, =CHC*H***_2_**CH=), 2.11–2.03 (m, 4H, =CHC*H***_2_**CH_2_/=CHC*H***_2_**CH_3_), 1.52–1.45 (m, 2H, CH_2_-3′), 1.42 (s, 3H, C(CH**_3_**)_2_), 1.36 (s, 3H, C(CH**_3_**)_2_), 1.40–1.25 (m, 8H, CH_2_), 0.97 (t, *J* = 7.5 Hz, 3H, CH_2_C*H***_3_**) ppm. ^13^C{H} NMR (101 MHz, CDCl_3_) δ_C_: 132.1, 130.4, 128.43, 128.41, 127.9, 127.3, 109.5, 80.3, 74.8, 74.0, 72.5, 67.0, 57.7, 31.6, 29.9, 29.8, 29.4, 27.4, 26.9, 25.8, 25.7, 25.6, 25.5, 20.7, 14.4 ppm. HRMS (ESI) *m*/*z*: [M + Na]^+^ calcd for C_25_H_44_O_4_Na 431.3137; found, 431.3132.

#### 3.2.3. Synthesis of (2′R)-1-O-[(9′Z,12′Z,15′Z)-2′-Methoxyocta-9′,12′,15′-trien-1-yl]-sn-glycerol, **5**

Triene **13** (0.020 g, 0.049 mmol) in ethanol (2.7 mL) was loaded into a two-necked round-bottom flask equipped with a magnetic stirrer and a reflux condenser under nitrogen. To that solution, wet Amberlyst-15^®^ (0.007 g) was added and the reaction was heated to reflux. After refluxing for 3 h, the mixture was allowed to cool, the Amberlyst-15^®^ was filtered off, and the solvent was removed in vacuo. The crude concentrate was then purified via flash column chromatography, eluting first with ethyl acetate/petroleum ether (1:2) and then with pure ethyl acetate to afford the final product MEL **5** as a yellow oil (0.016 g, 89%). [α]^D^_20_ = +4.27 (c 1.1, ethanol). IR (NaCl, ν_max_/cm^−1^): 3403, 3010, 2926, 2855, 1093, 928. ^1^H NMR (400 MHz, CDCl_3_) δ_H_: 5.43–5.28 (m, 6H, =CH), 3.89-3.84 (m, 1H, CH *sn*-2), 3.71 (dd, *J* = 11.4, 3.9 Hz, 1H, CH_2_ *sn*-3), 3.64 (dd, *J* = 11.4, 5.2 Hz, 1H, CH_2_ *sn*-3), 3.61 (dd, *J* = 10.0, 4.0 Hz, 1H, CH_2_ *sn*-1), 3.56 (dd, *J* = 10.5, 3.5 Hz, 1H, CH_2_-1′), 3.54 (dd, *J* = 10.0, 6.4 Hz, 1H, CH_2_ *sn*-1), 3.48 (dd, *J* = 10.5, 6.0 Hz, 1H, CH_2_-1′), 3.40 (s, 3H, OCH**_3_**), 3.35–3.29 (m, 1H, CH-2′), 2.98 (br s, 1H, OH), 2.81 (t, *J* = 6.1 Hz, 4H, =CHC*H***_2_**CH=), 2.32 (br s, 1H, OH), 2.11–2.03 (m, 4H, =CHC*H***_2_**CH_2_/=CHC*H***_2_**CH_3_), 1.54–1.42 (m, 2H, CH_2_-3′), 1.41–1.22 (m, 8H, CH_2_), 0.97 (t, *J* = 7.5 Hz, 3H, CH_2_C*H***_3_**) ppm. ^13^C{H} NMR (101 MHz, CDCl_3_) δ_C_: 132.1, 130.4, 128.44, 128.39, 127.9, 127.3, 80.5, 73.8, 73.5, 70.7, 64.2, 57.5, 31.1, 29.8, 29.7, 29.4, 27.4, 25.8, 25.7, 25.5, 20.7, 14.4 ppm. HRMS (ESI) *m*/*z*: [M + Na]^+^ calcd for C_22_H_40_O_4_Na 391.2824; found, 391.2819.

### 3.3. Synthesis of MEL ***6***

#### 3.3.1. Synthesis of 4-(Trimethylsilyl)but-3-yn-1-ol, **14**

Into a dried round-bottom flask under nitrogen atmosphere containing but-3-yn-1-ol (0.385 g, 5.50 mmol) in tetrahydrofuran (18 mL) at −78 °C, *n*-butyllithium in cyclohexane (6.05 mL, 2 M, 12.10 mmol) was added with a syringe. After 60 min at −78 °C, trimethylsilyl chloride (1.315 g, 12.10 mmol) was added dropwise from a syringe. Five minutes after that, the reaction was allowed to reach room temperature. After 6 h, 2 M HCl (12 mL) was added and the reaction stirred vigorously for 10 min, then a saturated solution of sodium bicarbonate was added and the solution was extracted three times with diethyl ether, dried over MgSO_4_, filtered, and then the solvent evaporated under reduced pressure. The extract was then applied to the silica gel chromatography using ethyl acetate/petroleum ether (1:3) as the eluent, affording the product **14** as a colorless liquid (0.579 g, 74% yield). IR (NaCl, ν_max_/cm^−1^): 3347, 2960, 2900, 2177. ^1^H NMR (400 MHz, CDCl_3_) δ_H_: 3.71 (q, *J* = 6.3 Hz, 2H, HOC*H***_2_**), 2.51 (t, *J* = 6.2 Hz, 2H, ≡CCH**_2_**), 1.75 (t, *J* = 6.4 Hz, 1H, OH), 0.16 (s, 9H, TMS) ppm. ^13^C{H} NMR (101 MHz, CDCl_3_) δ_C_: 103.5, 87.2, 61.0, 24.4, 0.20 (3) ppm. HRMS (ESI) *m*/*z*: [M + Na]^+^ calcd for C_7_H_14_OSiNa 165.0712; found, 165.0706.

#### 3.3.2. Synthesis of 4-Bromo-1-(trimethylsilyl)but-1-yne, **15**

Into a dry round-bottom flask equipped with a magnetic stirrer, TMS-protected but-3-yn-1-ol **14** (0.202 g, 1.60 mmol) dissolved in dichloromethane (20 mL) was placed. The flask was then cooled to 0–4 °C (ice/water), carbon tetrabromide (1.427 g, 4.30 mmol) was added, and the resulting mixture was stirred for 10 min. Then triphenylphosphine (0.939 g, 3.58 mmol) was added, the reaction was stirred for further 10 min, and then more triphenylphosphine (0.376 g, 1.42 mmol) was added in small doses every 30 min until the reaction turned yellow. The reaction mixture was then diluted with diethyl ether (50 mL) and the white precipitate was filtered through a short silica layer (2 cm) using diethyl ether as the eluent. The solvent was removed in vacuo, resulting in a colorless oil which was then applied to the silica gel flash chromatography using ethyl acetate/petroleum ether (1:6) as the eluent, affording the product **15** as a colorless oil (0.710 g, 96% yield). IR (NaCl, ν_max_/cm^−1^): 3019, 2960, 2924, 2899, 2854, 2178. ^1^H NMR (400 MHz, CDCl_3_) δ_H_: 3.43 (t, *J* = 7.5 Hz, 2H, BrCH**_2_**), 2.77 (t, *J* = 7.5 Hz, 2H, ≡CCH**_2_**), 0.16 (s, 9H, TMS) ppm. ^13^C{H} NMR (101 MHz, CDCl_3_) δ_C_: 103.3, 87.2, 29.3, 24.5, 0.1 (3) ppm. Too volatile for HRMS measurement.

#### 3.3.3. (2′R)-1-O-[7′-(Trimethylsilyl)-2′-hydroxyhept-6′-yn-1-yl]-2,3-O-isopropylidene-sn-glycerol, **16**

To a dried two-necked round-bottom flask equipped with a reflux condenser, magnesium (0.078 g, 3.20 mmol) was added under nitrogen atmosphere. To the flask, dry THF (8 mL) and TMS-protected monoyne bromide **15** (0.586 g, 2.86 mmol) in THF (2 mL) was then added via syringe. Heat was applied and the reaction was allowed to reflux until most of the magnesium metal had disappeared. The reaction was then cooled to about −10 °C (ice/acetone) and cuprous bromide (0.046 g, 0.32 mmol) and the head piece **7** [13] (0.301 g, 1.60 mmol) in THF (1 mL) were added. The stirred reaction was allowed to warm up overnight and was then finally quenched with a saturated aqueous ammonium chloride solution, extracted three times with diethyl ether, and the combined ether extracts were washed once with a saturated sodium chloride solution and dried over MgSO_4_. After filtering and evaporation of the solvent, the resulting concentrate was applied to the silica gel column chromatography using ethyl acetate/petroleum ether (1:2) as the eluent, affording the TMS-protected monoyne **16** as a colorless liquid (0.385 g, 76% yield). [α]^D^_20_ = +1.7 (c 0.1, ethanol). IR (NaCl, ν_max_/cm^−1^): 3474, 2986, 2956, 2936, 2900, 2872, 2174. ^1^H NMR (400 MHz, CDCl_3_) δ_H_: 4.33–4.22 (m, 1H, CH *sn*-2), 4.05 (dd, *J* = 8.3, 6.5 Hz, 1H, CH_2_ *sn*-3), 3.86–3.77 (m, 1H, CH-2′), 3.74 (dd, *J* = 8.3, 6.4 Hz, 1H, CH_2_ *sn*-3), 3.56 (d, *J* = 5.3 Hz, 2H, CH_2_ *sn*-1), 3.54 (dd, *J* = 9.7, 3.0 Hz, 1H, CH_2_-1′), 3.35 (dd, *J* = 9.7, 7.9 Hz, 1H, CH_2_-1′), 2.42 (d, *J* = 3.4 Hz, 1H, OH), 2.26 (t, *J* = 6.8 Hz, 2H, ≡CCH_2_), 1.77–1.48 (m, 4H, CH_2_-3′ and ≡CCH_2_C*H*_2_), 1.43 (s, 3H, C(CH_3_)_2_), 1.37 (s, 3H, C(CH_3_)_2_), 0.14 (s, 9H, TMS) ppm. ^13^C{H} NMR (101 MHz, CDCl_3_) δ_C_: 109.7, 107.2, 100.1, 85.0, 76.2, 74.9, 72.5, 70.0, 69.9, 66.7, 32.2, 26.9, 25.5, 24.8, 20.0, 0.3 ppm. HRMS (ESI) *m/z*: [M + Na]^+^ calcd for C_16_H_30_O_4_SiNa 337.1811; found, 337.1806.

#### 3.3.4. (2′R)-1-O-(2′-Methoxydec-6′-yn-1-yl)-2,3-O-isopropylidene-sn-glycerol, **17**

Into a flame-dried round-bottom 25 mL flask equipped with a magnetic stirrer, the TMS-protected alkyne alcohol **16** (0.093 g, 0.296 mmol) dissolved in dry DMF (6 mL) was placed and molecular sieves were added (0.2 g). Into the stirred mixture, potassium hydroxide (0.066 g, 1.18 mmol) was added and stirred for 10 min. Subsequently methyl iodide (0.420 g, 2.96 mmol) was added. The reaction was monitored with TLC, and after two hours, more potassium hydroxide (0.015 g, 0.27 mmol) was added, and then also every 40 min until the reaction was nearly completed according to TLC. When the reaction came to a stop according to TLC, the reaction mixture was quenched with a saturated ammonium chloride solution and extracted three times with diethyl ether. The combined extracts were washed with brine, dried over MgSO_4_, filtered, then concentrated in vacuo. The resulting concentrate was purified via flash silica gel column chromatography using ethyl acetate/petroleum ether (1:1) as the eluent, affording the methoxylated monoyne product **16** as a colorless liquid (0.063 g, 83% yield). [α]^D^_20_ = −3.29 (c 0.7, ethanol). IR (NaCl, ν_max_/cm^−1^): 3290, 2986, 2934, 2872, 2828, 2117. ^1^H NMR (400 MHz, CDCl_3_) δ_H_: 4.30–4.22 (m, 1H, CH *sn*-2), 4.05 (dd, *J* = 8.3, 6.4 Hz, 1H, CH_2_ *sn*-3), 3.75 (dd, *J* = 8.3, 6.3 Hz, 1H, CH_2_ *sn*-3), 3.56 (dd, *J* = 10.0, 5.4 Hz, 1H, CH_2_ *sn*-1), 3.54 (dd, *J* = 10.3, 5.6 Hz, 1H, CH_2_-1′), 3.49 (dd, *J* = 10.0, 5.7 Hz, 1H, CH_2_ *sn*-1), 3.48 (dd, *J* = 10.3, 5.7 Hz, 1H, CH_2_-1′), 3.40 (s, 3H, OCH_3_), 3.37–3.31 (m, 1H, CH-2′), 2.25–2.14 (m, 2H, ≡CCH_2_), 1.94 (t, *J* = 2.6 Hz, 1H, ≡CH), 1.71–1.51 (m, 4H, CH_2_-3′ and ≡CCH_2_C*H*_2_), 1.42 (s, 3H, C(CH_3_)_2_), 1.36 (s, 3H, C(CH_3_)_2_) ppm. ^13^C{H} NMR (101 MHz, CDCl_3_) δ_C_: 109.5, 84.4, 79.7, 74.8, 73.8, 72.6, 68.6, 67.0, 57.8, 30.7, 26.9, 25.6, 24.5, 18.7 ppm. HRMS (ESI) *m/z*: [M + Na]^+^ calcd for C_14_H_24_O_4_Na 279.1572; found, 279.1567.

#### 3.3.5. Synthesis of 1-Bromooct-2-yne, **18**

Into a dry two-necked round-bottom flask equipped with a magnetic stirrer, oct-2-yn-1-ol (0.500 g, 3.96 mmol) dissolved in dichloromethane (22 mL) was placed. The flask was then cooled to 0–4 °C (ice/water), carbon tetrabromide (1.58 g, 4.75 mmol) was added, and the resulting mixture was stirred for 10 min. Then triphenylphosphine (1.040 g, 3.97 mmol) was added and the reaction was stirred for a further 10 min. After 15 min and 30 min, more triphenylphosphine (0.208 g, 0.79 mmol) was added, at which point the reaction mixture turned yellow (in all 1.456 g, 5.55 mmol). The reaction mixture was then diluted with diethyl ether (50 mL) and the white precipitate was filtered through a short silica layer (2 cm) using diethyl ether as the eluent. The solvent was removed in vacuo, resulting in a colorless oil which was then applied to the silica gel flash chromatography using ethyl acetate/petroleum ether (1:6) as the eluent, affording the product **18** as a colorless oil (0.745 g, 100% yield). IR (NaCl, ν_max_/cm^−1^): 2957, 2932, 2871, 2860, 2311, 2234. ^1^H NMR (400 MHz, CDCl_3_) δ_H_: 3.93 (t, *J* = 2.4 Hz, 2H, BrCH**_2_**), 2.23 (tt, *J* = 7.2, 2.4 Hz, 2H, ≡CC*H***_2_**CH_2_), 1.58–1.46 (m, 2H, ≡CCH_2_C*H***_2_**), 1.41–1.23 (m, 4H, C*H***_2_**C*H***_2_**CH_3_), 0.90 (t, *J* = 7.0 Hz, 3H, CH**_3_**) ppm. ^13^C{H} NMR (101 MHz, CDCl_3_) δ_C_: 88.5, 75.4, 31.2, 28.2, 22.3, 19.1, 16.0, 14.1 ppm. Too volatile for HRMS measurement.

#### 3.3.6. Synthesis of Undeca-2,5-diyn-1-ol, **19**

To a stirred suspension of cuprous iodide (0.236 g, 1.24 mmol), sodium iodide (0.184 g, 1.24 mmol), and cesium carbonate (0.403 g, 1.24 mmol) in dry DMF (5 mL) under nitrogen atmosphere was added 1-bromooct-2-yne **18** (0.234 g, 1.24 mmol) in DMF (2 mL), followed by propargyl alcohol (0.069 g, 1.24 mmol). The resulting mixture was stirred vigorously for 24 h at room temperature. Then, the reaction mixture was quenched with a saturated ammonium chloride solution and stirred for 10 min. The mixture was then extracted three times with diethyl ether and the combined ether extracts were washed with brine, dried over MgSO_4_, filtered, then concentrated in vacuo. The crude concentrate was purified via flash column chromatography using ethyl acetate/petroleum ether (1:3) as the eluent, affording the diyne alcohol product **19** as a yellow oil (0.150 g, 74% yield). IR (NaCl, ν_max_/cm^−1^): 3358, 2957, 2932, 2871, 2860, 2259. ^1^H NMR (400 MHz, CDCl_3_) δ_H_: 4.26 (t, *J* = 2.2 Hz, 2H, HOC*H***_2_**), 3.19 (quin, *J* = 2.3 Hz, 2H, ≡CCH**_2_**C≡), 2.15 (tt, *J* = 7.2, 2.4 Hz, 2H, ≡CC*H***_2_**CH_2_), 1.55 (br s, 1H, OH) 1.49 (quin, *J* = 7.3 Hz, 2H, ≡CCH_2_C*H***_2_**), 1.40–1.25 (m, 4H, C*H***_2_**C*H***_2_**CH_3_), 0.89 (t, *J* = 7.2 Hz, 3H, CH**_3_**) ppm. ^13^C{H} NMR (101 MHz, CDCl_3_) δ_C_: 81.4, 81.1, 78.5, 73.4, 51.5, 31.2, 28.6, 22.4, 18.8, 14.1, 10.0 ppm. HRMS (ESI) *m*/*z*: [M + Na]^+^ calcd for C_11_H_16_ONa 187.1099; found, 187.1093.

#### 3.3.7. Synthesis of 1-Bromoundeca-2,5-diyne, **20**

Into a dry two-necked round-bottom flask equipped with a magnetic stirrer, undeca-2,5-diyn-1-ol **19** (0.168 g, 1.02 mmol) dissolved in dichloromethane (20 mL) was placed. The flask was then cooled to 0–4 °C (ice/water), carbon tetrabromide (0.407 g, 1.23 mmol) was added, and the resulting mixture was stirred for 10 min. Triphenylphosphine (0.268 g, 1.02 mmol) was added and the reaction was stirred for a further 40 min. Then, more triphenylphosphine (0.080 g, 0.30 mmol) was added, after which the reaction turned yellow within 20 min and showed completion according to TLC. The reaction mixture was then diluted with diethyl ether (50 mL) and the precipitate was filtered through a short silica layer (2 cm) using diethyl ether as the eluent. The solvent was removed in vacuo, resulting in a yellow oil which was applied to the silica gel flash chromatography using ethyl acetate/petroleum ether (1:6) as the eluent, affording the diyne product **20** as a yellow oil (0.207 g, 89% yield). IR (NaCl, ν_max_/cm^−1^): 3003, 2957, 2931, 2870, 2859, 2268, 2234. ^1^H NMR (400 MHz, CDCl_3_) δ_H_: 3.91 (t, *J* = 2.3 Hz, 2H, BrCH**_2_**), 3.21 (quin, *J* = 2.4 Hz, 2H, ≡CCH**_2_**C≡), 2.15 (tt, *J* = 7.2, 2.4 Hz, 2H, ≡CC*H***_2_**CH_2_), 1.49 (quin, *J* = 7.2 Hz, 2H, ≡CCH_2_C*H***_2_**), 1.40–1.27 (m, 4H, C*H***_2_**C*H***_2_**CH_3_), 0.90 (t, *J* = 7.2 Hz, 3H, CH**_3_**) ppm. ^13^C{H} NMR (101 MHz, CDCl_3_) δ_C_: 82.2, 81.5, 75.2, 72.8, 31.1, 28.4, 22.2, 18.7, 14.9, 14.0, 10.1 ppm. HRMS (ESI) *m*/*z*: [M + Na]^+^ calcd for C_11_H_15_BrNa 249.0255; found, 249.0249.

#### 3.3.8. Synthesis of (2′R)-1-O-(2′-Methoxyocta-6′,9′,12′-triyn-1-yl)-2,3-O-isopropylidene-sn-glycerol, **21**

To a stirred suspension of cuprous iodide (0.119 g, 0.62 mmol), sodium iodide (0.232 g, 1.56 mmol) and cesium carbonate (0.407 g, 1.25 mmol) in dry DMF (3 mL) under nitrogen atmosphere was added 1-bromoundeca-2,5-diyne **20** (0.207 g, 0.91 mmol) in DMF (2 mL), followed by the methoxylated monoyne **17** (0.080 g, 0.312 mmol) in DMF (2 mL). The resulting mixture was stirred vigorously for 43 h at room temperature. Then, the reaction mixture was quenched with a saturated ammonium chloride solution and stirred for 10 min. The mixture was then extracted three times with diethyl ether and the combined ether extracts were washed with brine, dried over MgSO_4_, filtered, then concentrated in vacuo. The crude concentrate was purified via flash column chromatography using ethyl acetate/petroleum ether (4:11) as the eluent, affording the triyne product **21** as a yellow oil (0.119 g, 94% yield). [α]^D^_20_ = -3.14 (c 0.7, ethanol). IR (NaCl, ν_max_/cm^−1^): 2984, 2933, 2872, 2211, 1088. ^1^H NMR (400 MHz, CDCl_3_) δ_H_: 4.30–4.22 (m, 1H, CH *sn*-2), 4.05 (dd, *J* = 8.3, 6.4 Hz, 1H, CH_2_ *sn*-3), 3.76 (dd, *J* = 8.3, 6.3 Hz, 1H, CH_2_ *sn*-3), 3.56 (dd, *J* = 10.0, 5.4 Hz, 1H, CH_2_ *sn*-1), 3.53 (dd, *J* = 10.3, 5.8 Hz, 1H, CH_2_-1′), 3.49 (dd, *J* = 10.0, 5.7 Hz, 1H, CH_2_ *sn*-1), 3.48 (dd, *J* = 10.3, 4.3 Hz, 1H, CH_2_-1′), 3.40 (s, 3H, OCH_3_), 3.37–3.30 (m, 1H, CH-2′), 3.15–3.09 (m, 4H, ≡CCH**_2_**C≡), 2.22–2.10 (m, 4H, ≡CC*H***_2_**CH_2_), 1.68–1.44 (m, 6H, C*H***_2_**), 1.42 (s, 3H, C(CH**_3_**)_2_), 1.36 (s, 3H, C(CH**_3_**)_2_), 1.38–1.23 (m, 4H, C*H***_2_**C*H***_2_**CH_3_), 0.89 (t, *J* = 7.1 Hz, 3H, CH_2_C*H*_3_) ppm. ^13^C{H} NMR (101 MHz, CDCl_3_) δ_C_: 109.5, 81.1, 80.5, 79.8, 75.1, 74.9, 74.8, 74.3, 73.84, 73.81, 72.6, 67.0, 57.8, 31.2, 30.8, 28.6, 26.9, 25.6, 24.7, 22.4, 19.0, 18.8, 14.1, 9.9 ppm. HRMS (ESI) *m/z*: [M + Na]^+^ calcd for C_25_H_38_O_4_Na 425.2668; found, 425.2662.

#### 3.3.9. Synthesis of (2′R)-1-O-[(6′Z,9′Z,12′Z)-2′-Methoxyocta-6′,9′,12′-trien-1-yl]-2,3-O-isopropylidene-sn-glycerol, **22**

The Lindlar catalyst (0.117 g) was placed into a dry two-necked round-bottom flask equipped with a magnetic stirrer under nitrogen atmosphere at room temperature and the flask was sealed with a septum. Toluene (5 mL) was added to the flask with a syringe. A balloon filled with hydrogen gas was then mounted on a syringe and stuck through the septum. The mixture was then stirred while the hydrogen gas was blown through the flask to replace the nitrogen atmosphere with hydrogen. Then quinoline (0.018 g, 0.145 mmol) and triyne **21** (0.109 g, 0.290 mmol), dissolved in toluene (2 mL), were added with a syringe and the reaction was stirred vigorously while being monitored with TLC. When the reaction came to completion according to the TLC (40 min), the flask was promptly opened and the reaction mixture was filtered through a celite layer on a fritted disk using dichloromethane as the eluent. After removing the solvent in vacuo, the crude concentrate was purified via flash column chromatography using ethyl acetate/petroleum ether (1:4) as the eluent, affording the crude product **22** as a light-yellow liquid (0.087 g, 78% yield). The crude product was then applied to a 10% silver nitrate-impregnated silica gel column using an ethyl acetate/petroleum ether gradient from 1:7 to 7:9 as the eluent, resulting in the purified triene product **22** as a faintly yellow liquid (0.037 g, 43% yield; overall in a 33% yield). [α]^D^_20_ = −6.88 (c 0.9, ethanol). IR (NaCl, ν_max_/cm^−1^): 3010, 2985, 2930, 2860, 1652, 975. ^1^H NMR (400 MHz, CDCl_3_) δ_H_: 5.45–5.28 (m, 6H, =CH), 4.32–4.21 (m, 1H, CH *sn*-2), 4.05 (dd, *J* = 8.3, 6.4 Hz, 1H, CH_2_ *sn*-3), 3.76 (dd, *J* = 8.3, 6.3 Hz, 1H, CH_2_ *sn*-3), 3.57 (dd, *J* = 10.0, 5.3 Hz, 1H, CH_2_ *sn*-1), 3.55–3.48 (m, 2H, CH_2_-1′ and CH_2_ *sn*-1), 3.48 (dd, *J* = 10.3, 4.4 Hz, 1H, CH_2_-1′), 3.40 (s, 3H, OCH_3_), 3.36–3.29 (m, 1H, CH-2′), 2.80 (t, *J* = 5.8 Hz, 4H, =CHC*H***_2_**CH=), 2.13-2.01 (m, 4H, =CHC*H***_2_**), 1.55–1.43 (m, 4H, CH_2_-3′ and CH_2_-4′), 1.42 (s, 3H, C(CH**_3_**)_2_), 1.36 (s, 3H, C(CH**_3_**)_2_), 1.40–1.24 (m, 6H, CH_2_), 0.89 (t, *J* = 6.8 Hz, 3H, CH_2_C*H***_3_**) ppm. ^13^C{H} NMR (101 MHz, CDCl_3_) δ_C_: 130.5, 129.9, 128.4, 128.3, 128.1, 127.6, 109.5, 80.1, 74.7, 73.8, 72.4, 66.9, 57.7, 31.5, 31.1, 29.4, 27.3, 27.2, 26.8, 25.7 (2), 25.5, 25.4, 22.6, 14.1 ppm. HRMS (ESI) *m*/*z*: [M + Na]^+^ calcd for C_25_H_44_O_4_Na 431.3137; found, 431.3132.

#### 3.3.10. Synthesis of (2′R)-1-O-[(6′Z,9′Z,12′Z)-2′-Methoxyocta-6′,9′,12′-trien-1-yl]-sn-glycerol, **6**

Triene **22** (0.017 g, 0.042 mmol) in ethanol (3 mL) was loaded into a two-necked round-bottom flask equipped with a magnetic stirrer and a reflux condenser under nitrogen. To that solution, wet Amberlyst-15^®^ (0.007 g) was added and the reaction was heated to reflux. After refluxing for 3 h, the mixture was allowed to cool, the Amberlyst-15^®^ was filtered off and the solvent was removed in vacuo. The crude concentrate was then purified via flash column chromatography, eluting first with ethyl acetate/petroleum ether (1:2) and then with pure ethyl acetate to afford the final product MEL **6** as a yellow oil (0.015 g, 100%). [α]^D^_20_ = +3.26 (c 1.5, ethanol). IR (NaCl, ν_max_/cm^−1^): 3404, 3011, 2927, 2858, 1651. ^1^H NMR (400 MHz, CDCl_3_) δ_H_: 5.44–5.28 (m, 6H, =CH), 3.89–3.84 (br m, 1H, CH *sn*-2), 3.73–3.69 (br dd, 1H, CH_2_ *sn*-3), 3.66–3.63 (br dd, 1H, CH_2_ *sn*-3), 3.61 (dd, *J* = 10.0, 3.9 Hz, 1H, CH_2_ *sn*-1), 3.56 (dd, *J* = 10.5, 3.6 Hz, 1H, CH_2_-1′), 3.54 (dd, *J* = 10.0, 6.3 Hz, 1H, CH_2_ *sn*-1), 3.48 (dd, *J* = 10.5, 6.0 Hz, 1H, CH_2_-1′), 3.40 (s, 3H, OCH**_3_**), 3.36–3.30 (m, 1H, CH-2′), 2.95 (br s, 1H, OH), 2.80 (t, *J* = 5.7 Hz, 4H, =CHC*H***_2_**CH=), 2.28 (br s, 1H, OH), 2.14–2.00 (m, 4H, =CHC*H***_2_**CH_2_), 1.58–1.22 (m, 10H, CH_2_), 0.89 (t, *J* = 6.5 Hz, 3H, CH_2_C*H***_3_**) ppm. ^13^C{H} NMR (101 MHz, CDCl_3_) δ_C_: 130.6, 129.8, 128.6, 128.5, 128.2, 127.7, 80.4, 73.8, 73.5, 70.7, 64.2, 57.6, 31.7 30.7, 29.5 27.4, 27.4, 25.8 (2), 25.5, 22.7, 14.2 ppm. HRMS (ESI) *m*/*z*: [M + Na]^+^ calcd for C_22_H_40_O_4_Na 391.2824; found, 391.2819.

## 4. Conclusions

The successful asymmetric synthesis of the trienes C18:3 n-3 MEL **5** and C18:3 n-6 MEL **6** is described. Both syntheses were based on the use of the head group synthon (2*R*,2′*S*)-**7,** which is a double-C3 building block. The syntheses were brought about by the polyacetylene approach, involving a crucial stereoselective semi-hydrogenation of the triynes **12** and **21**. The n-3 C18:3 MEL **5** was obtained virtually free of over-hydrogenation and *trans*-isomer byproducts after flash chromatography and a subsequent argentation chromatography treatment of the triene **13** intermediate obtained from the semi-hydrogenation key step of the synthesis. The n-6 C18:3 MEL **6** was obtained in an acceptable but somewhat lower purity after similar purification treatment of the triene **22**. Neither of the products were observed to match with the unknown C18:3 MEL that was found in a mixture of shark and dogfish liver oil in terms of their MS/MS spectra, so its identity remains undisclosed.

## Figures and Tables

**Table 1:** Comparison of optical activity data for unsaturated MEL derivatives.

MEL Derivative	Location of First Double Bond	[α]^20^_D_	Conditions *
C16:1 n-12 (**1**)	Δ4′	−4.9	c, 1.42, ethanol [13]
C18:1 n-14	Δ4′	−4.2	c, 2.94, ethanol [12]
C18:3 n-8 (**4**)	Δ4′	−4.7	c, 1.7, ethanol [16]
C22:6 n-3 (**3**)	Δ4′	−3.7	c, 0.13, ethanol [14]
C18:1 n-9	Δ9′	+3.7	c, 2.36, ethanol [12]
C18:3 n-3 (**5**)	Δ9′	+4.3	c, 1.1, ethanol *
C18:3 n-6 (**6**)	Δ6′	+3.3	c, 1.5, ethanol *

* Current work.

## Data Availability

The data underlying this study are available in the published article and its online Supplementary Materials.

## Supplementary Materials

The following supporting information can be downloaded at: https://www.mdpi.com/article/10.3390/molecules29010223/s1, Figure S1: Comparison of the MS/MS fragmentation spectra of the unknown C18:3 MEL from the natural dogfish and shark liver oil mixture with MEL **4** from a previous synthesis and MELs **5** and **6** obtained from the current synthesis; Spectral data of all compounds synthesized **5**, **6**, **12**–**14** and **16**–**22** (^1^H, ^13^C, COSY and HSQC NMR); for **15** (^1^H and ^13^C NMR).
