# Supplementary material for: Asymmetric Synthesis of Methoxylated Ether Lipids: Total Synthesis of Polyunsaturated C18:3 Omega-3 and Omega-6 MEL Triene Derivatives
Source: Molecules. 2023 Dec 31;29(1):223. doi: 10.3390/molecules29010223 (PMC10780507; doi:10.3390/molecules29010223)
Supplement: Supplementary file 1 [file molecules-29-00223-s001.zip › molecules-2794204-supplementary.pdf]

Supporting Information

Asymmetric synthesis of methoxylated ether lipids: Total synthesis of polyunsaturated C18:3 omega-3 and omega-6 MEL triene derivatives

Svanur Sigurjónsson and Gudmundur G. Haraldsson*

Science Institute, Chemistry Department, University of Iceland, Dunhaga 3, 107 Reykjavik, Iceland

Table of contents

NMR Spectra *S2 – S34*

Compound **12** *S2*

Compound **13** *S5*

Compound MEL **5** *S8*

Compound **14** *S11*

Compound **15** *S13*

Compound **16** *S14*

Compound **17** *S17*

Compound **18** *S20*

Compound **19** *S22*

Compound **20** *S24*

Compound **21** *S26*

Compound **22** *S29*

Compound MEL **6** *S32*

MS/MS Spectra *S35*

Figure *S1* *S35*

^{1}H NMR (400 MHz, $CDCl_3$) of compound **12**

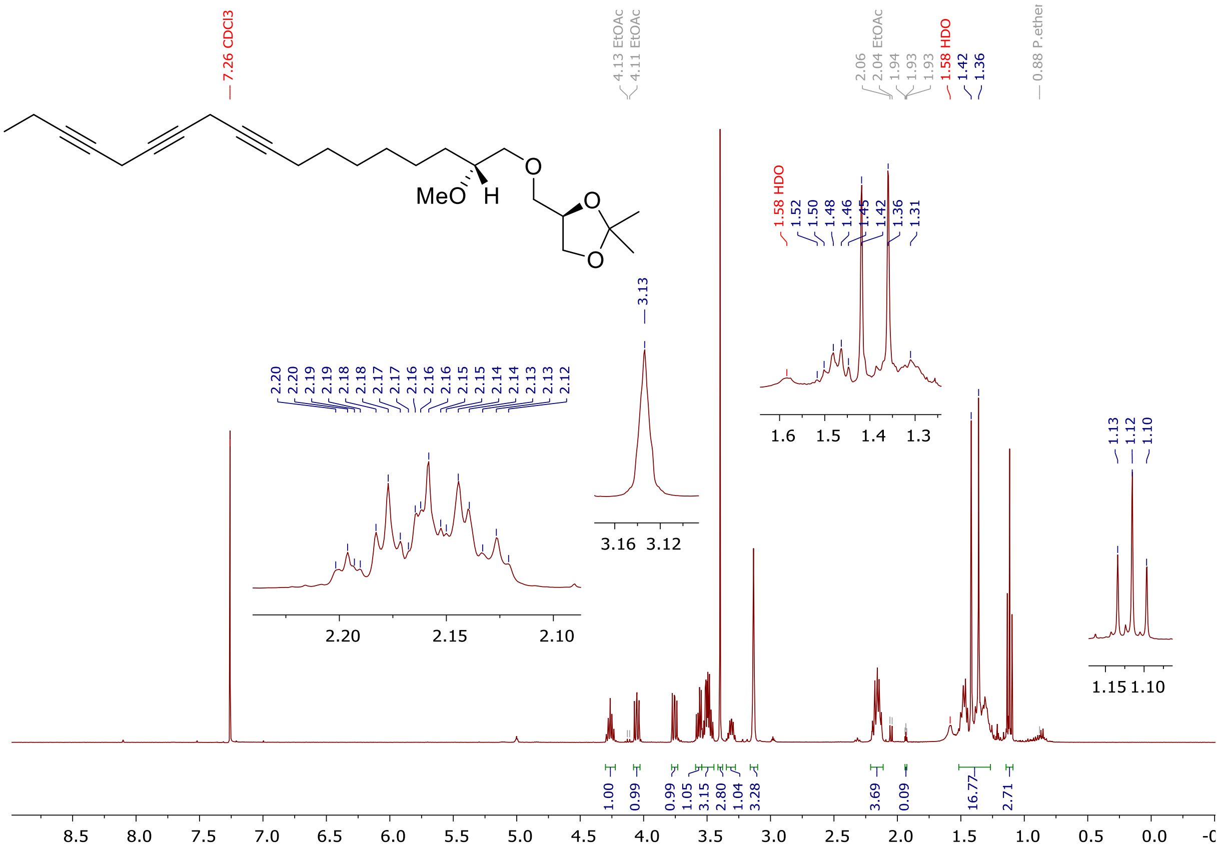

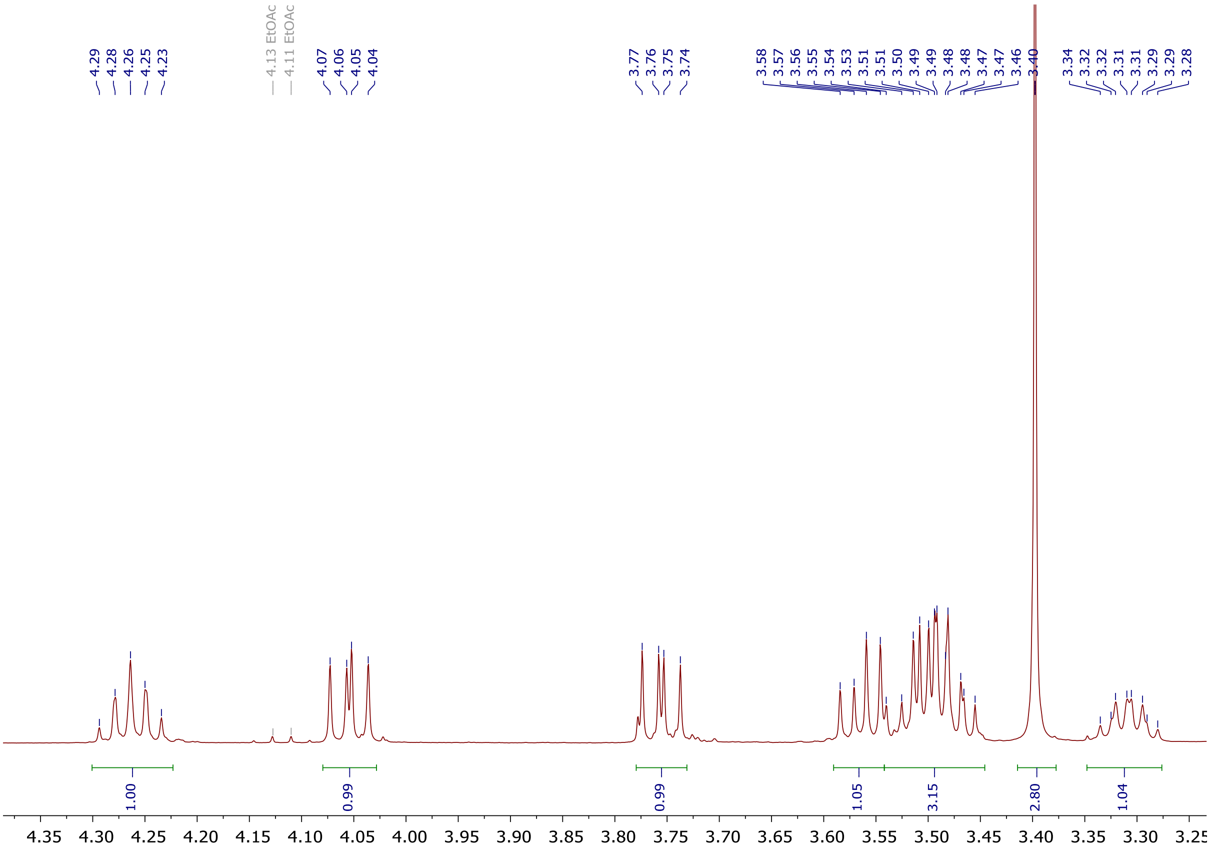

$^{13}C\{H\}$ NMR (101 MHz, $CDCl_3$) of compound **12**

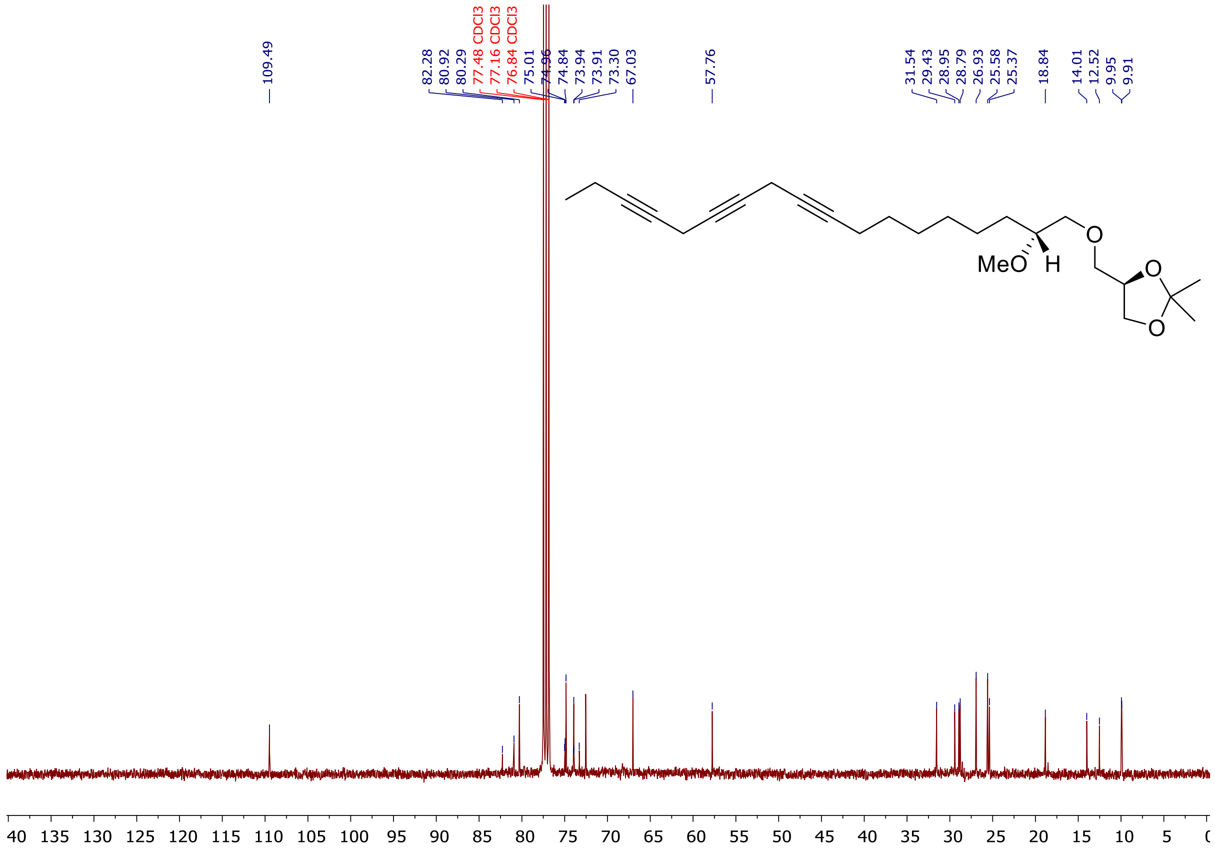

^{1}H-^{1}H COSY spectrum of compound **12**

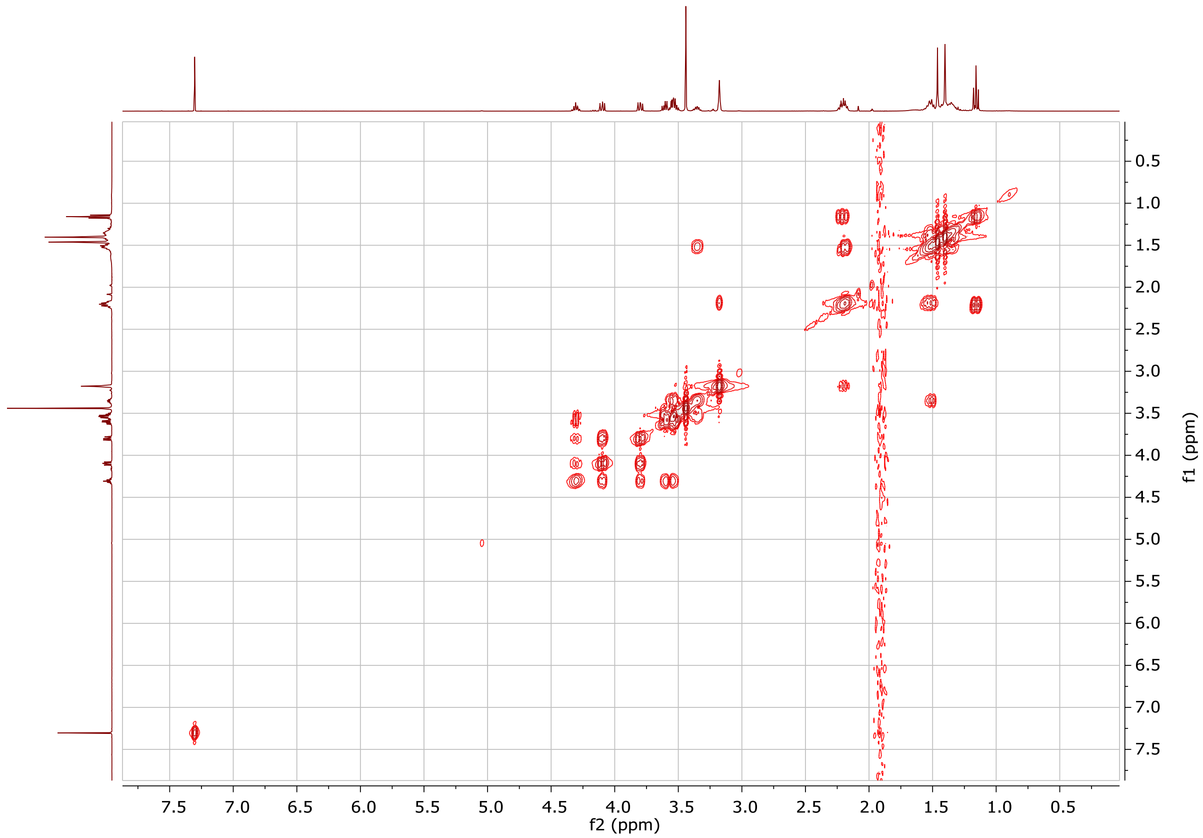

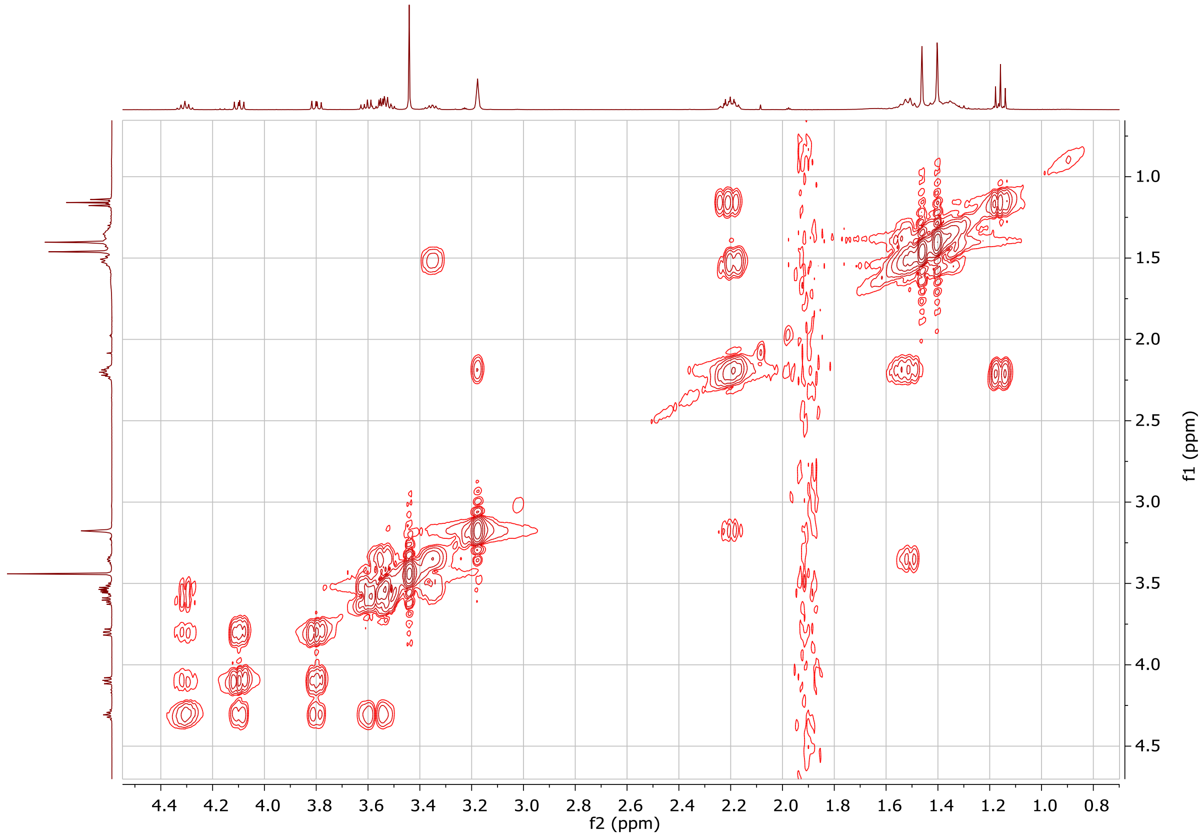

^{1}H NMR (400 MHz, $CDCl_3$) of compound **13**

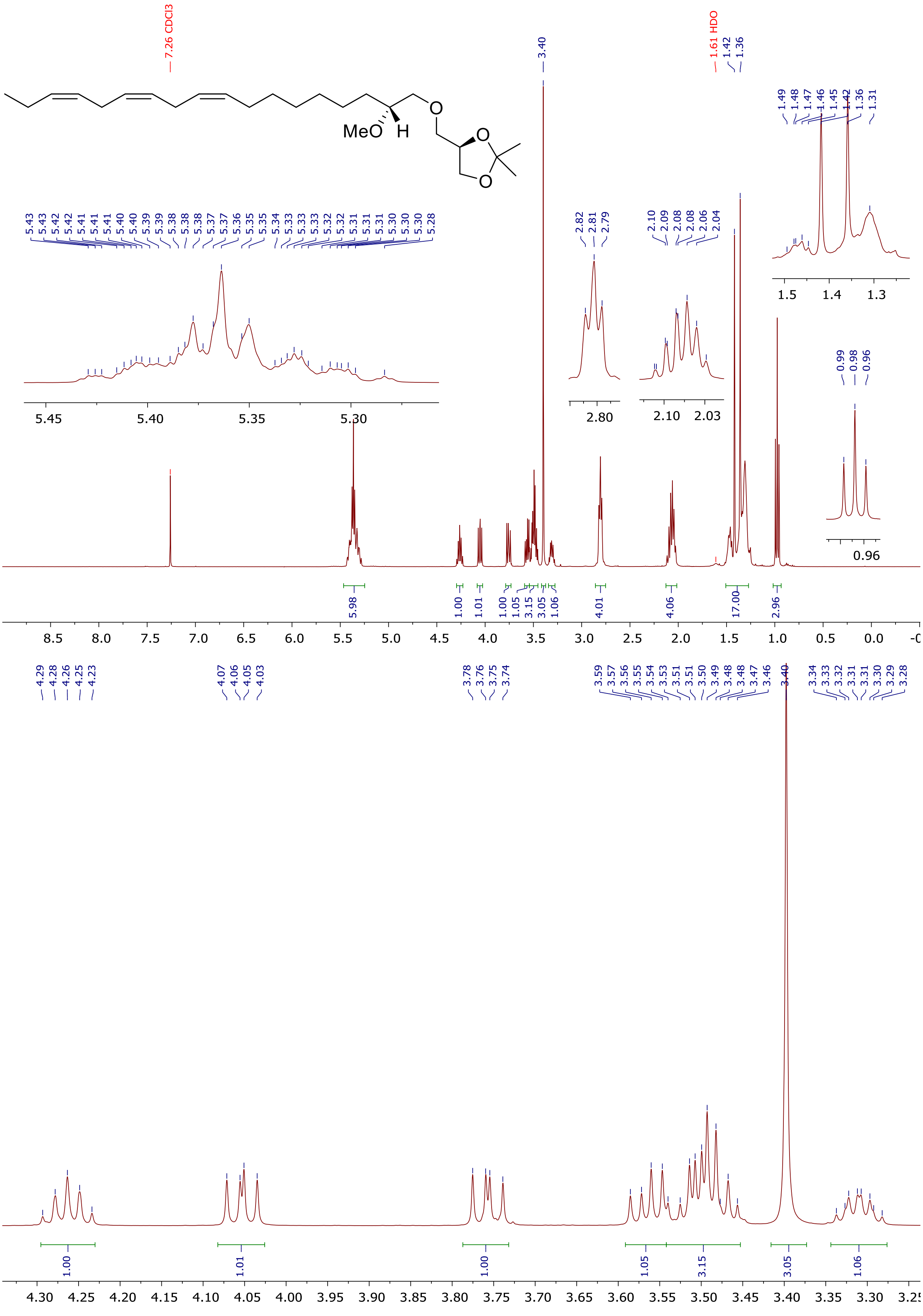

$^{13}C\{H\}$ NMR (101 MHz, $CDCl_3$) of compound **13**

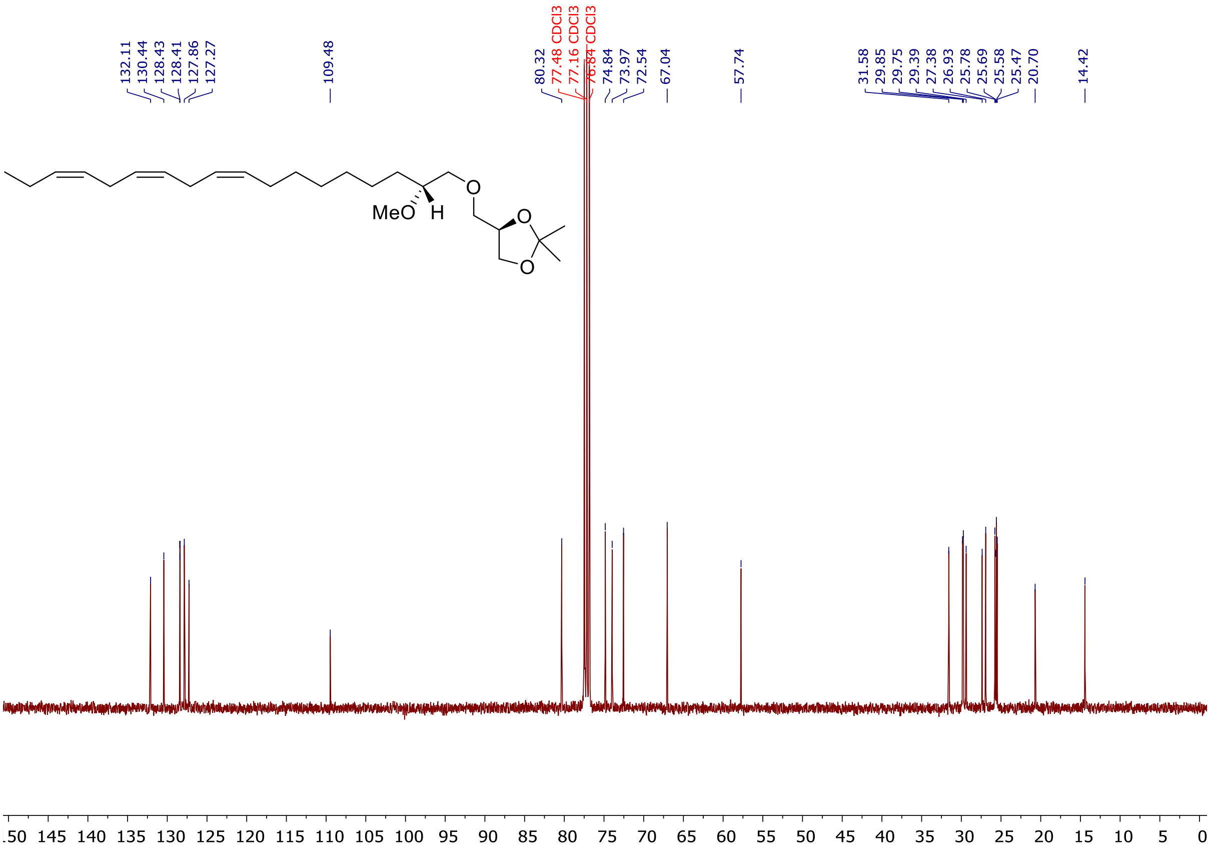

^{1}H-^{1}H COSY spectrum of compound **13**

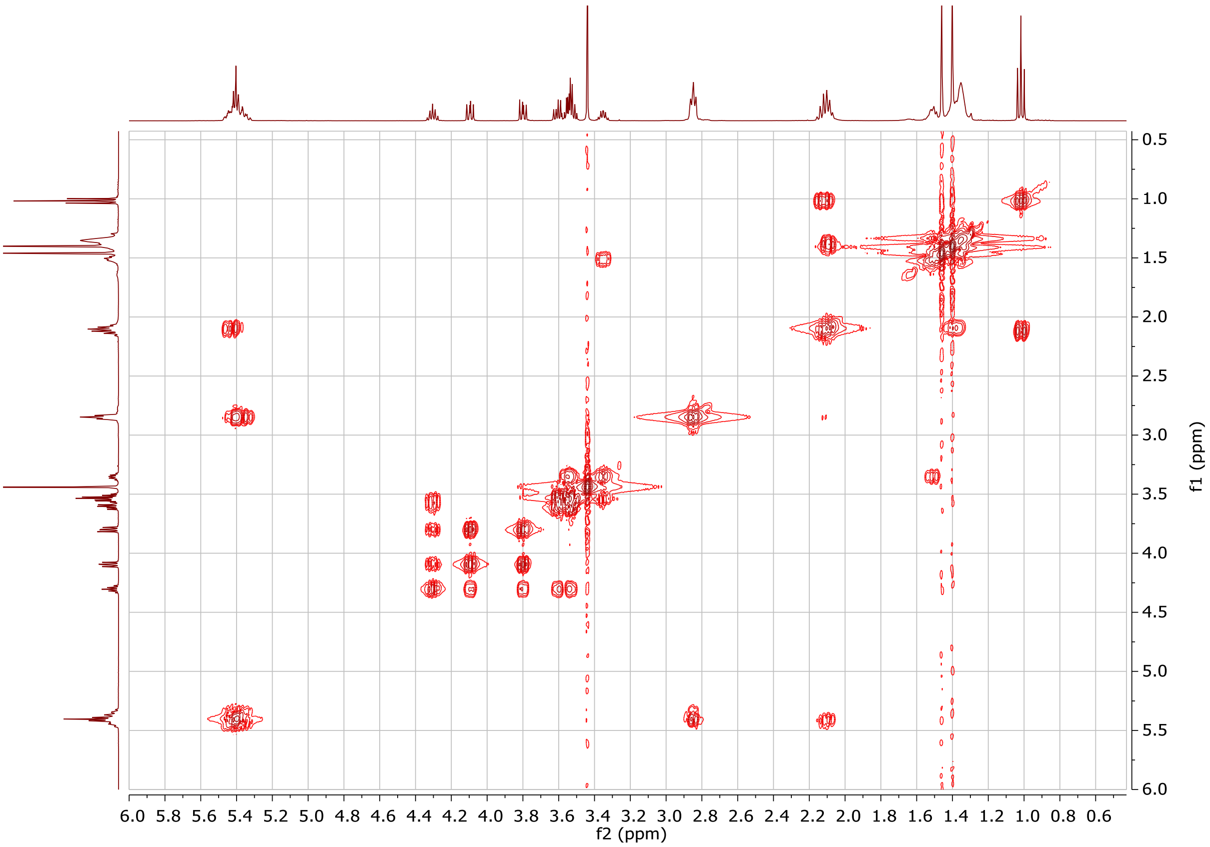

^{13}C-^{1}H HSQC spectrum of compound **13**

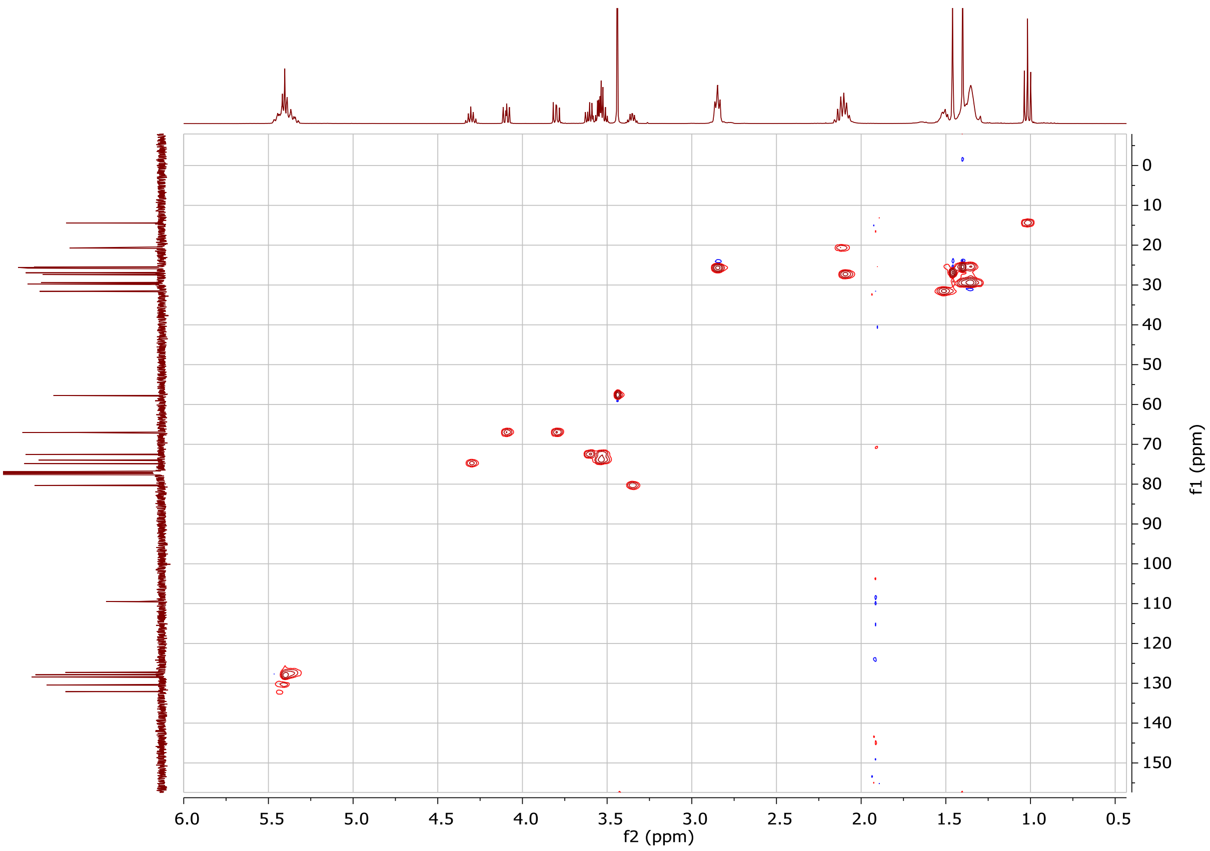

^{1}H NMR (400 MHz, $CDCl_3$) of MEL **5**

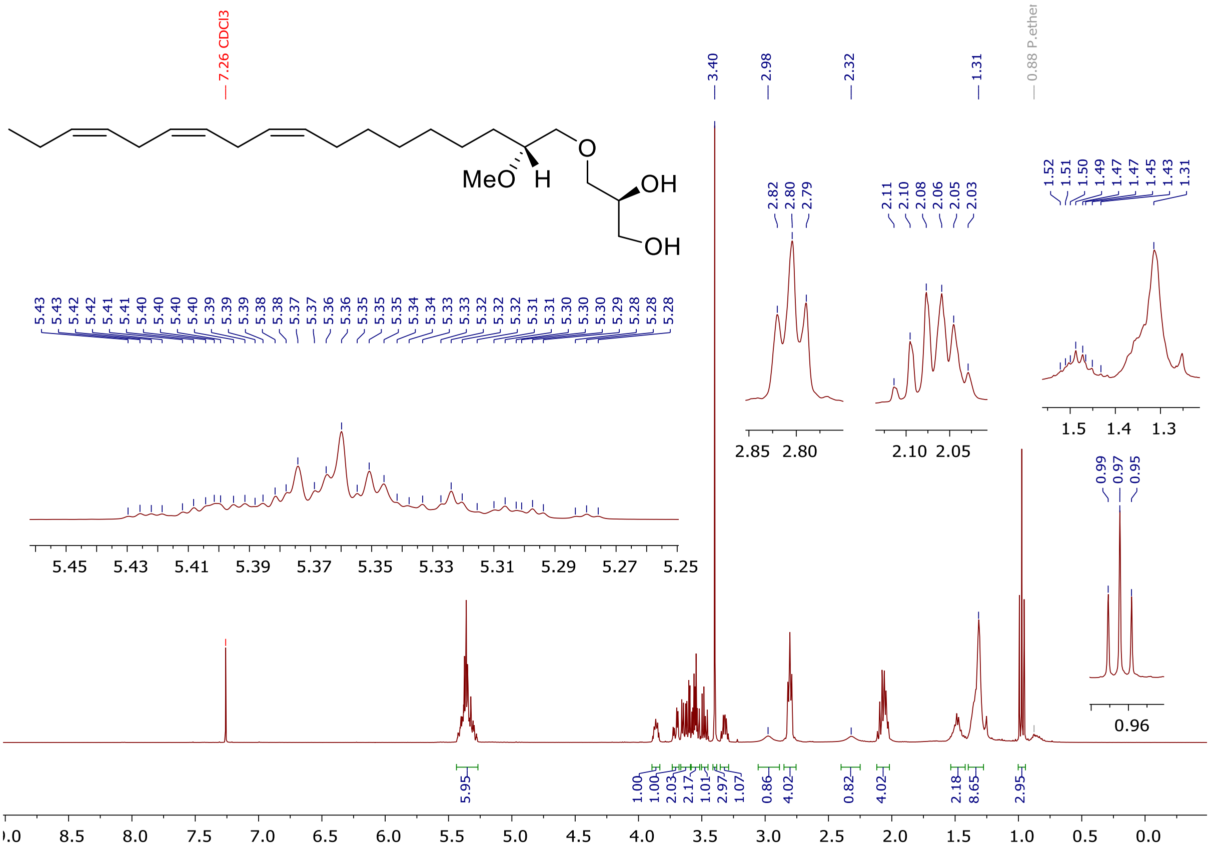

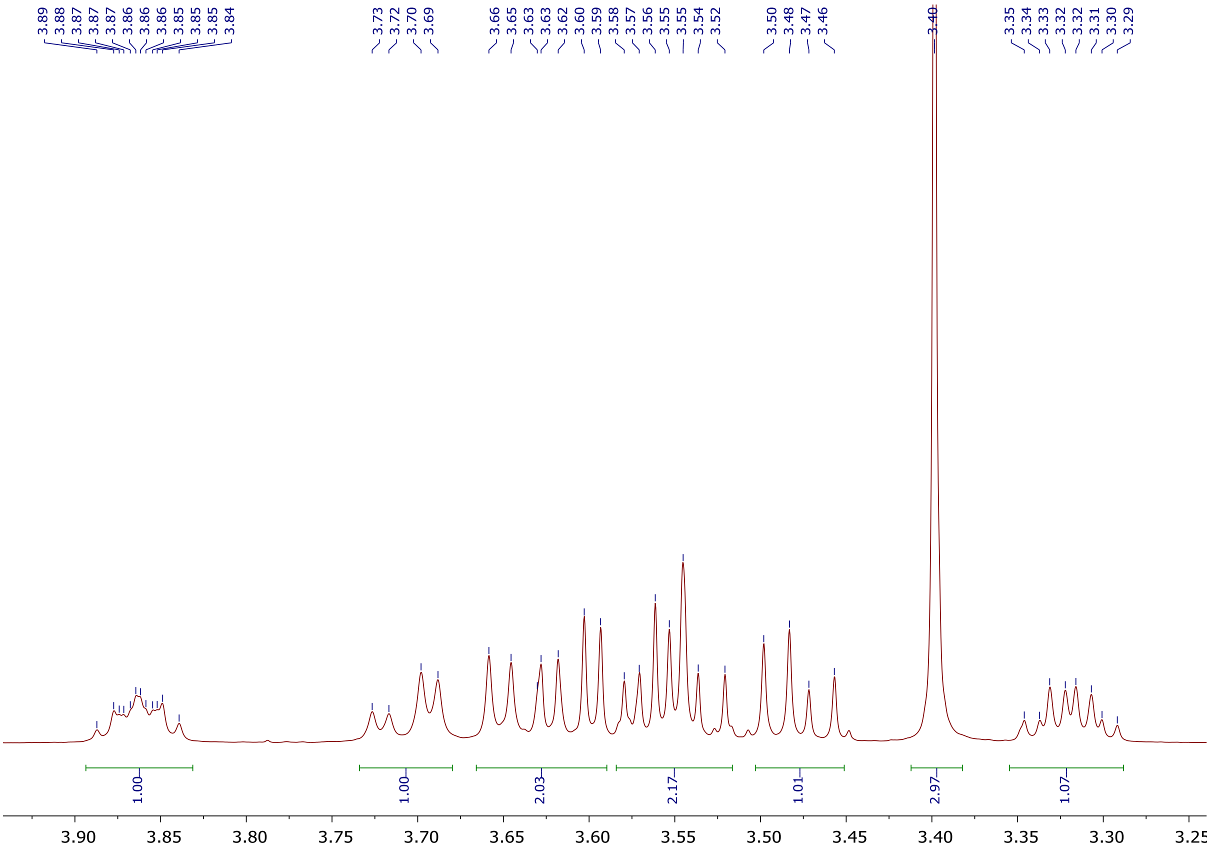

$^{13}C\{H\}$ NMR (101 MHz, $CDCl_3$) of MEL **5**

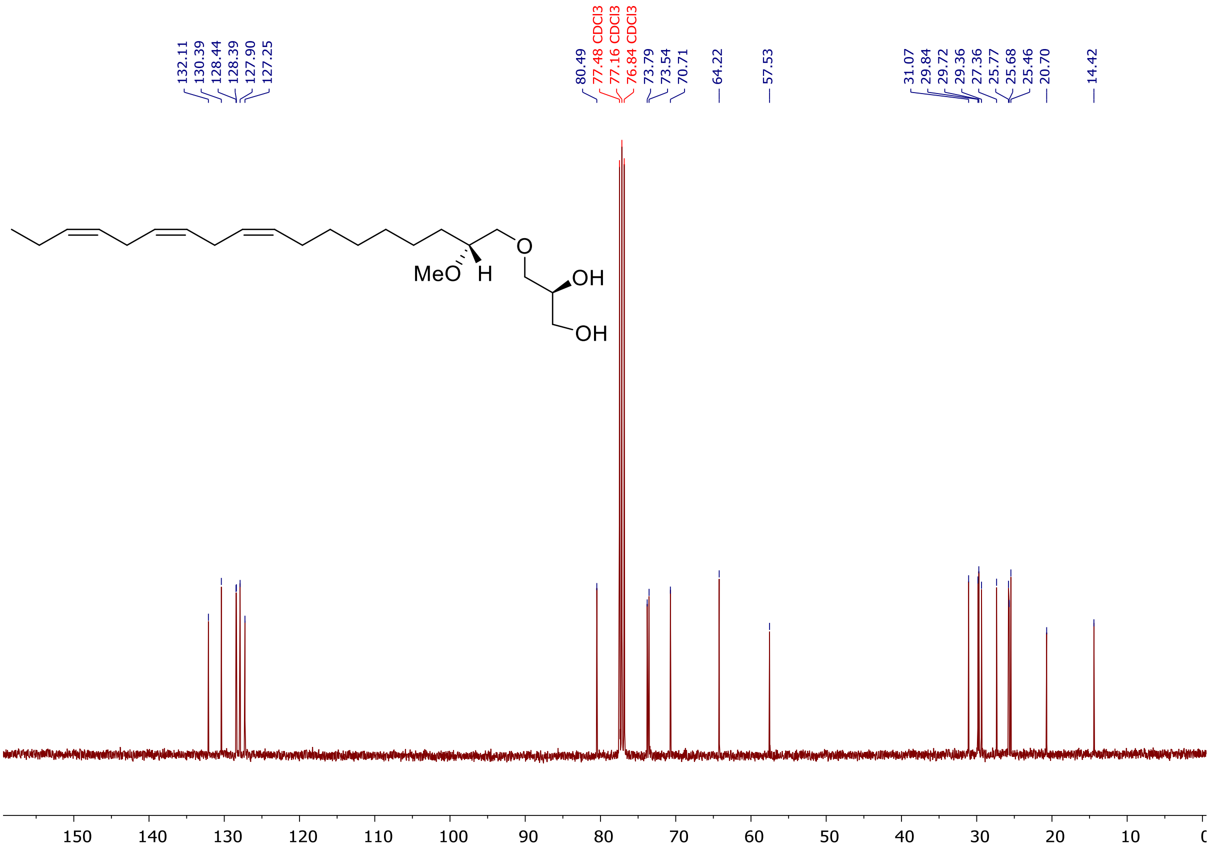

^1H-^1H COSY spectrum of MEL **5**

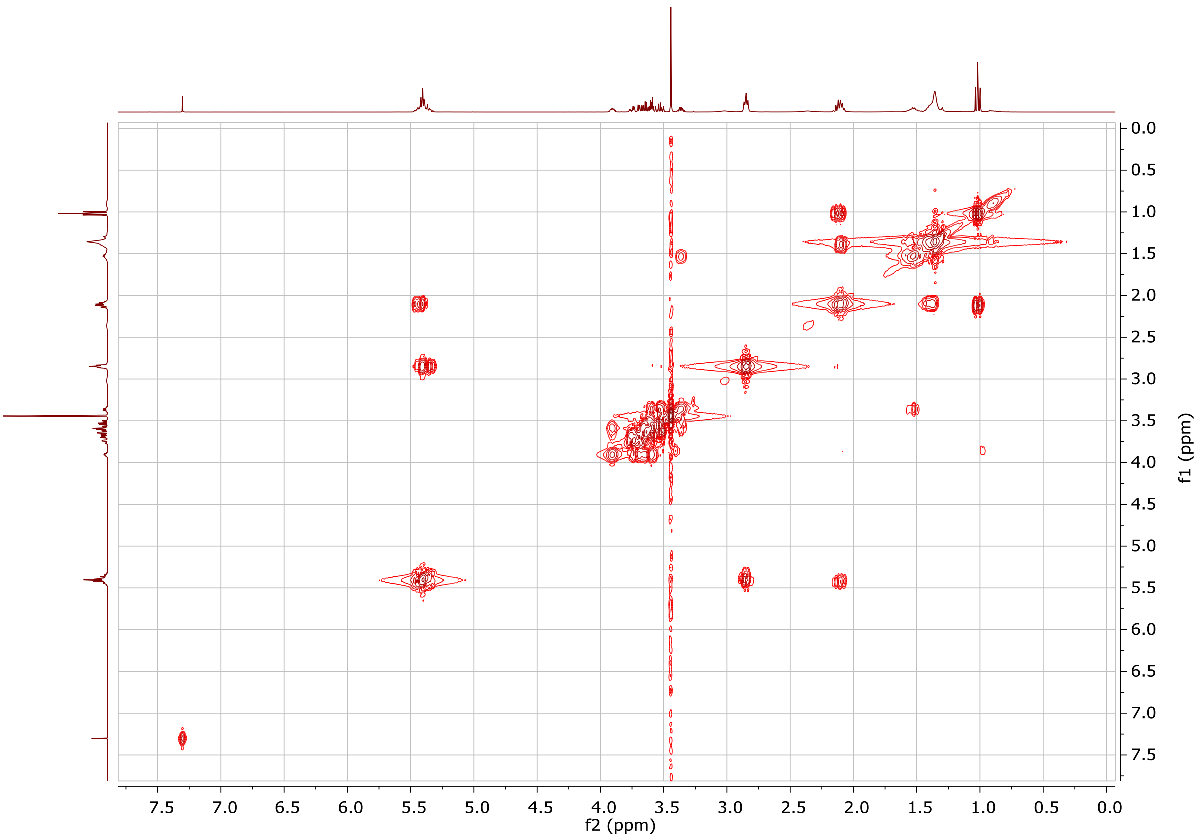

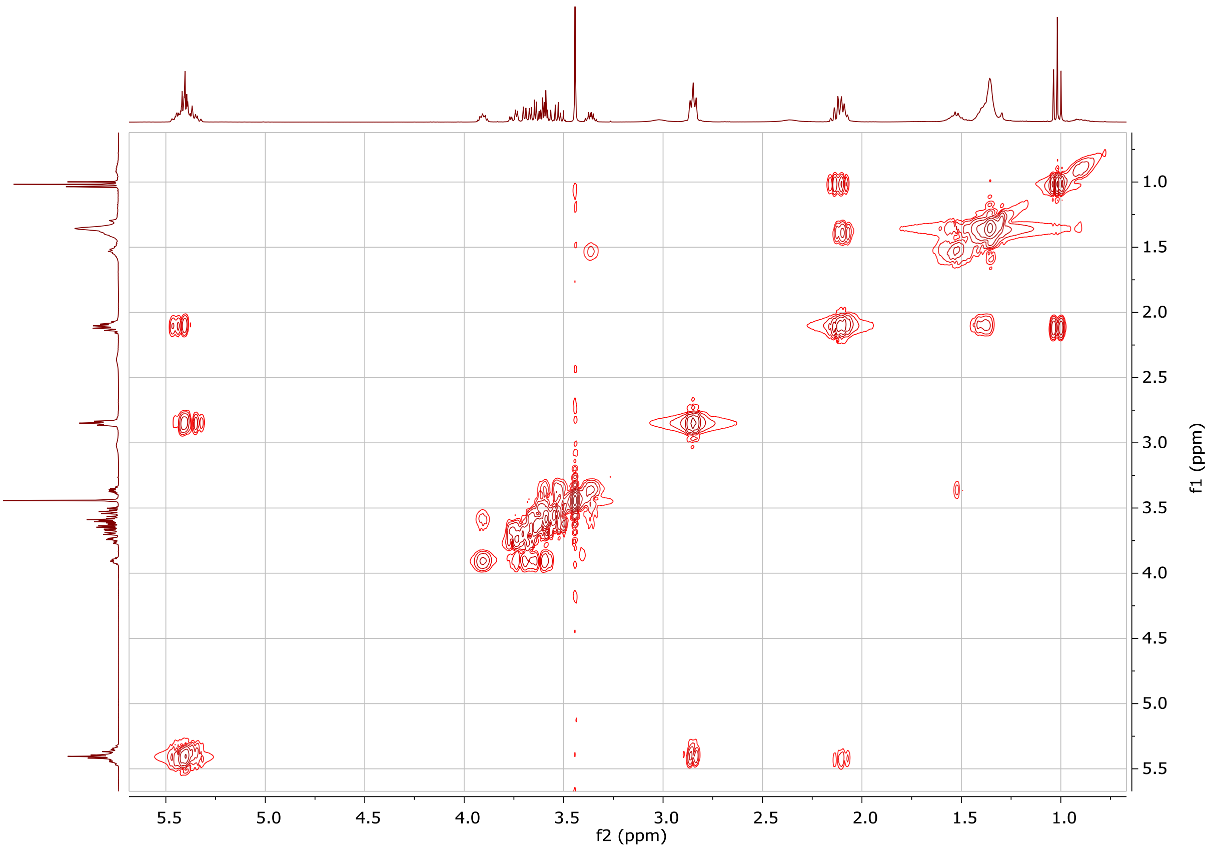

^{13}C-^{1}H HSQC spectrum of MEL **5**

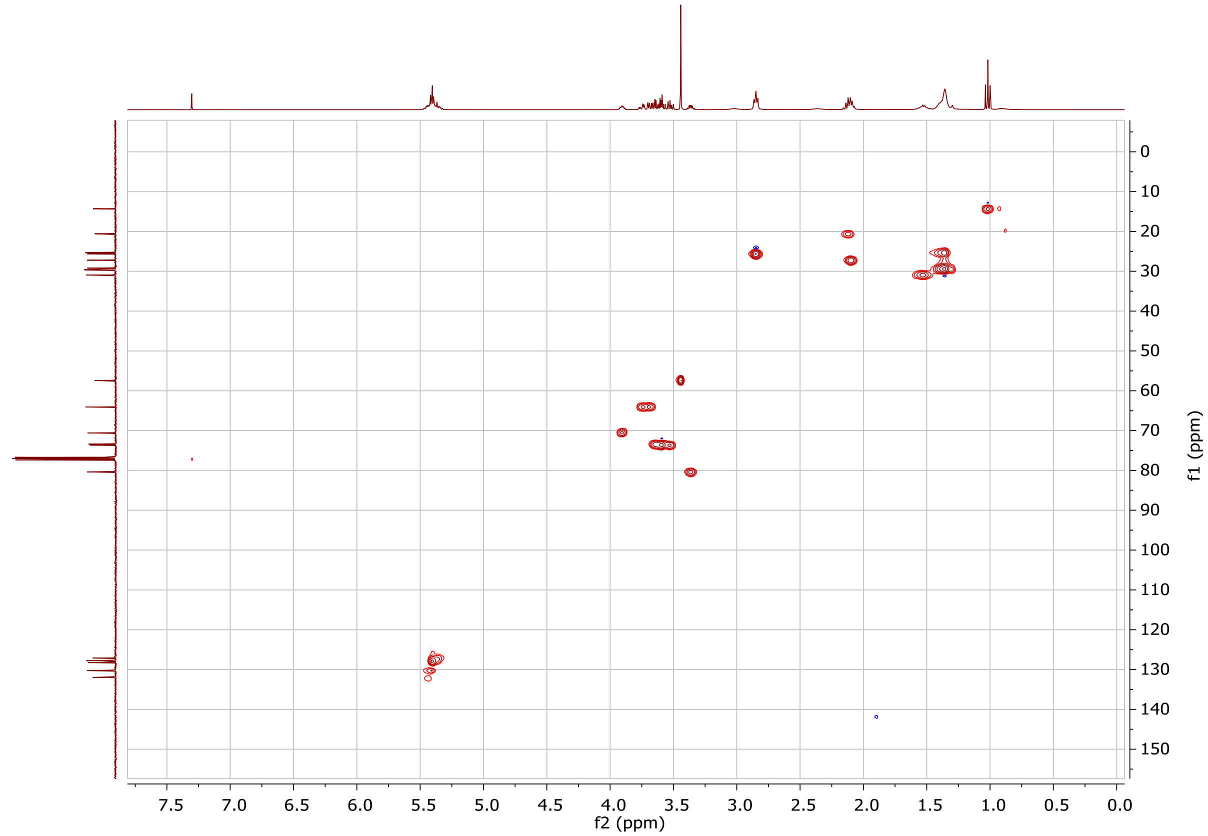

^{1}H NMR (400 MHz, $CDCl_3$) of compound **14**

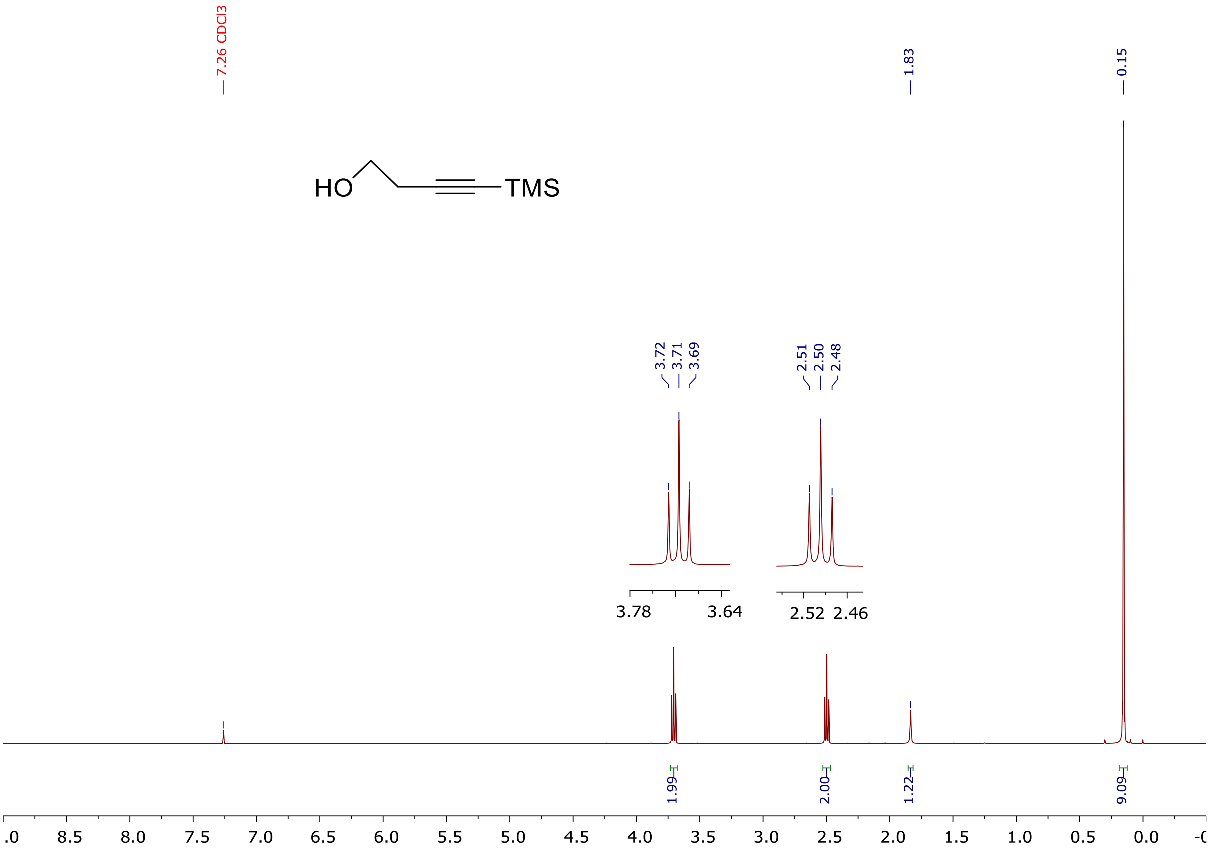

$^{13}C\{H\}$ NMR (101 MHz, $CDCl_3$) of compound **14**

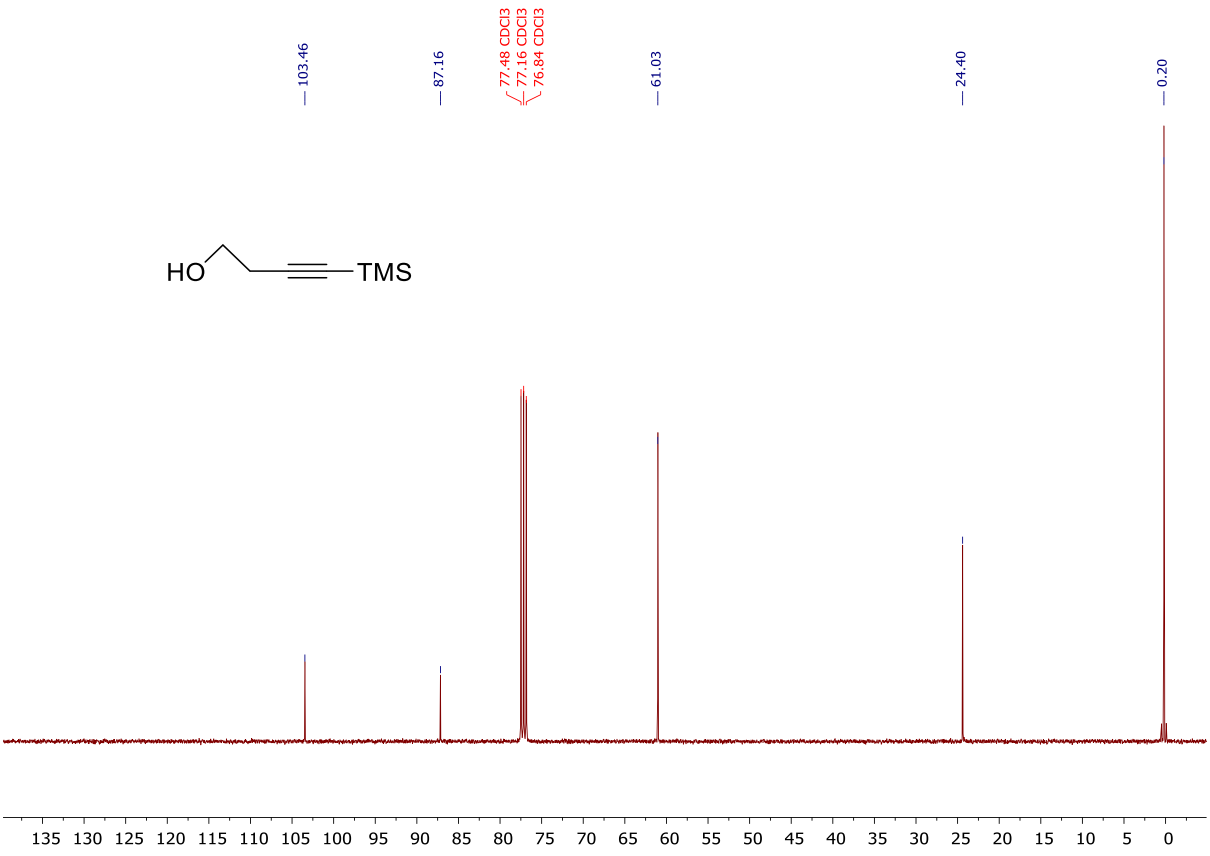

^{1}H-^{1}H COSY spectrum of compound **14**

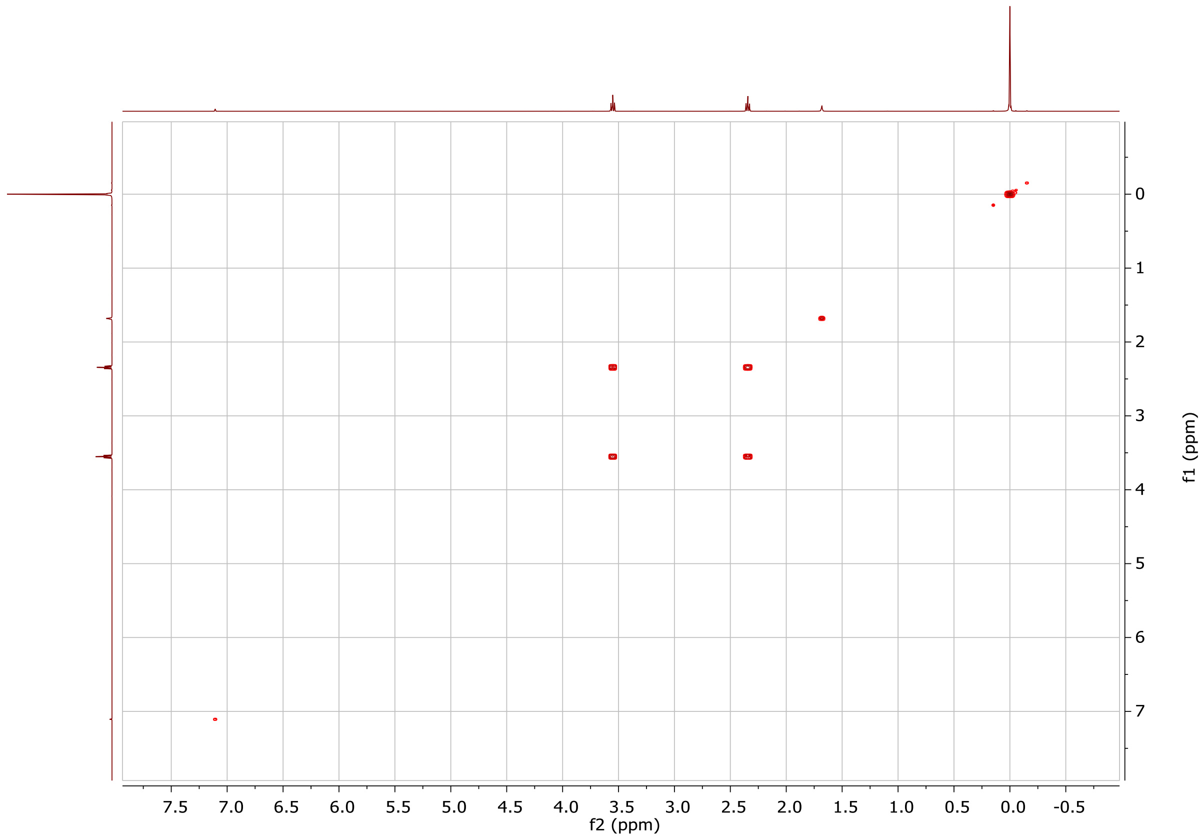

^{13}C-^{1}H HSQC spectrum of compound **14**

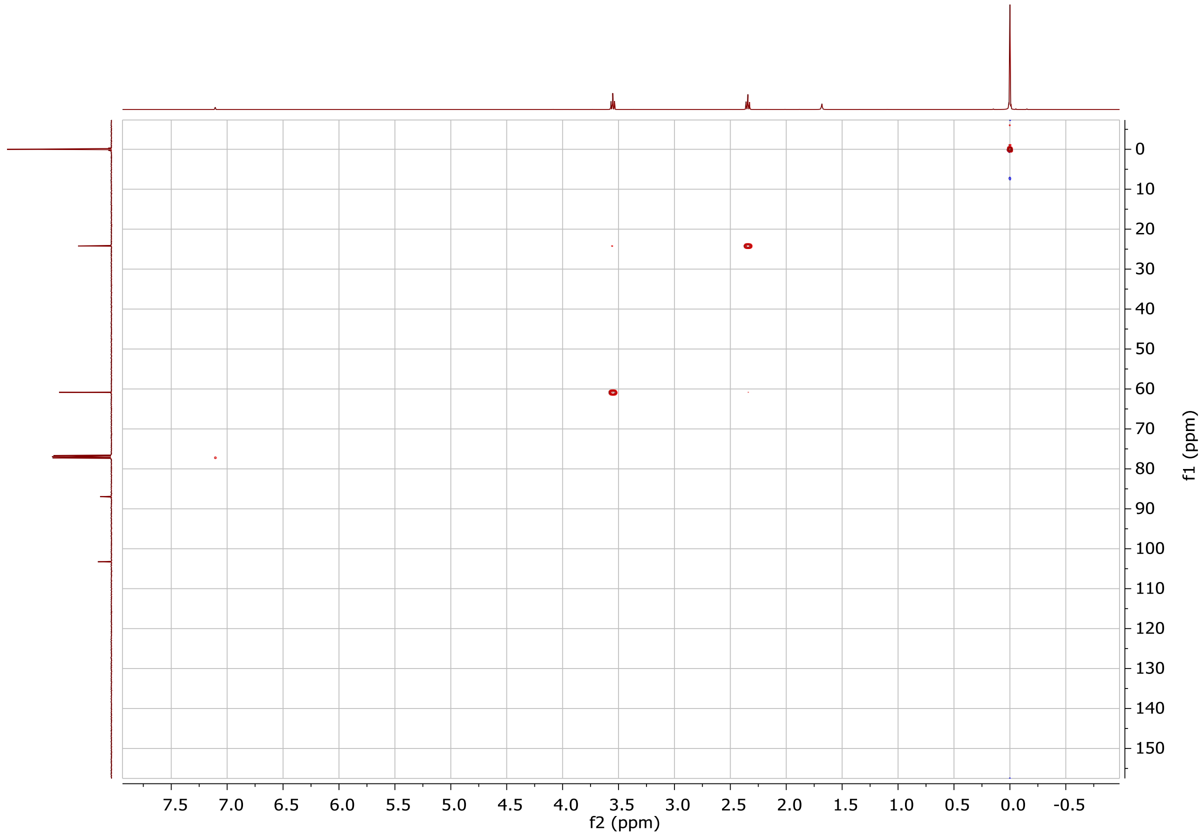

^{1}H NMR (400 MHz, $CDCl_3$) of compound **15**

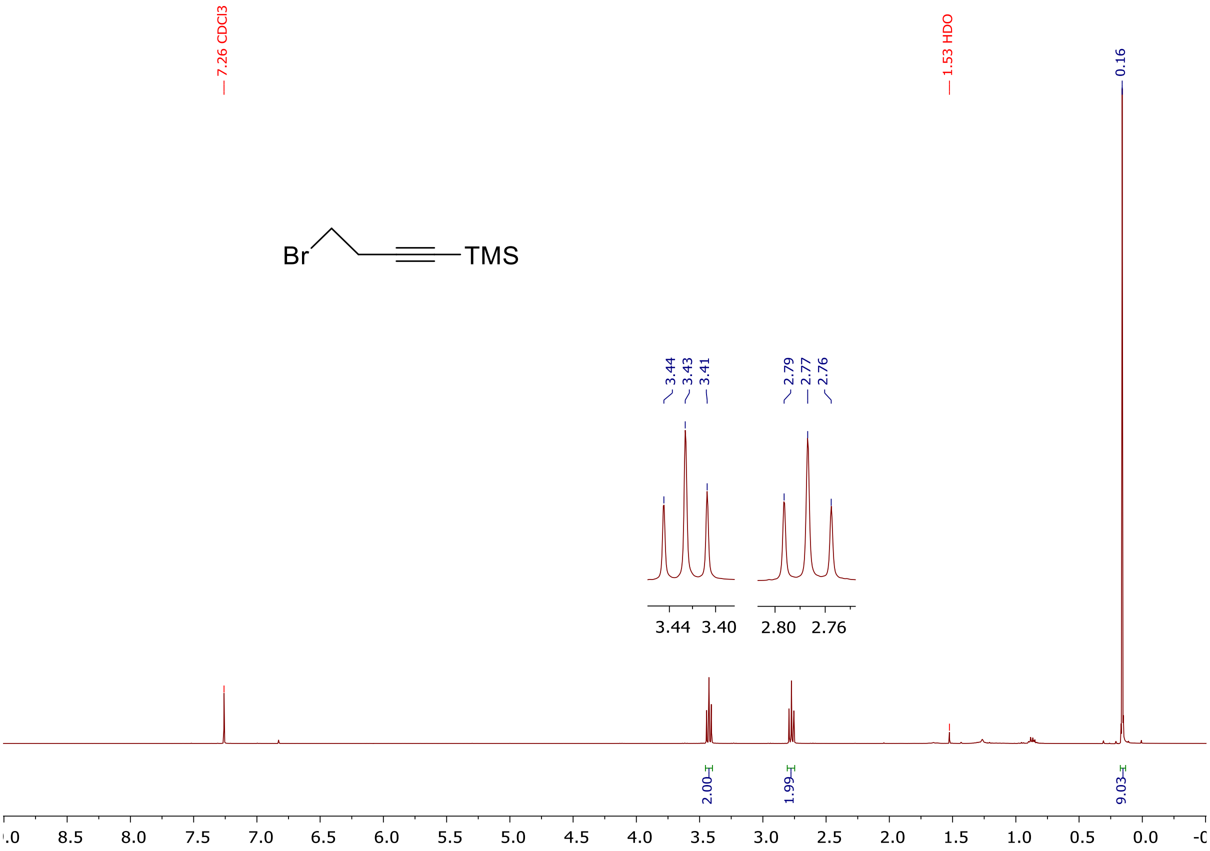

^{13}C{H} NMR (101 MHz, $CDCl_3$) of compound **15**

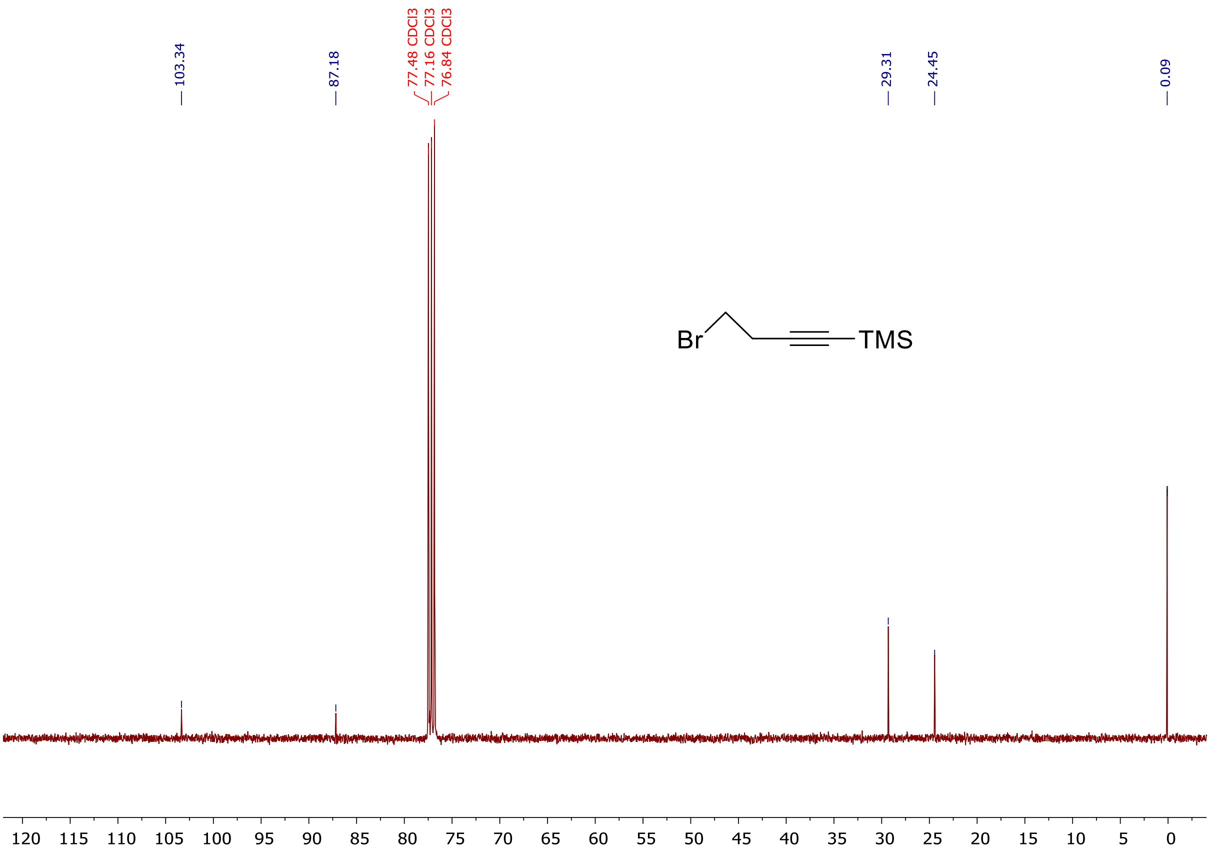

^1H NMR (400 MHz, $CDCl_3$) of compound **16**

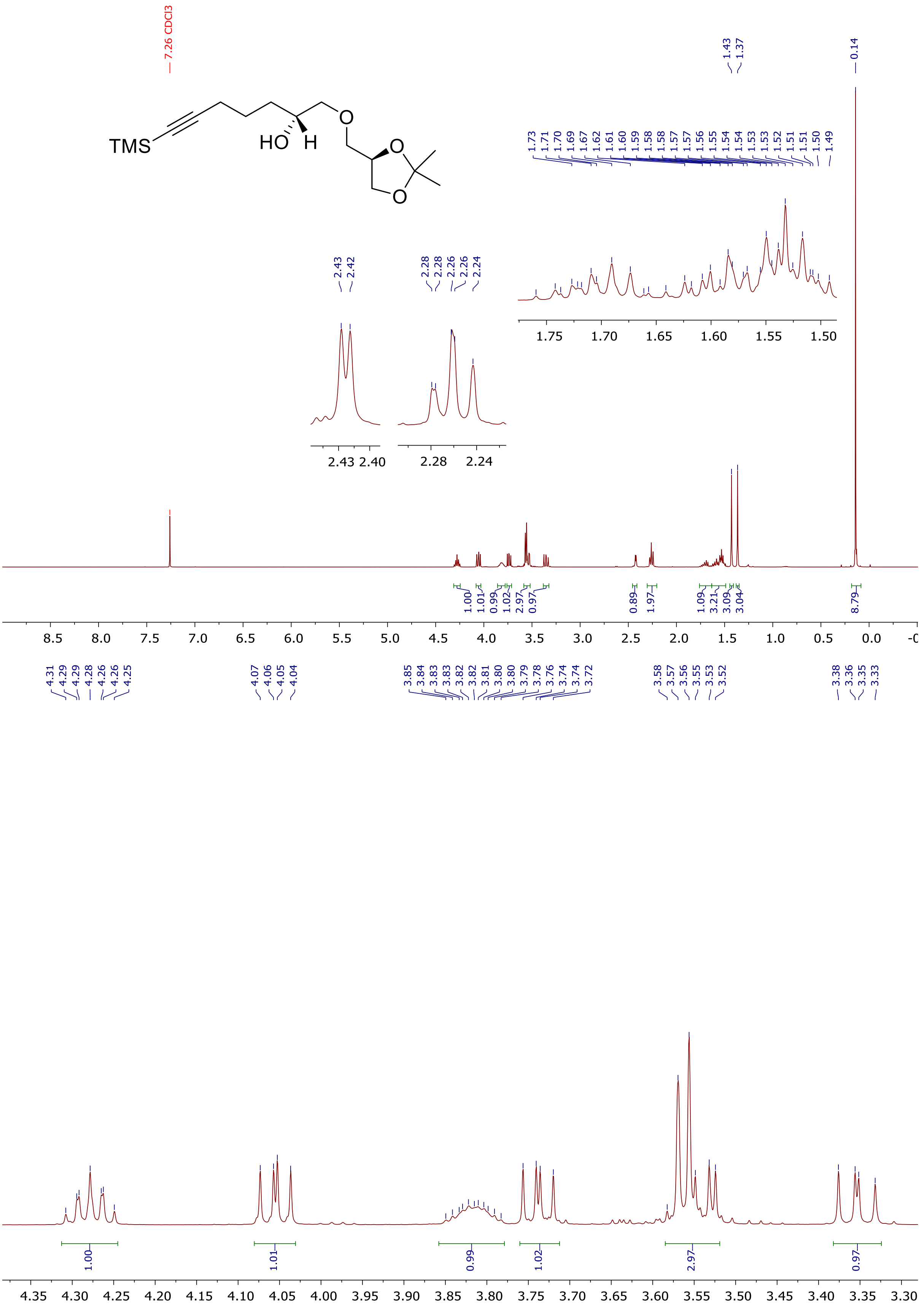

$^{13}C\{H\}$ NMR (101 MHz, $CDCl_3$) of compound **16**

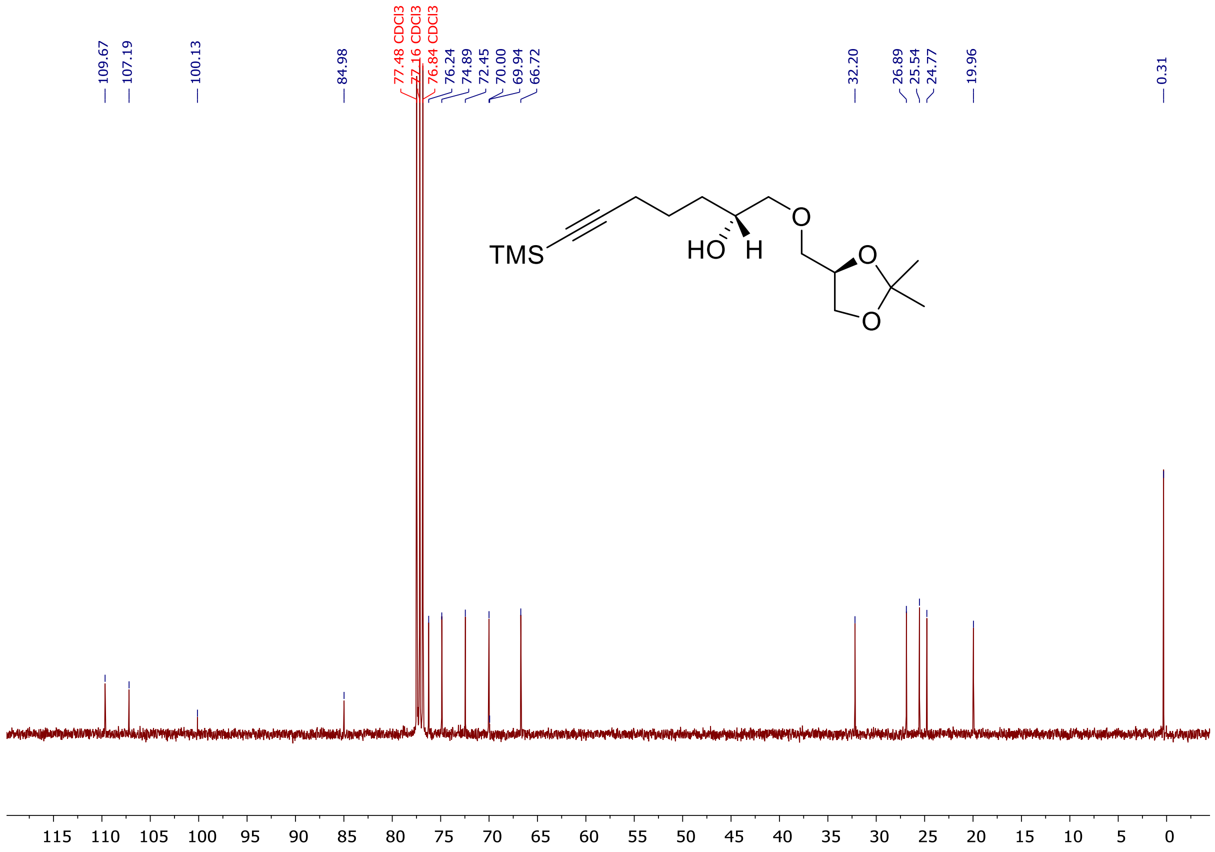

^{1}H-^{1}H COSY spectrum of compound **16**

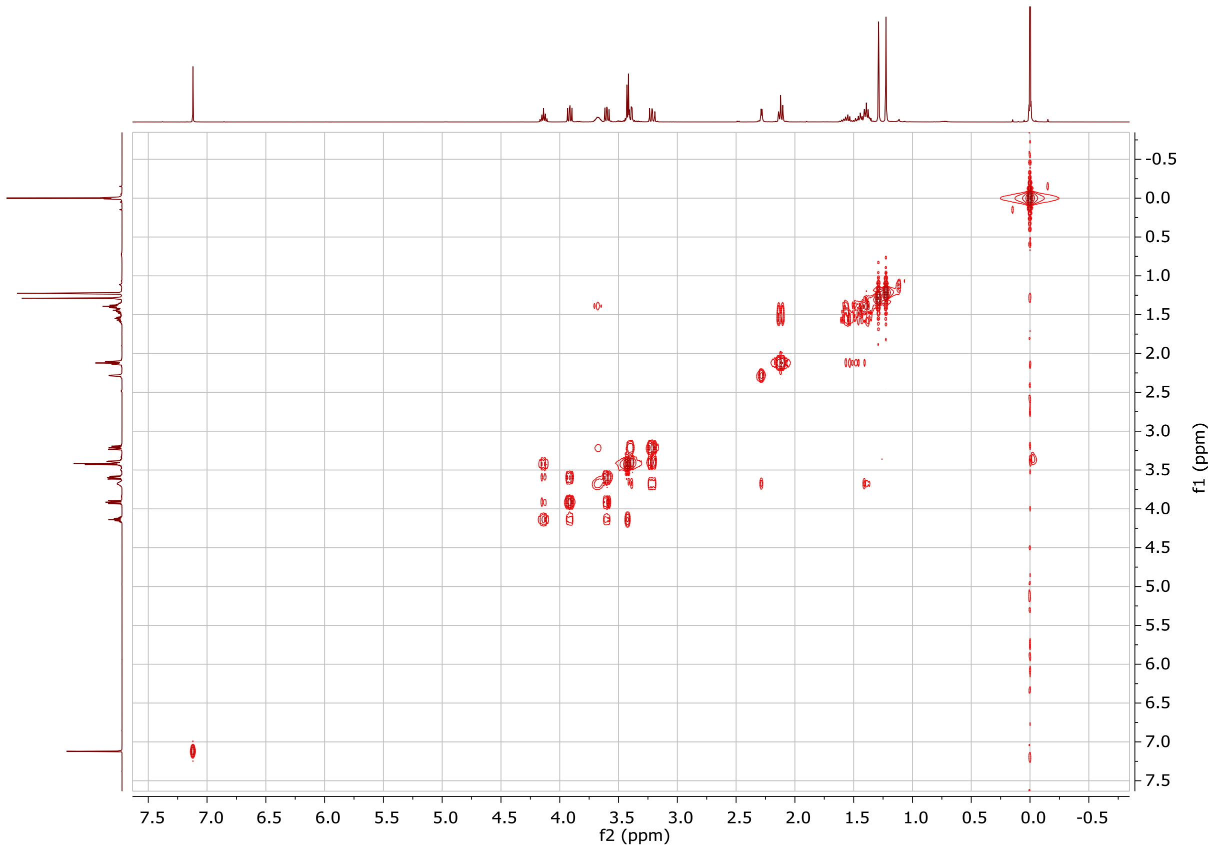

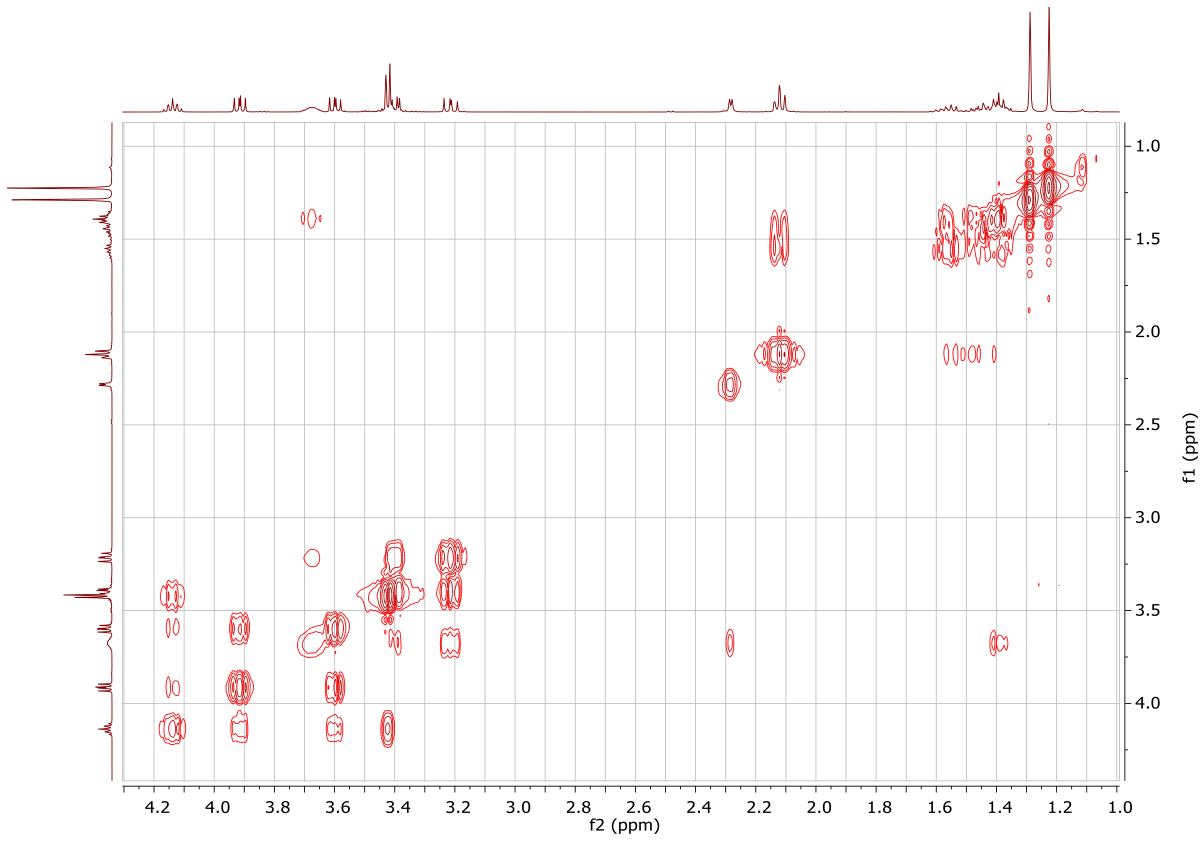

^{13}C-^{1}H HSQC spectrum of compound **16**

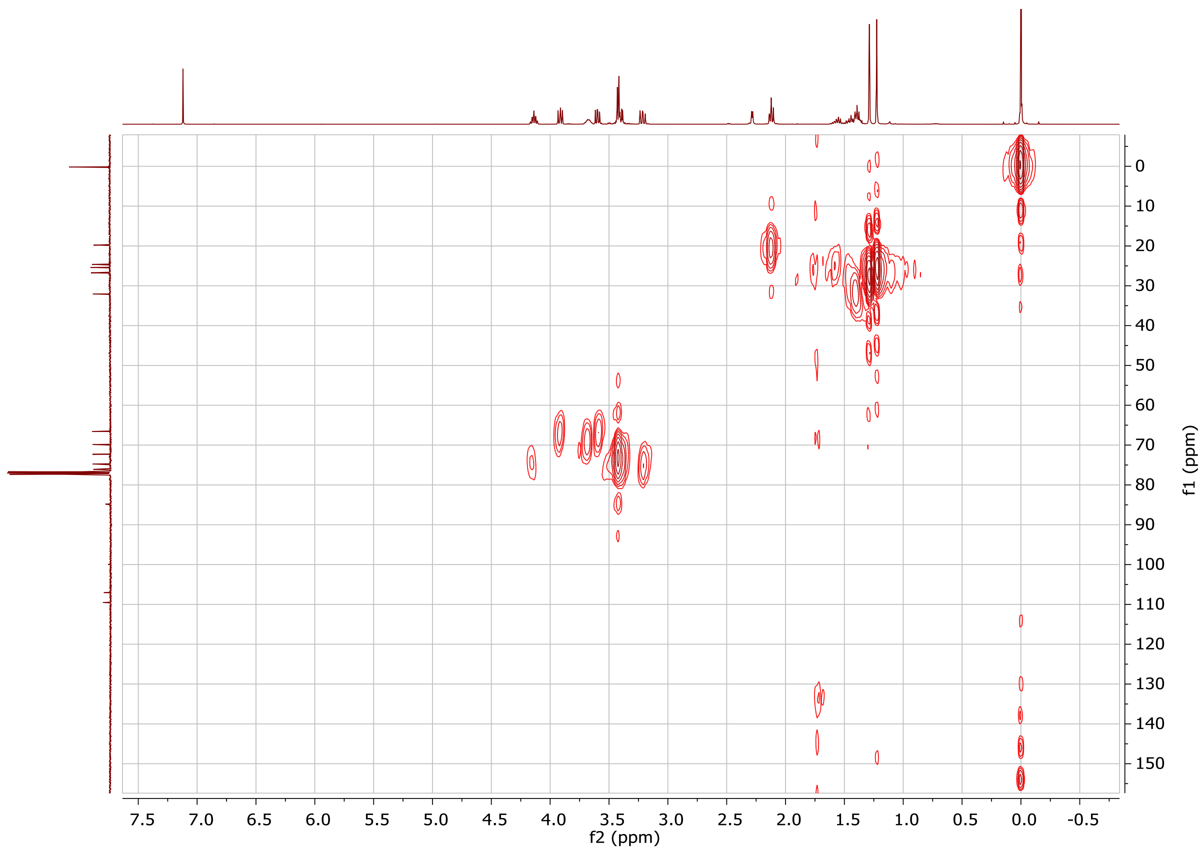

^{1}H NMR (400 MHz, $CDCl_3$) of compound **17**

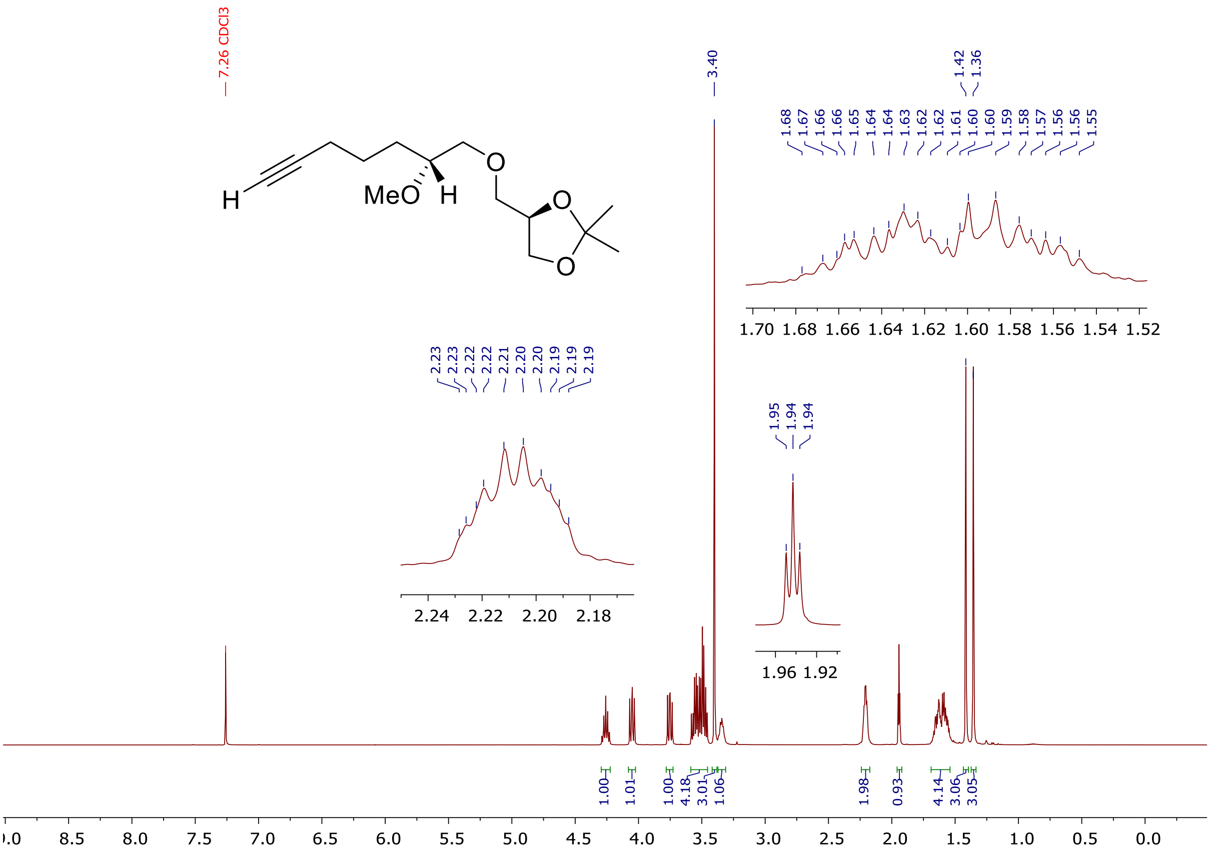

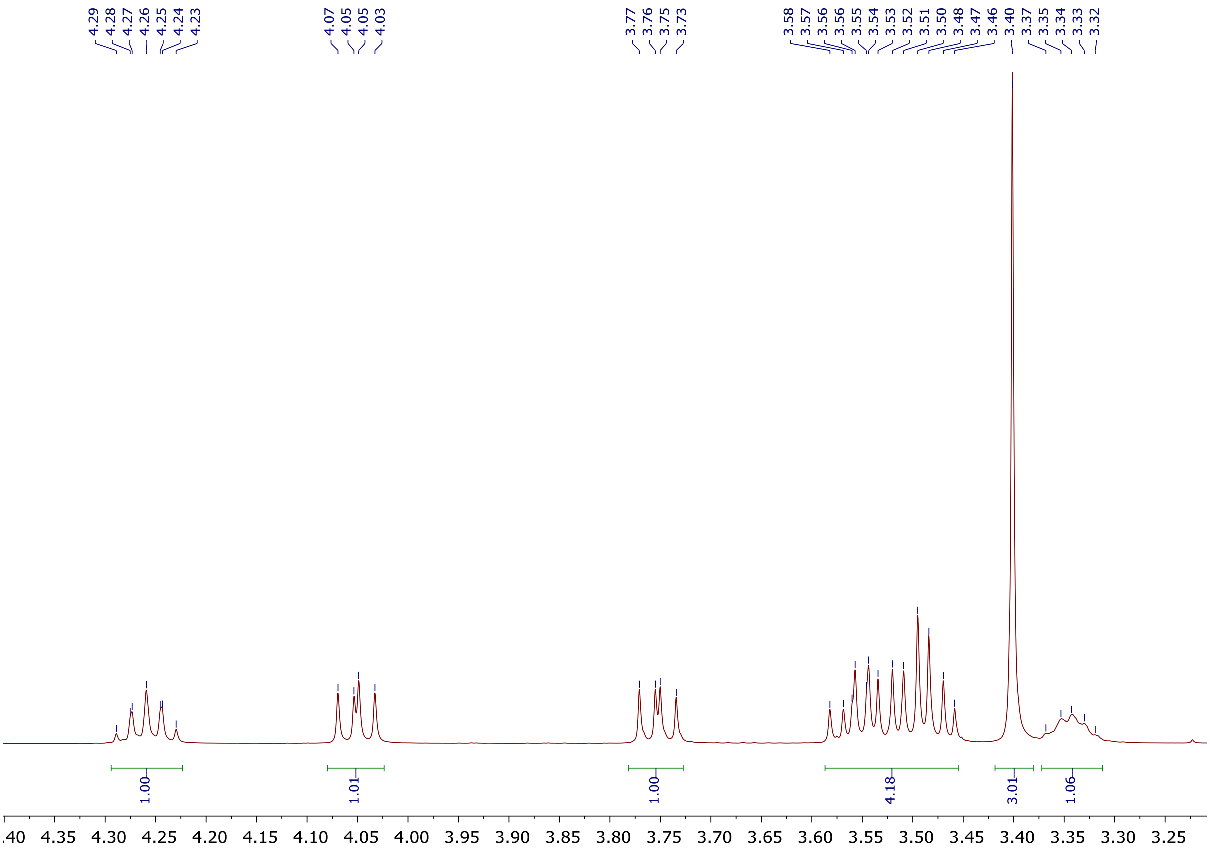

$^{13}C\{H\}$ NMR (101 MHz, $CDCl_3$) of compound **17**

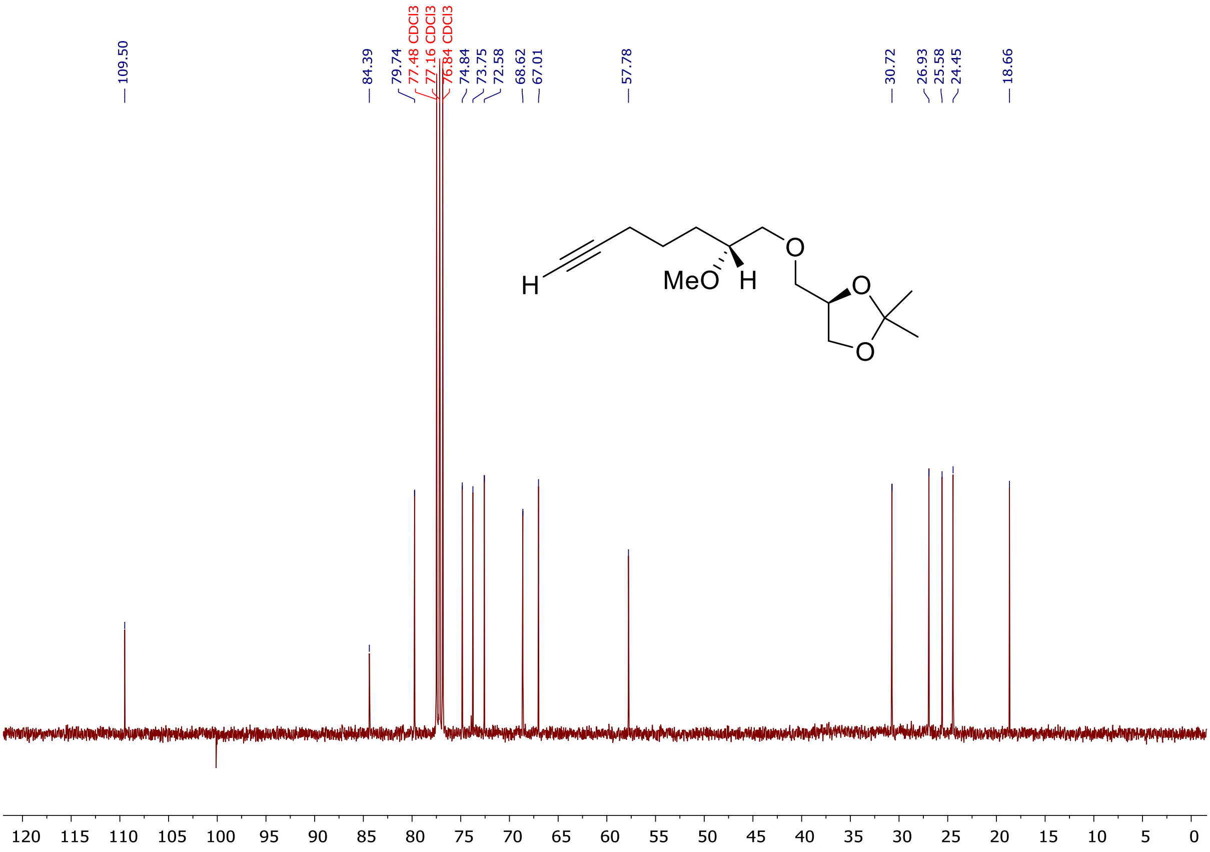

^1H-^1H COSY spectrum of compound **17**

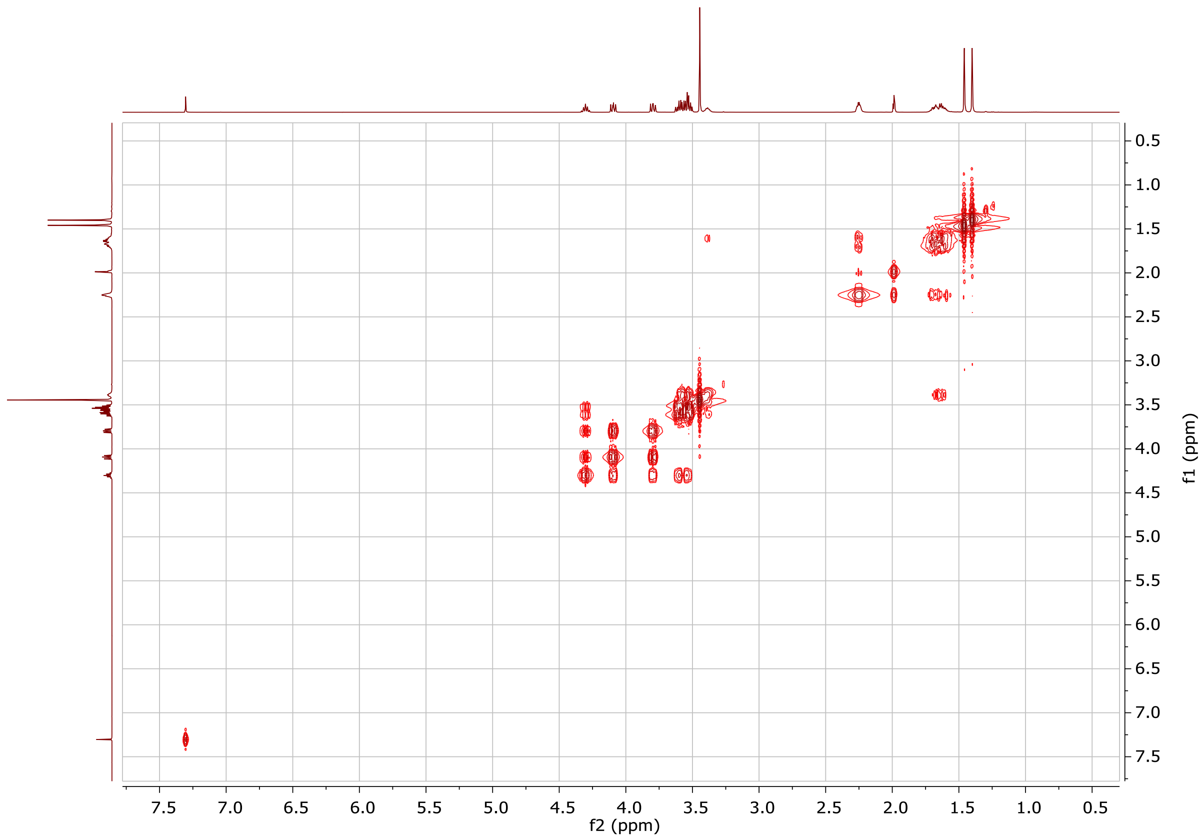

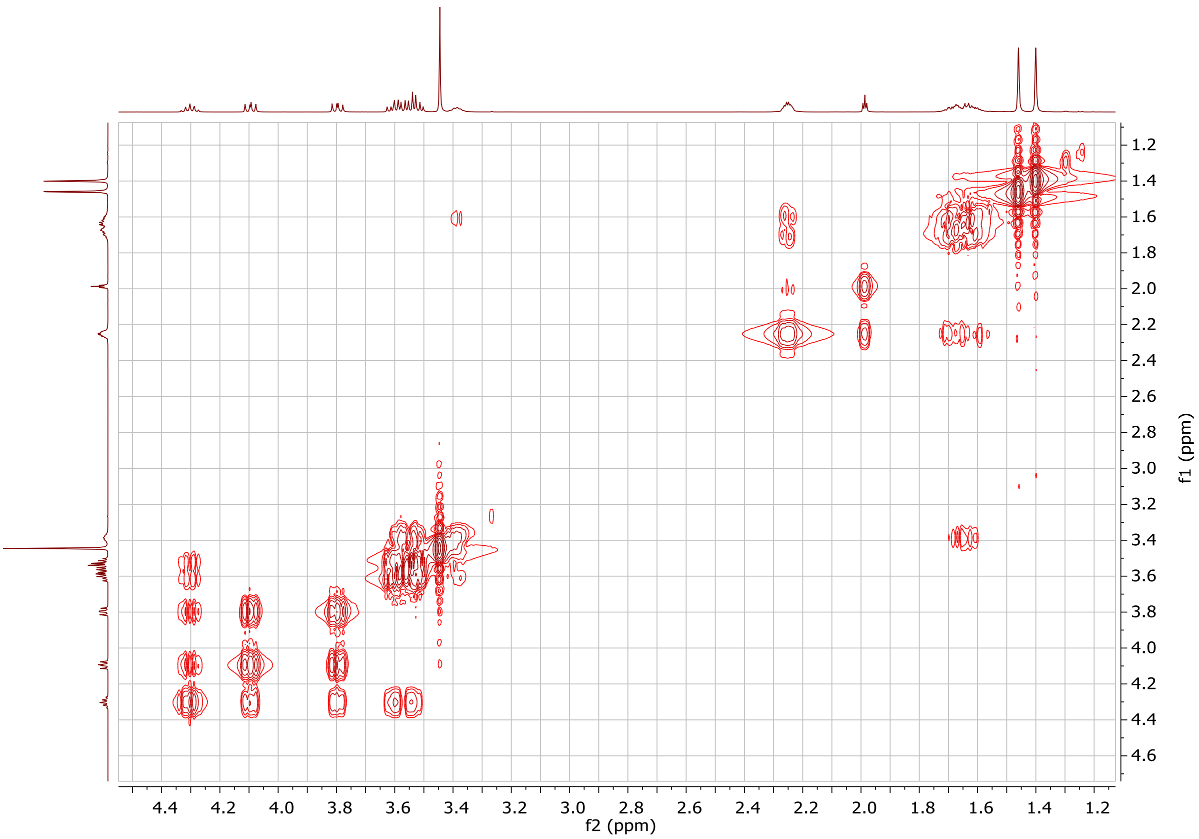

^{13}C-^{1}H HSQC spectrum of compound **17**

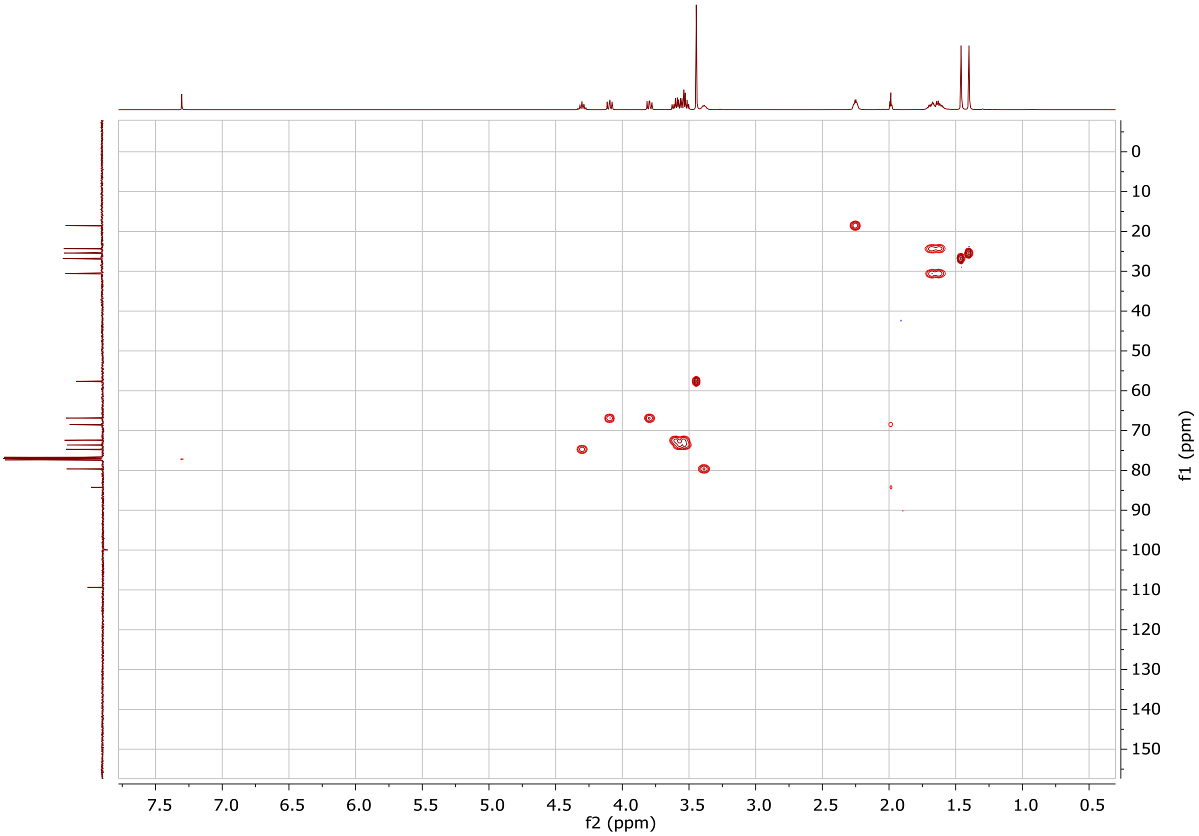

^{1}H NMR (400 MHz, $CDCl_3$) of compound **18**

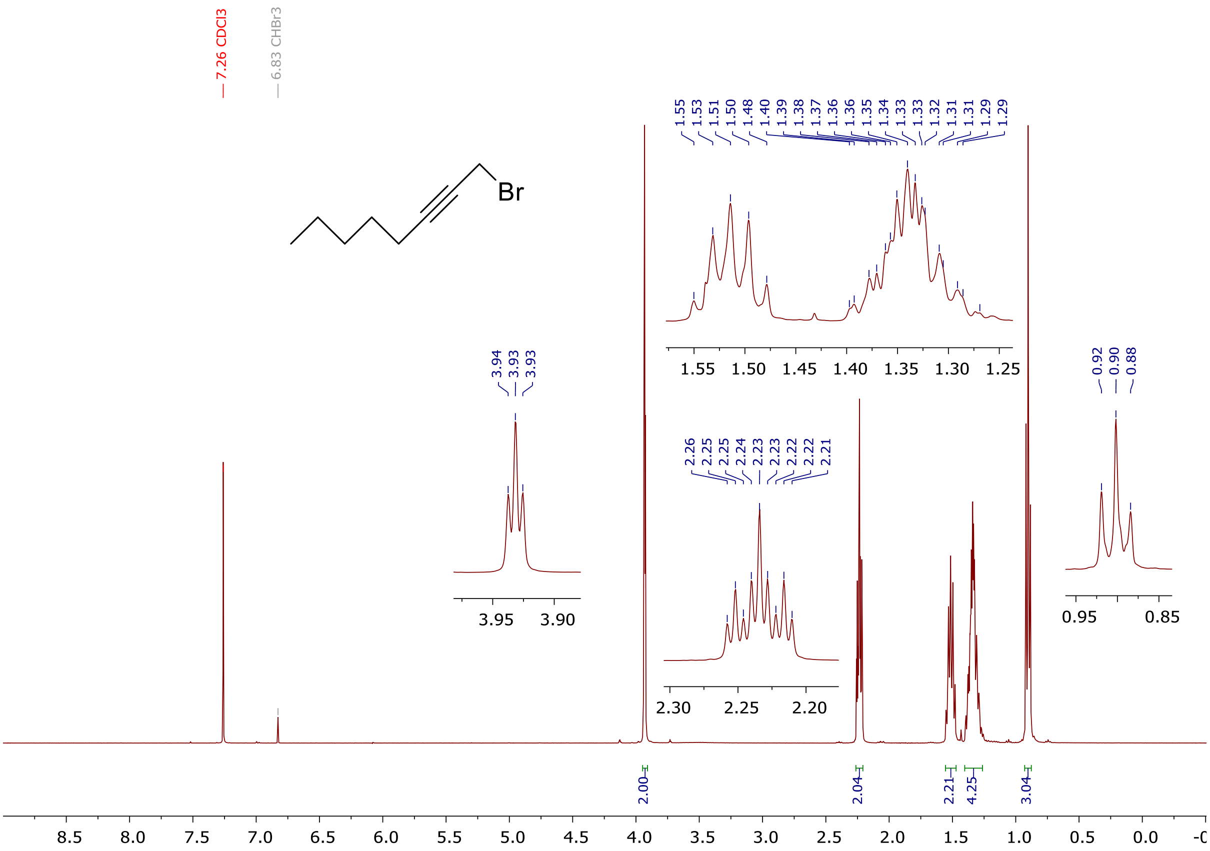

^{13}C{H} NMR (101 MHz, $CDCl_3$) of compound **18**

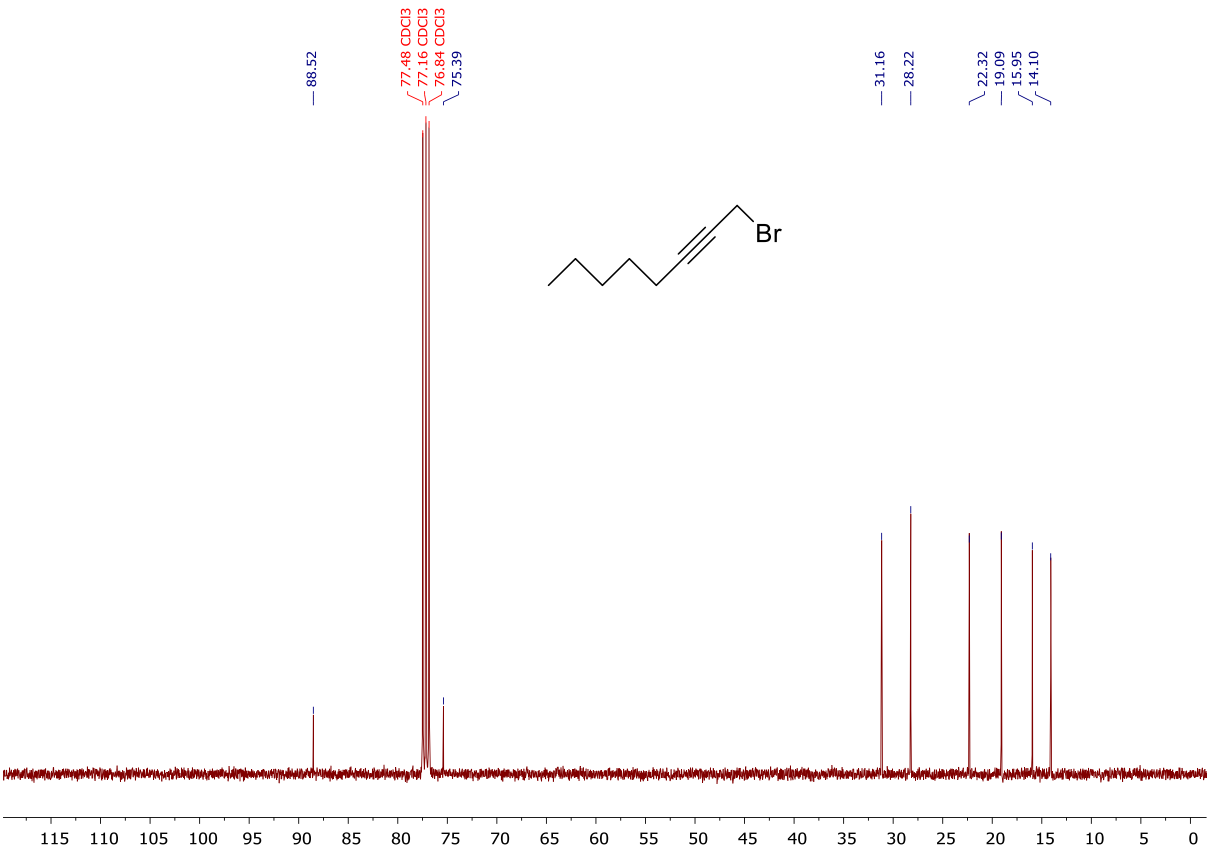

^{1}H-^{1}H COSY spectrum of compound **18**

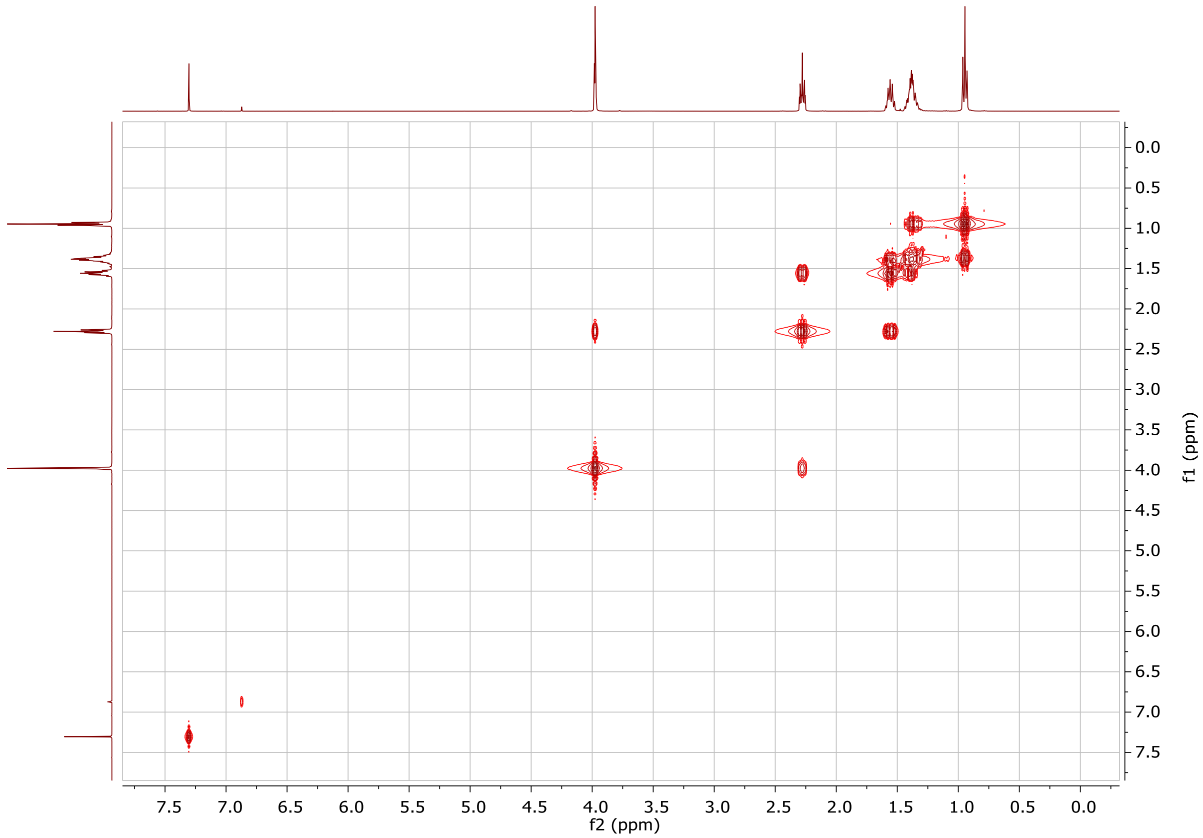

^{13}C-^{1}H HSQC spectrum of compound **18**

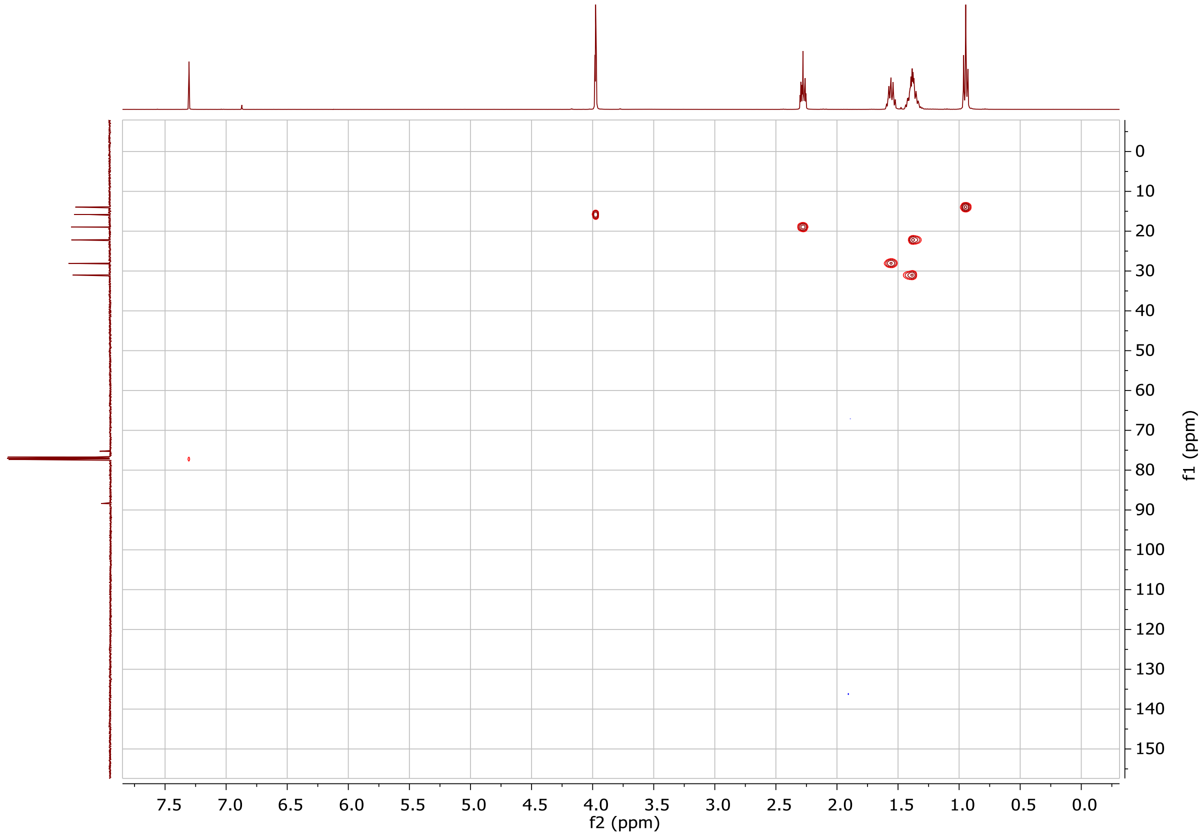

^{1}H NMR (400 MHz, $CDCl_3$) of compound **19**

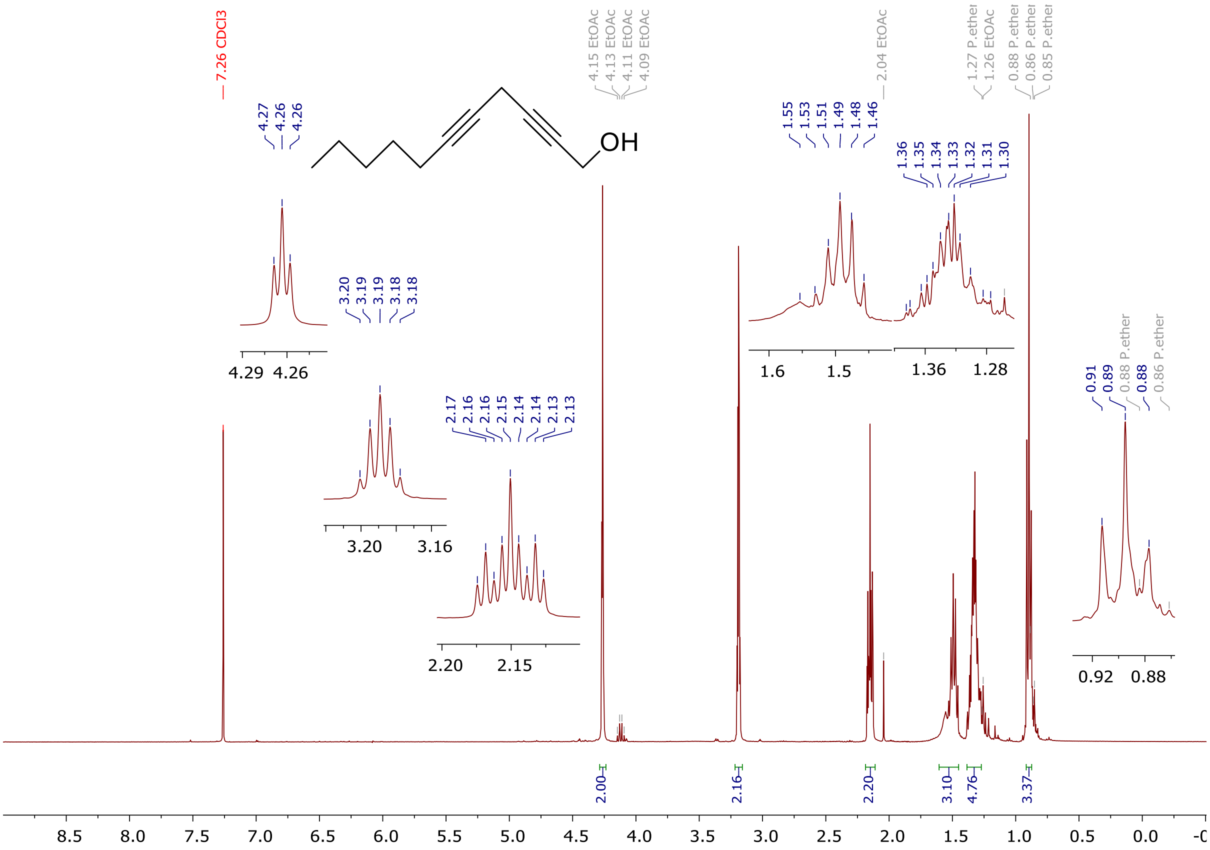

$^{13}C\{H\}$ NMR (101 MHz, $CDCl_3$) of compound **19**

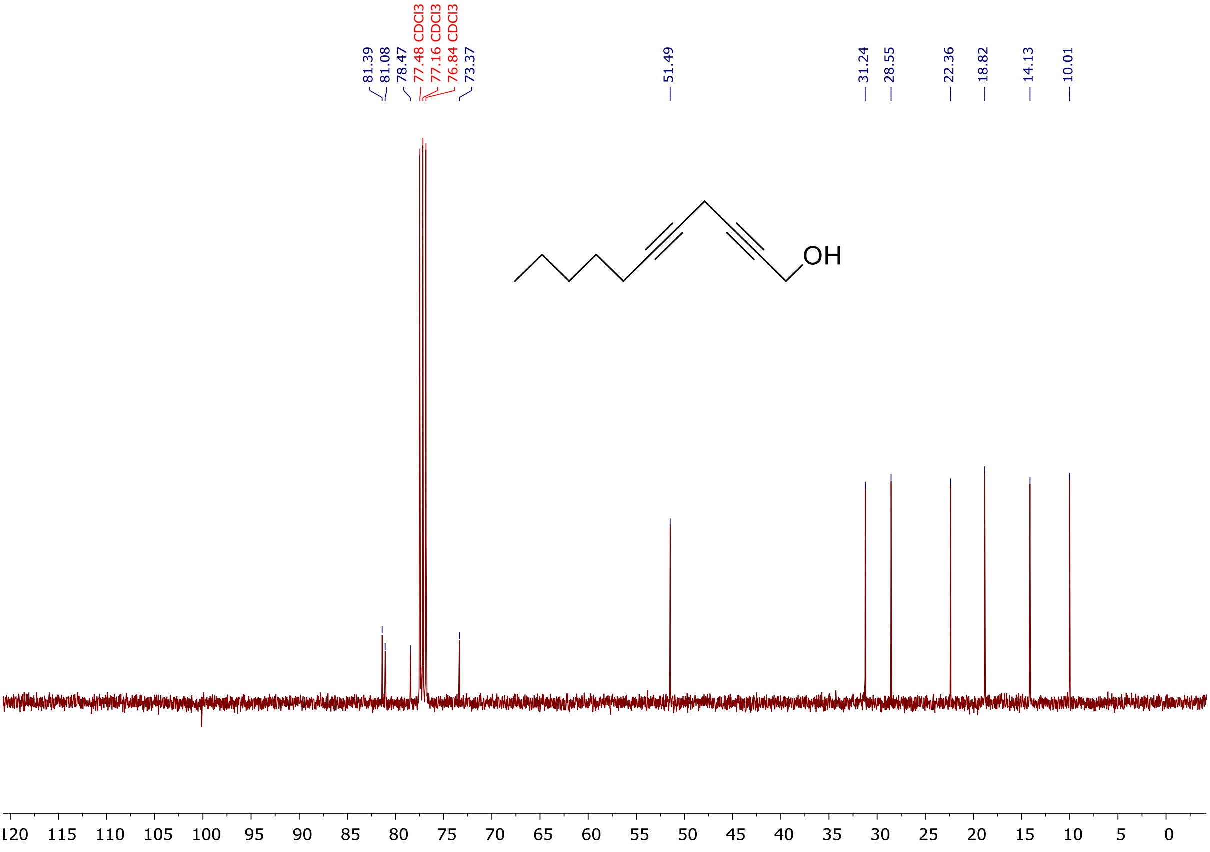

^{1}H-^{1}H COSY spectrum of compound **19**

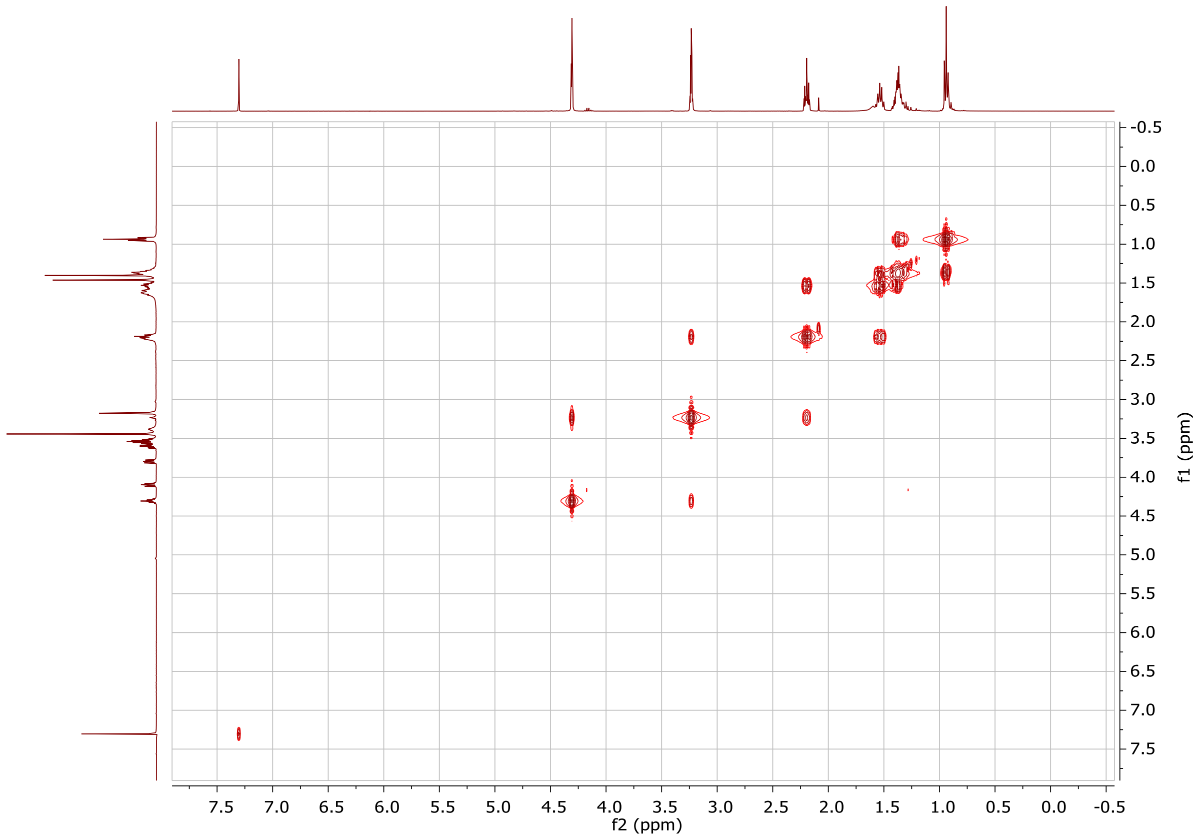

^{13}C-^{1}H HSQC spectrum of compound **19**

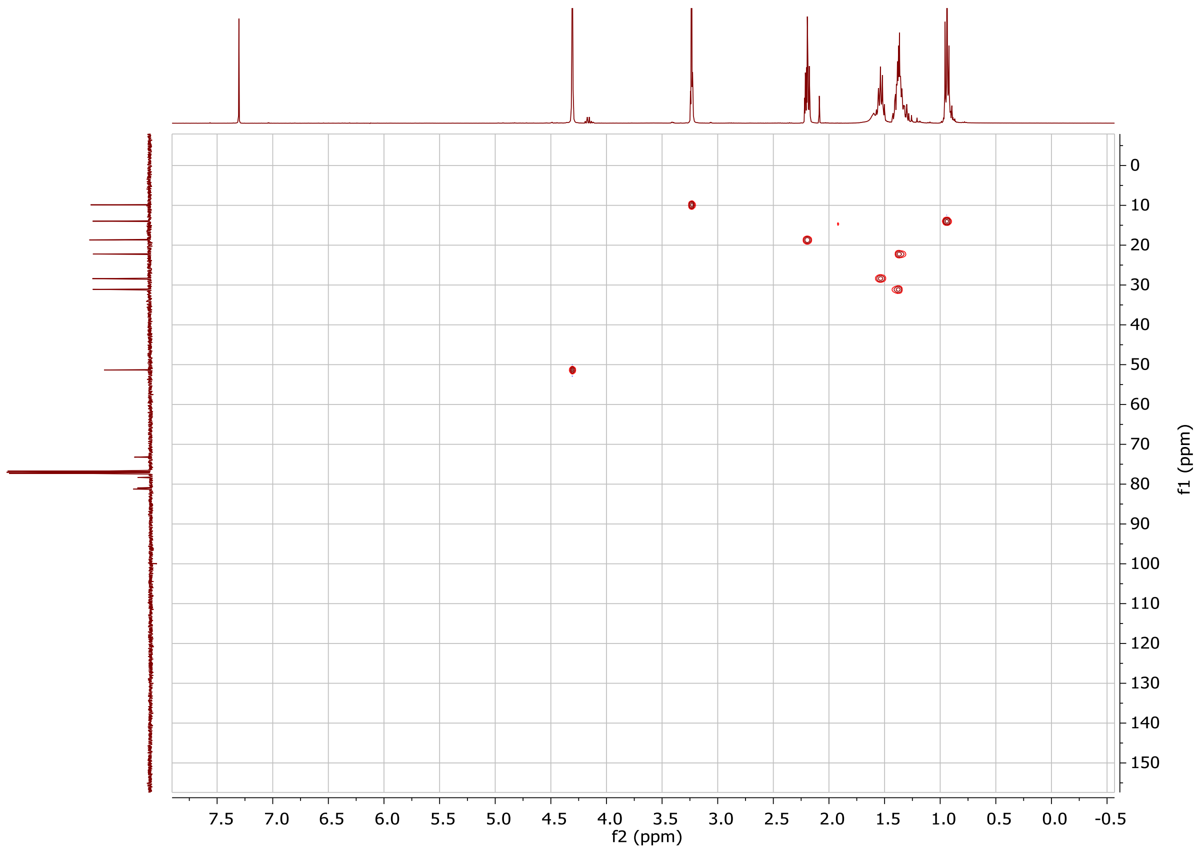

^{1}H NMR (400 MHz, $CDCl_3$) of compound **20**

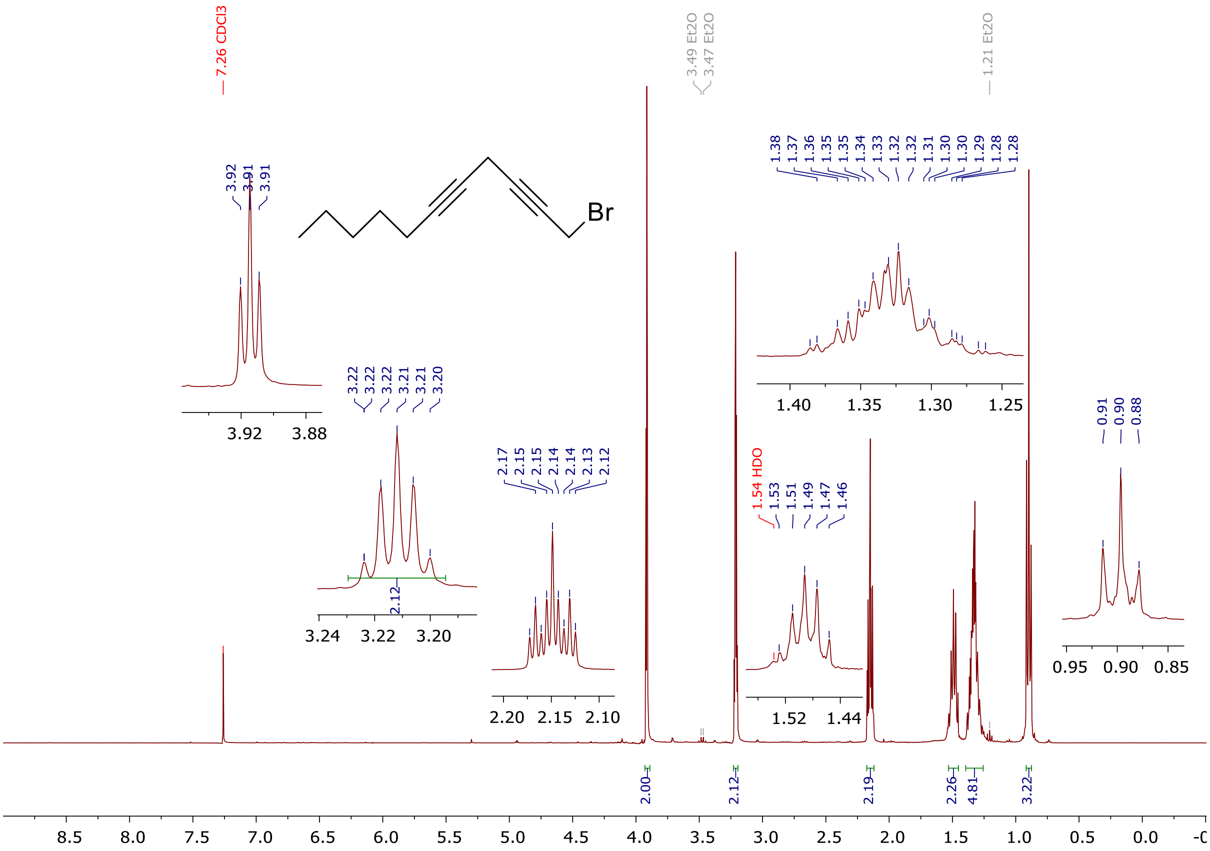

^{13}C{H} NMR (101 MHz, $CDCl_3$) of compound **20**

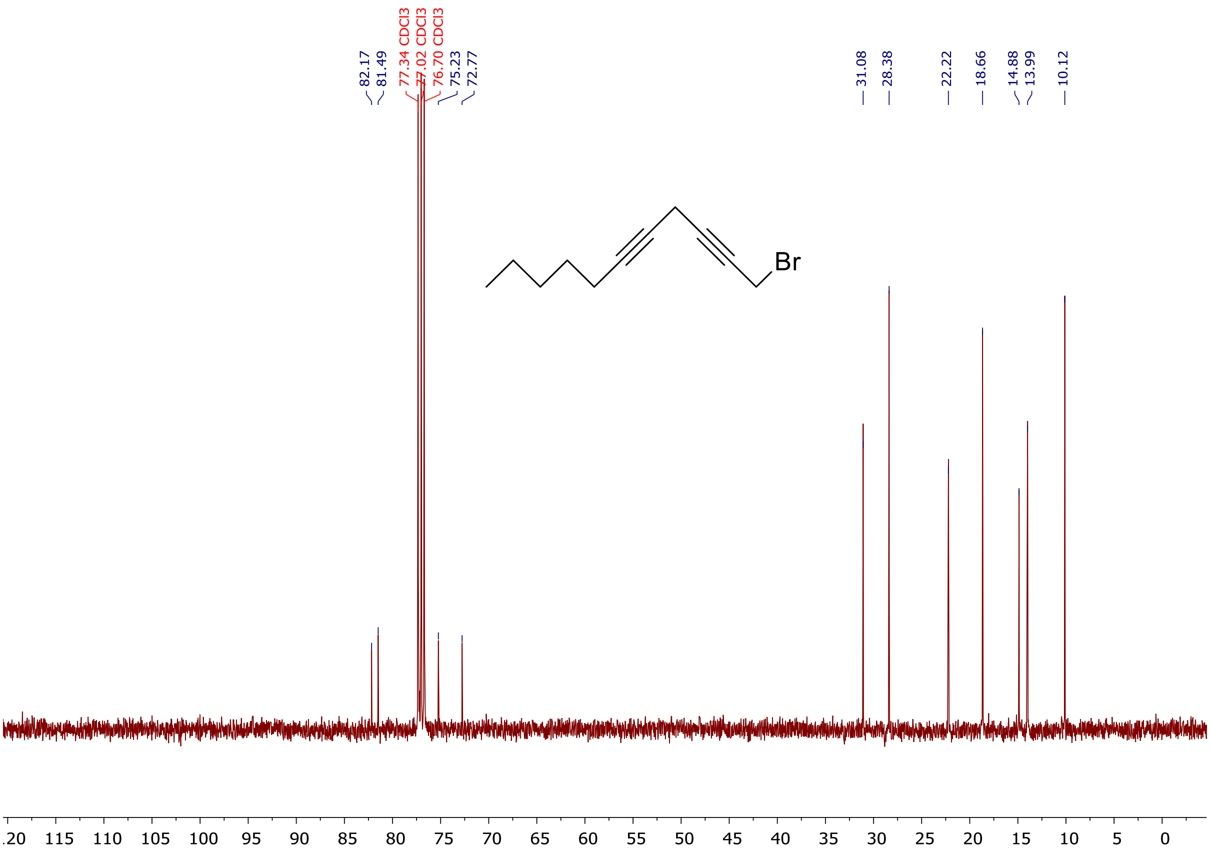

^{1}H-^{1}H COSY spectrum of compound **20**

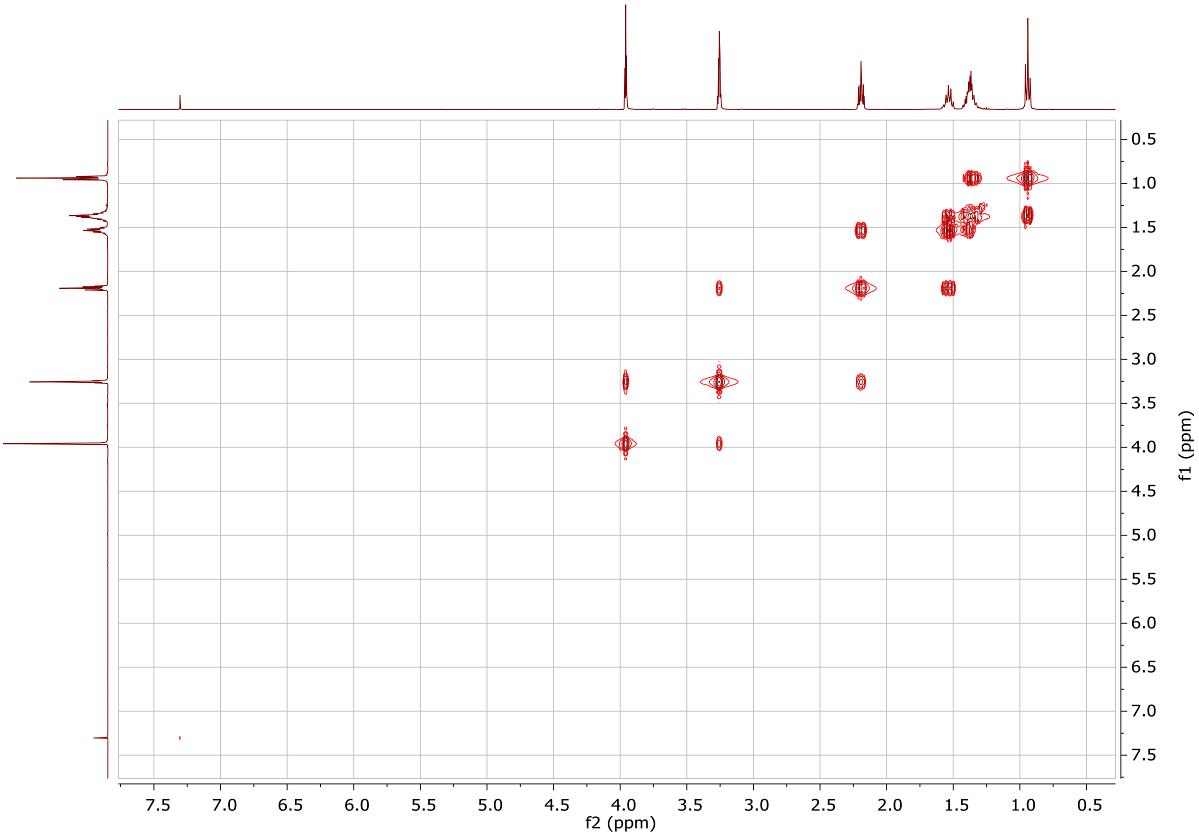

^{13}C-^{1}H HSQC spectrum of compound **20**

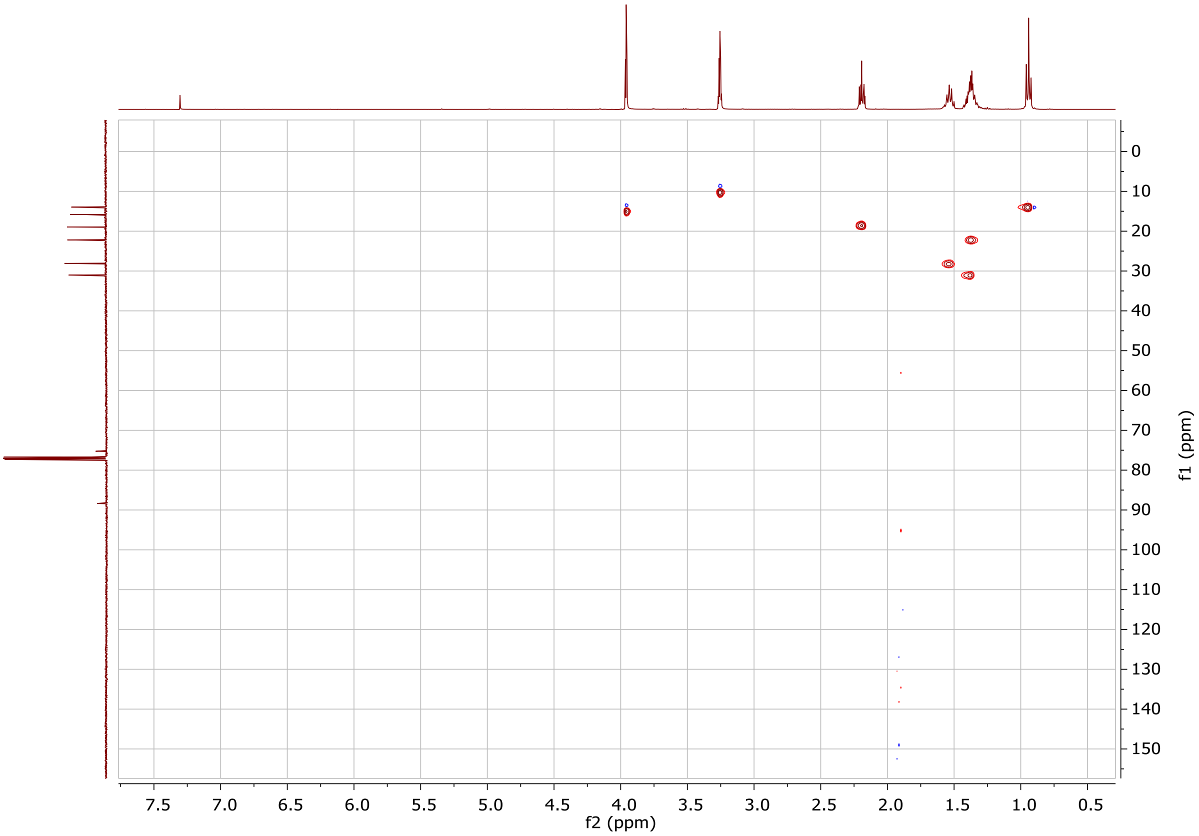

^{1}H NMR (400 MHz, $CDCl_3$) of compound **21**

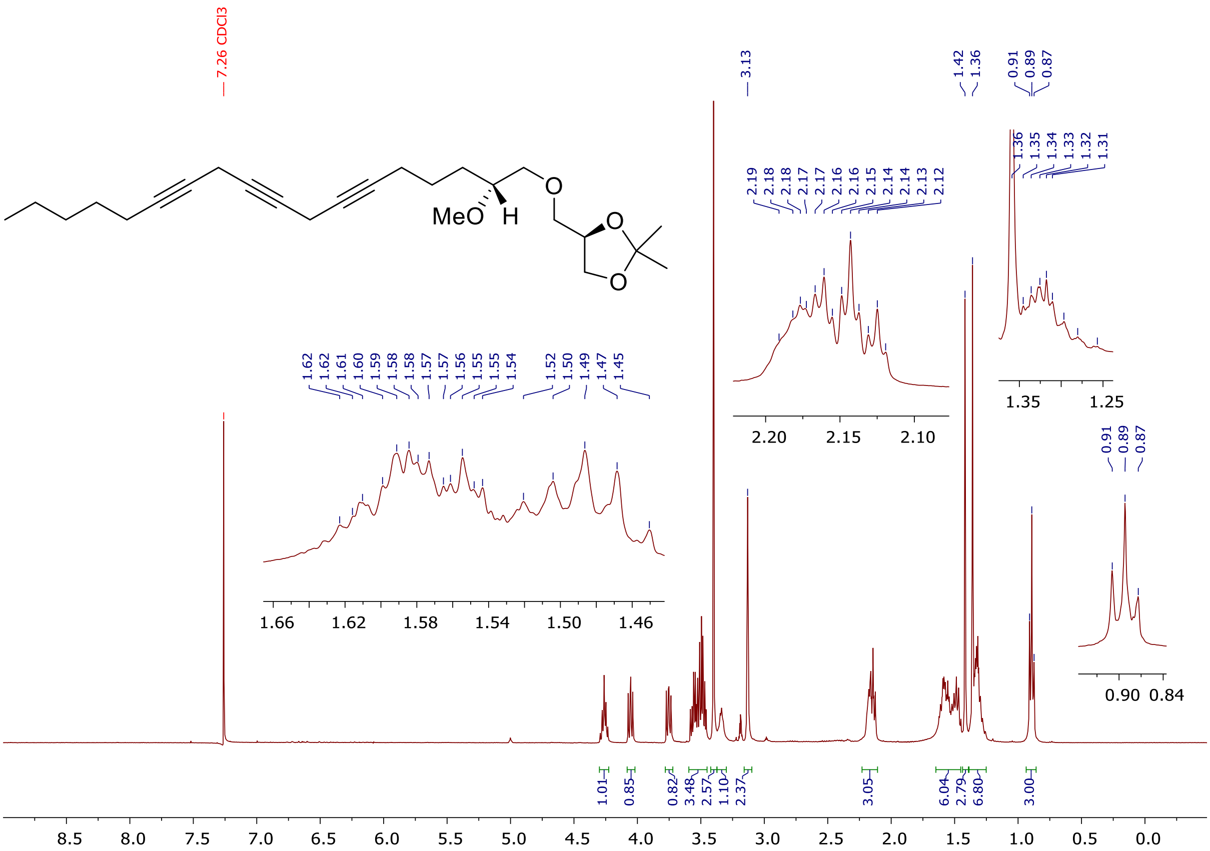

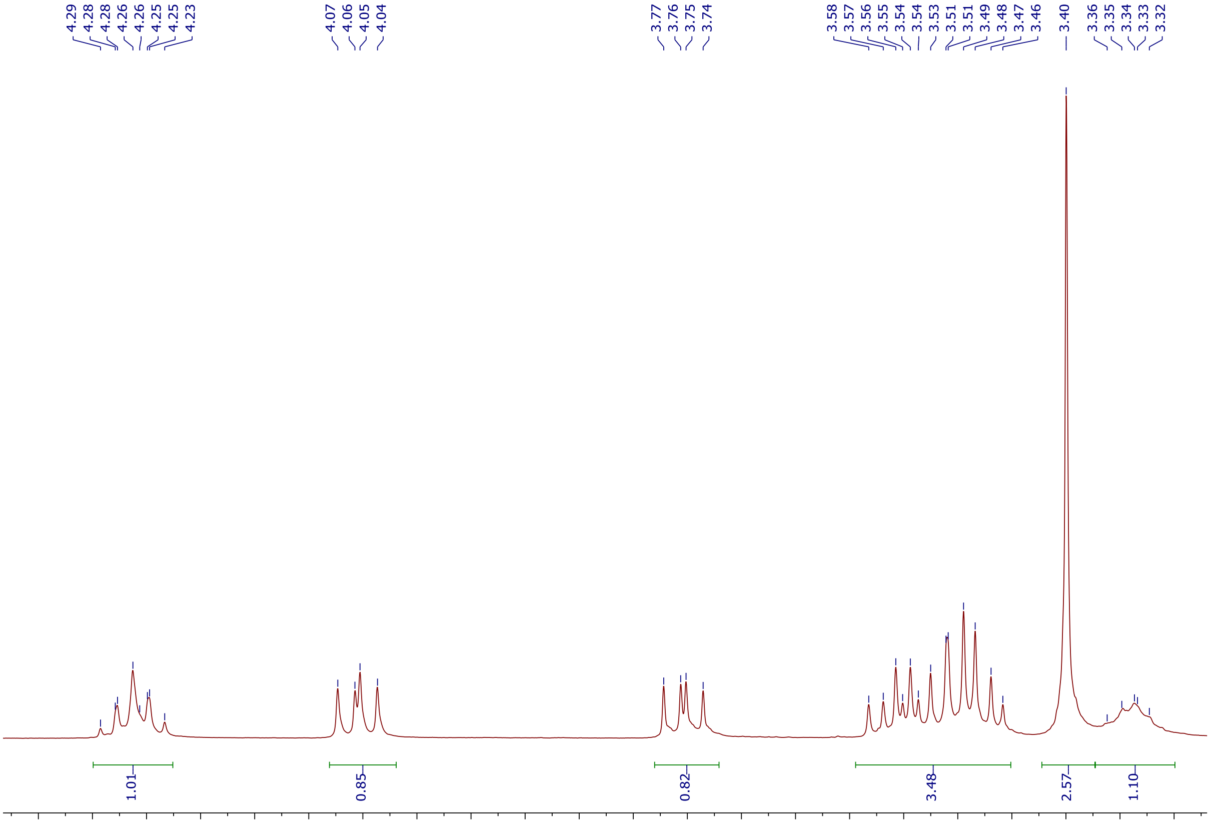

$^{13}C\{H\}$ NMR (101 MHz, $CDCl_3$) of compound **21**

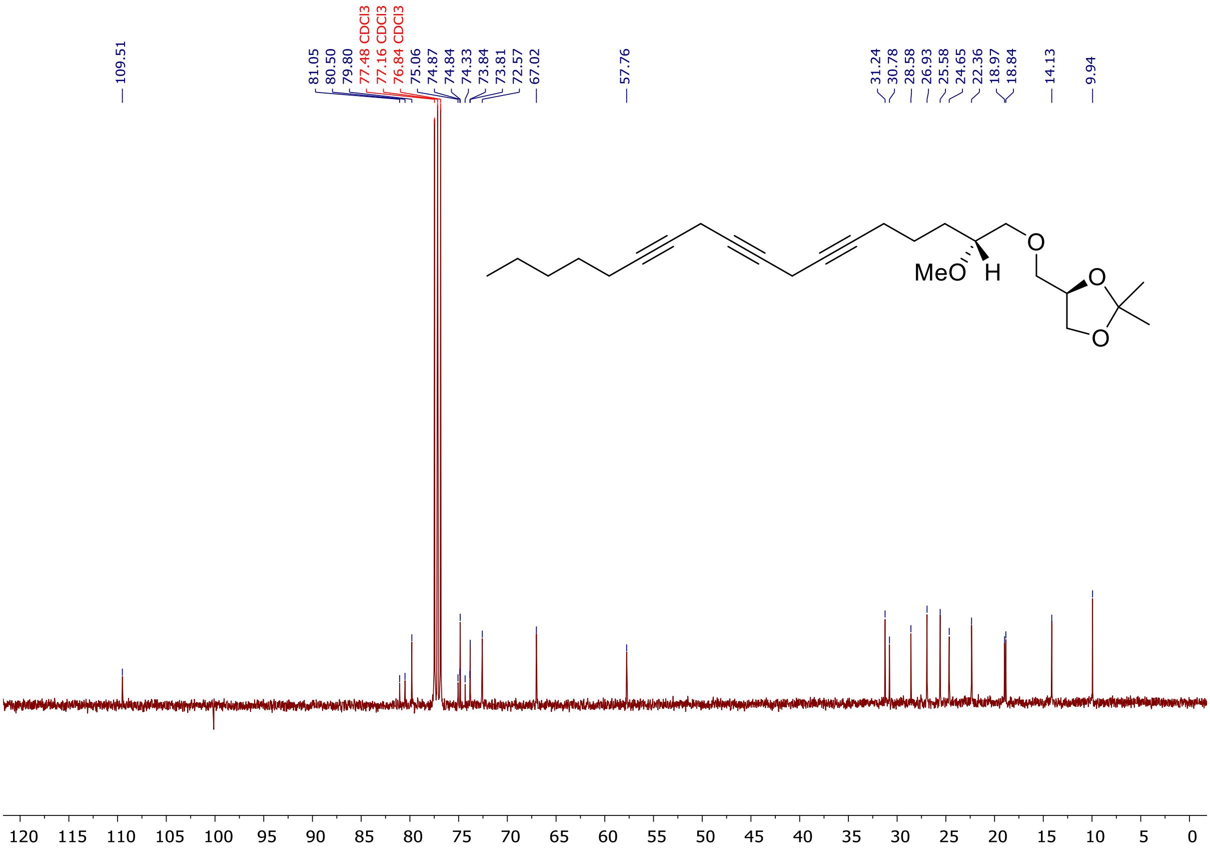

^{1}H-^{1}H COSY spectrum of compound **21**

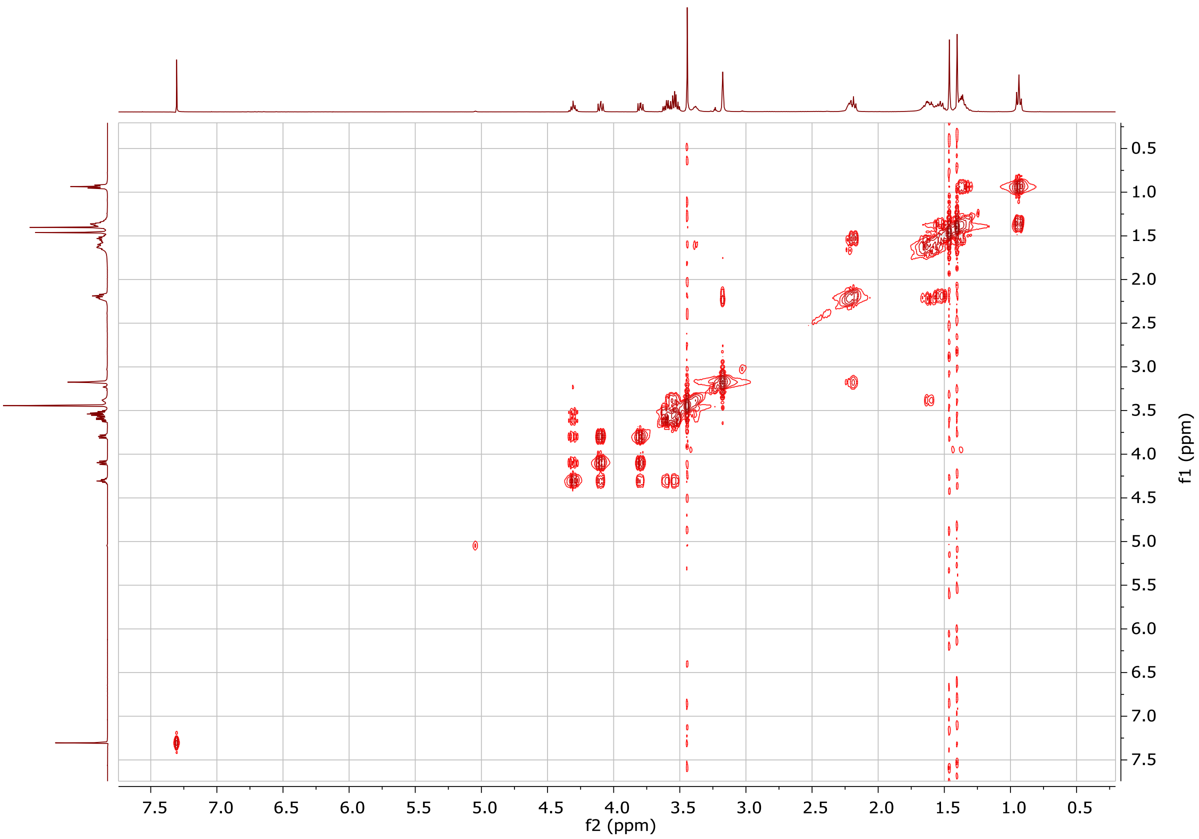

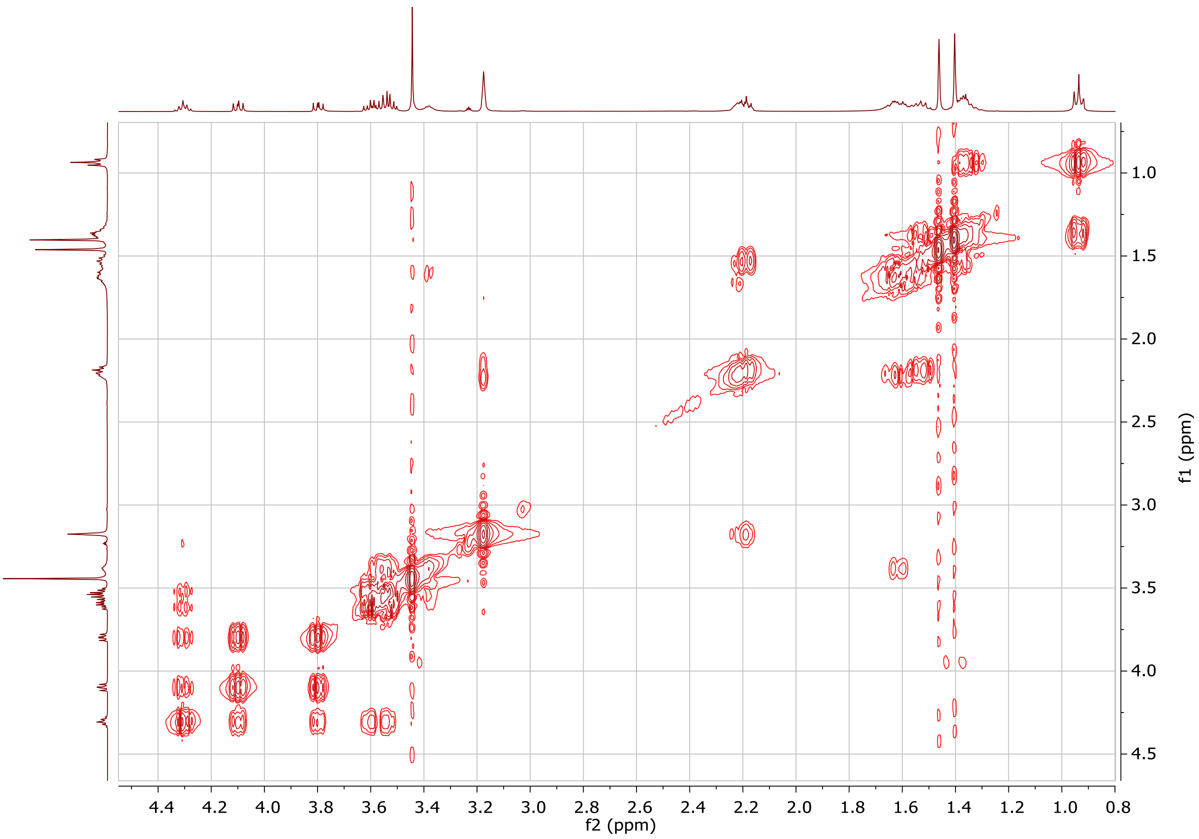

^{13}C-^{1}H HSQC spectrum of compound **21**

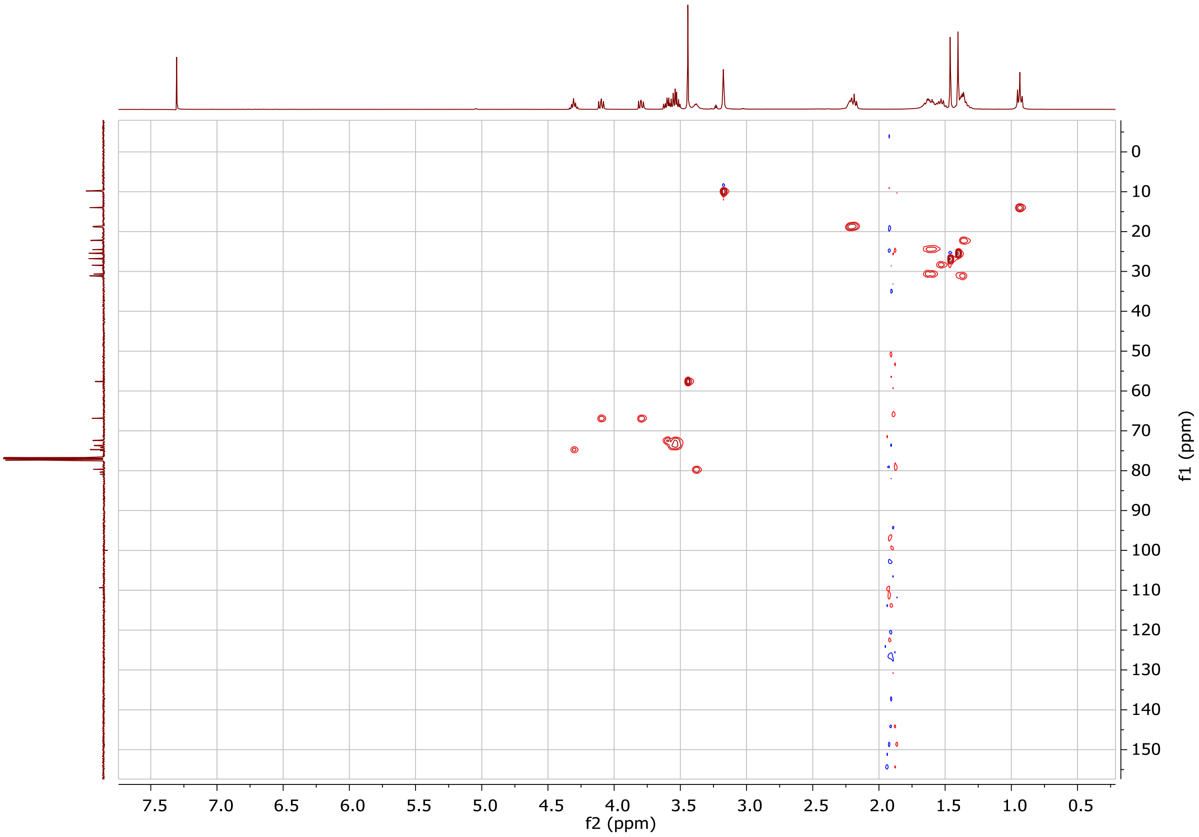

^{1}H NMR (400 MHz, $CDCl_3$) of compound **22**

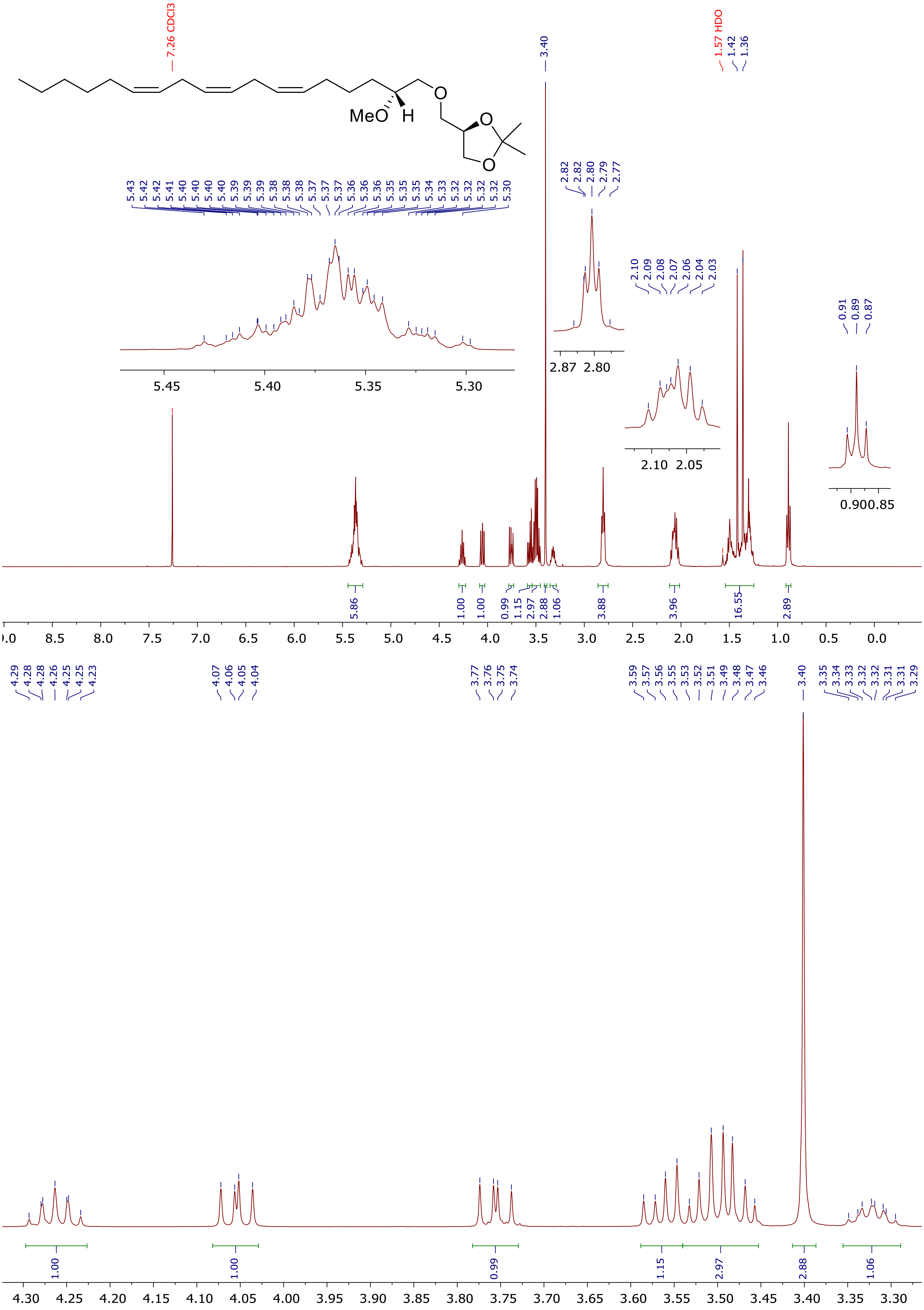

$^{13}C\{H\}$ NMR (101 MHz, $CDCl_3$) of compound **22**

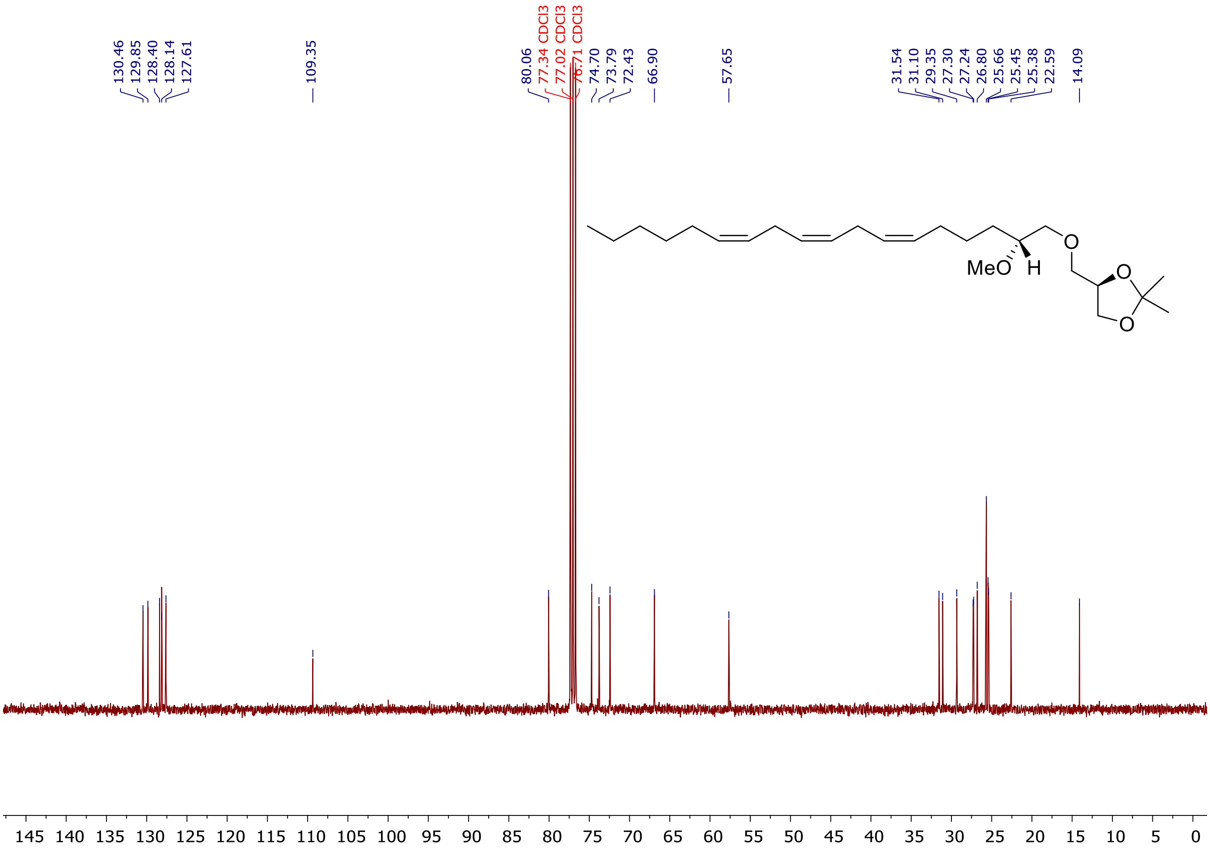

^{1}H-^{1}H COSY spectrum of compound **22**

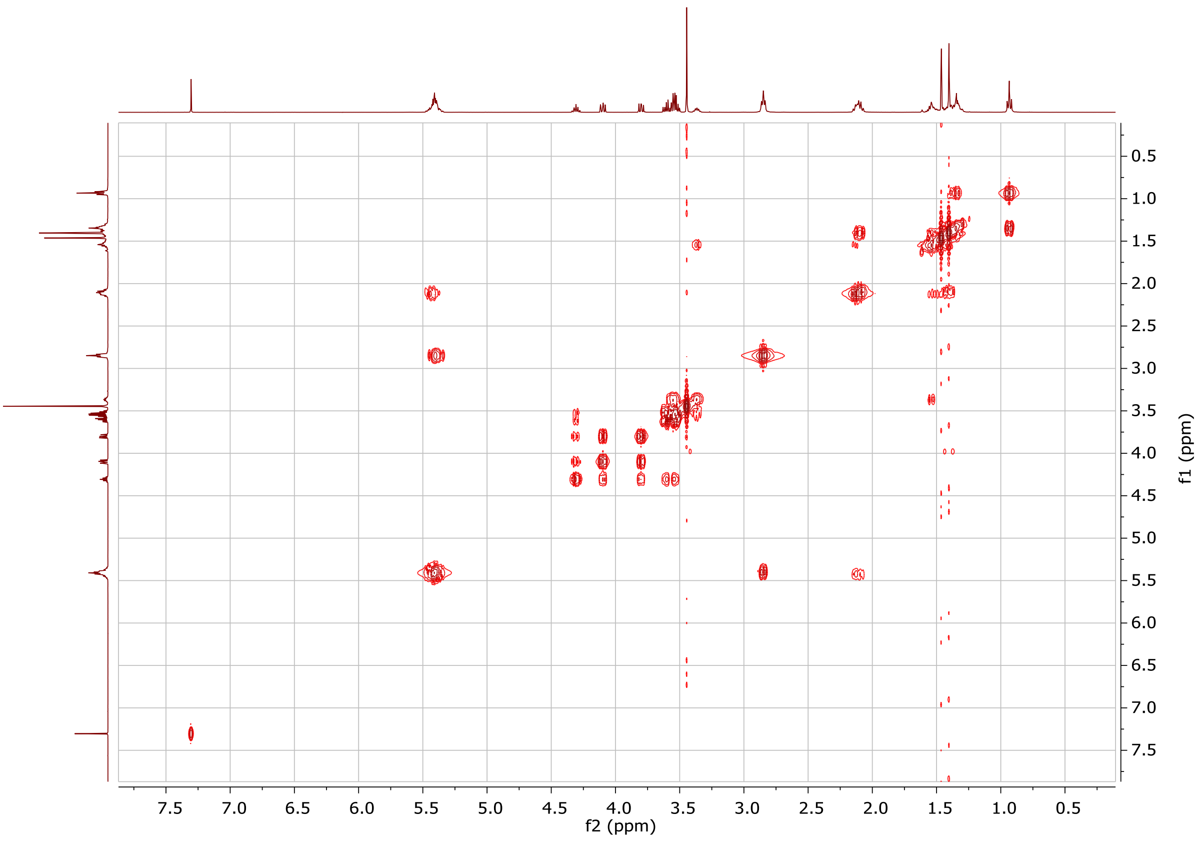

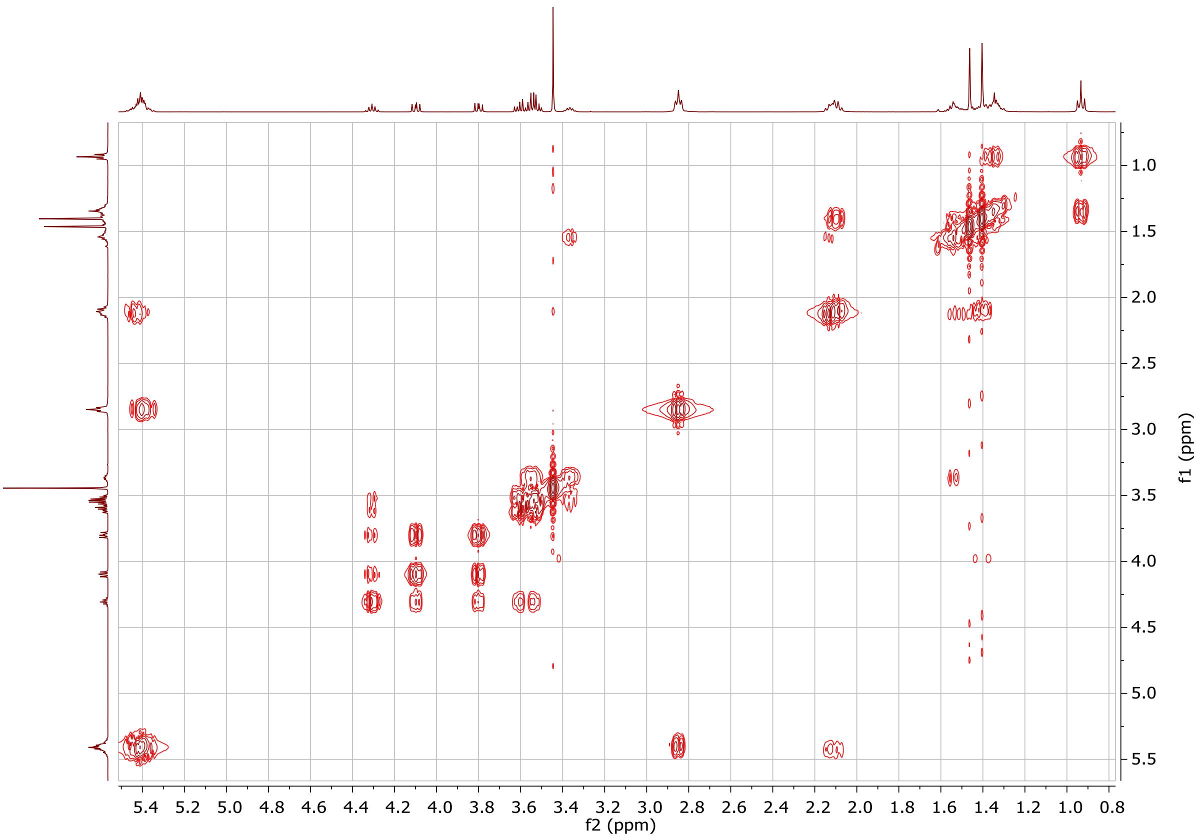

^{13}C-^{1}H HSQC spectrum of compound **22**

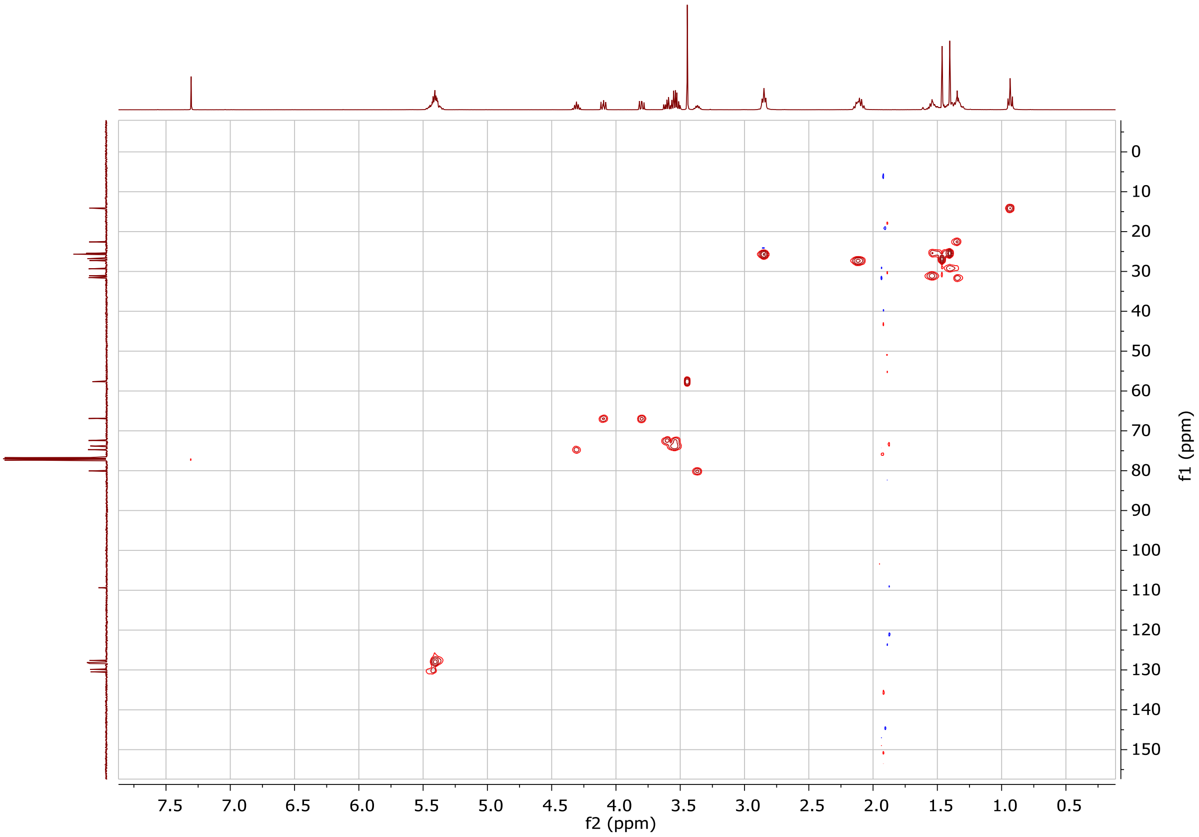

^{1}H NMR (400 MHz, $CDCl_3$) of MEL **6**

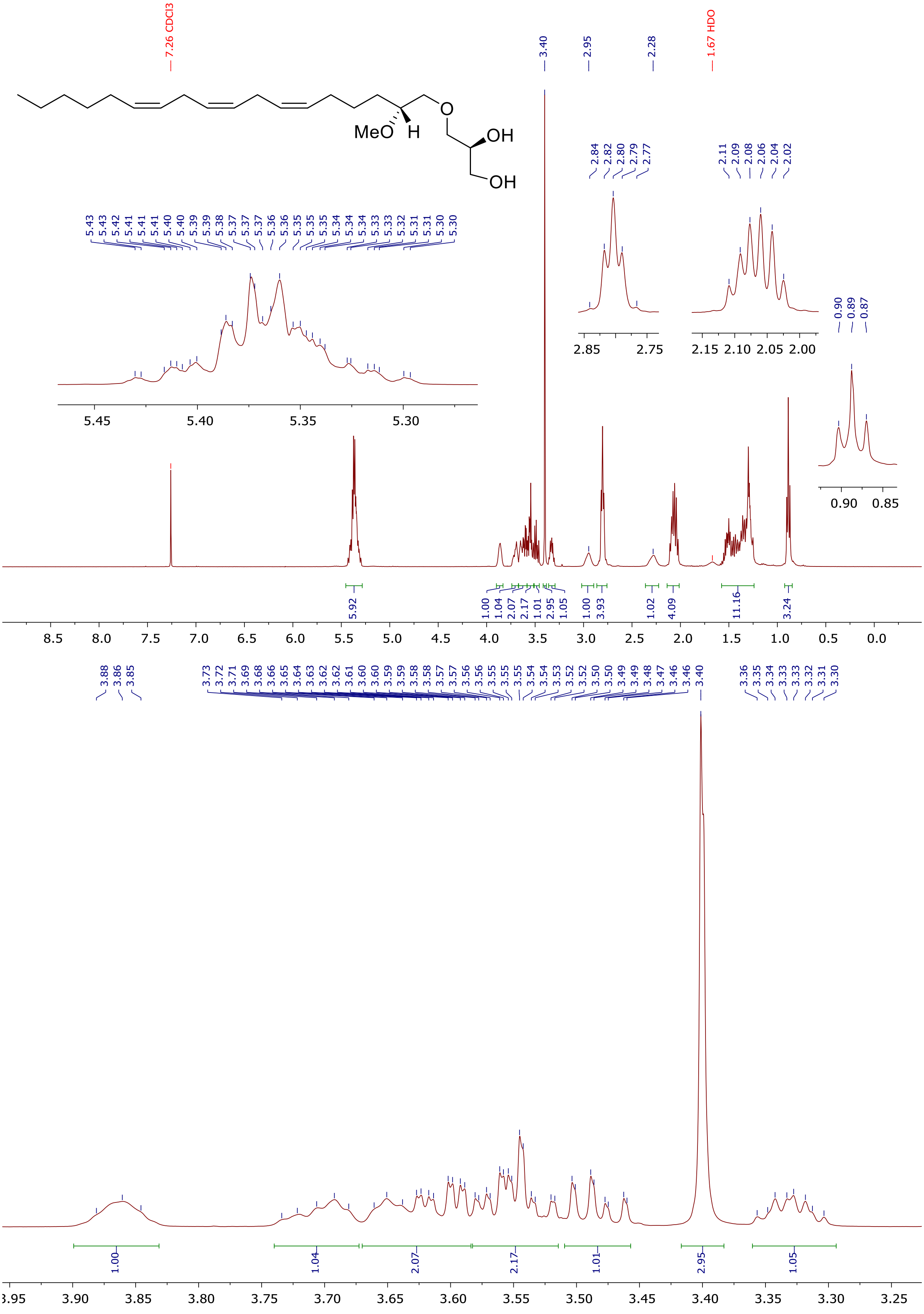

$^{13}C\{H\}$ NMR (101 MHz, $CDCl_3$) of MEL **6**

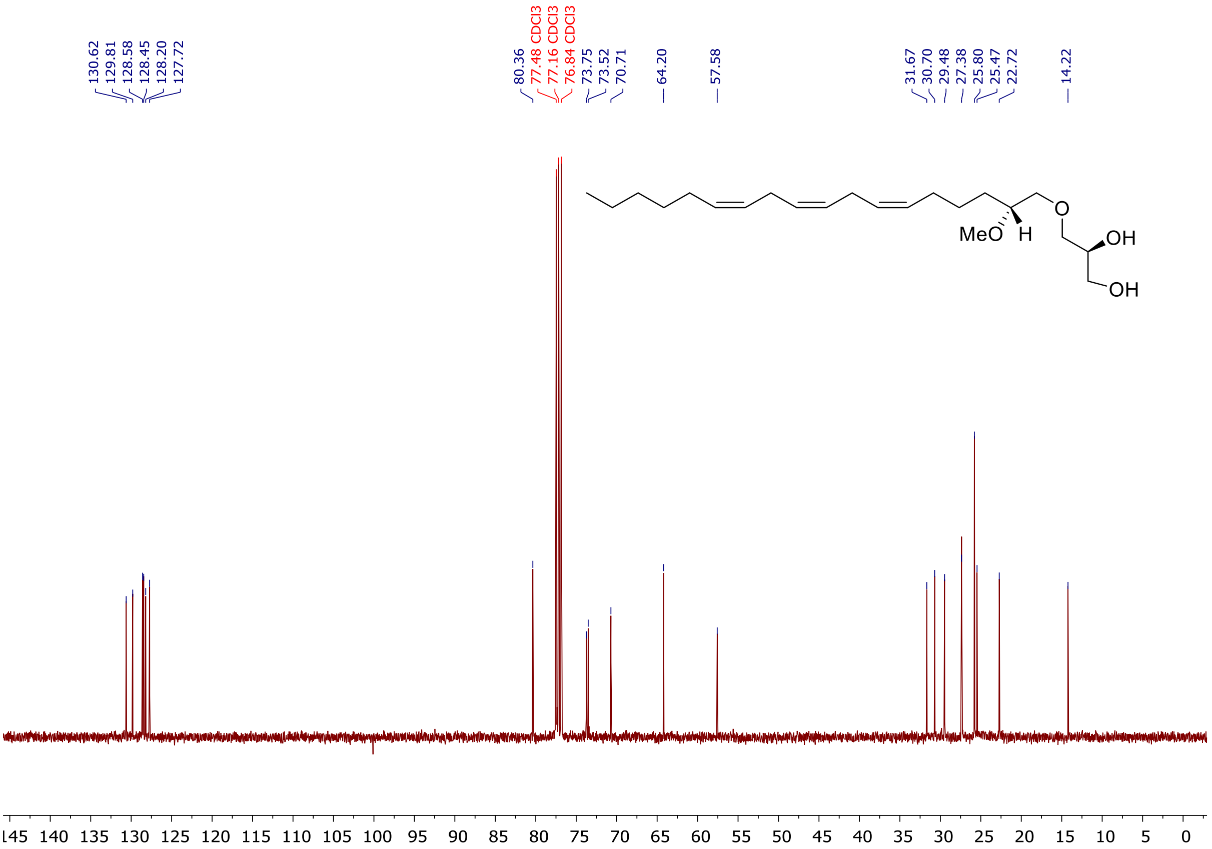

^{1}H-^{1}H COSY spectrum of MEL **6**

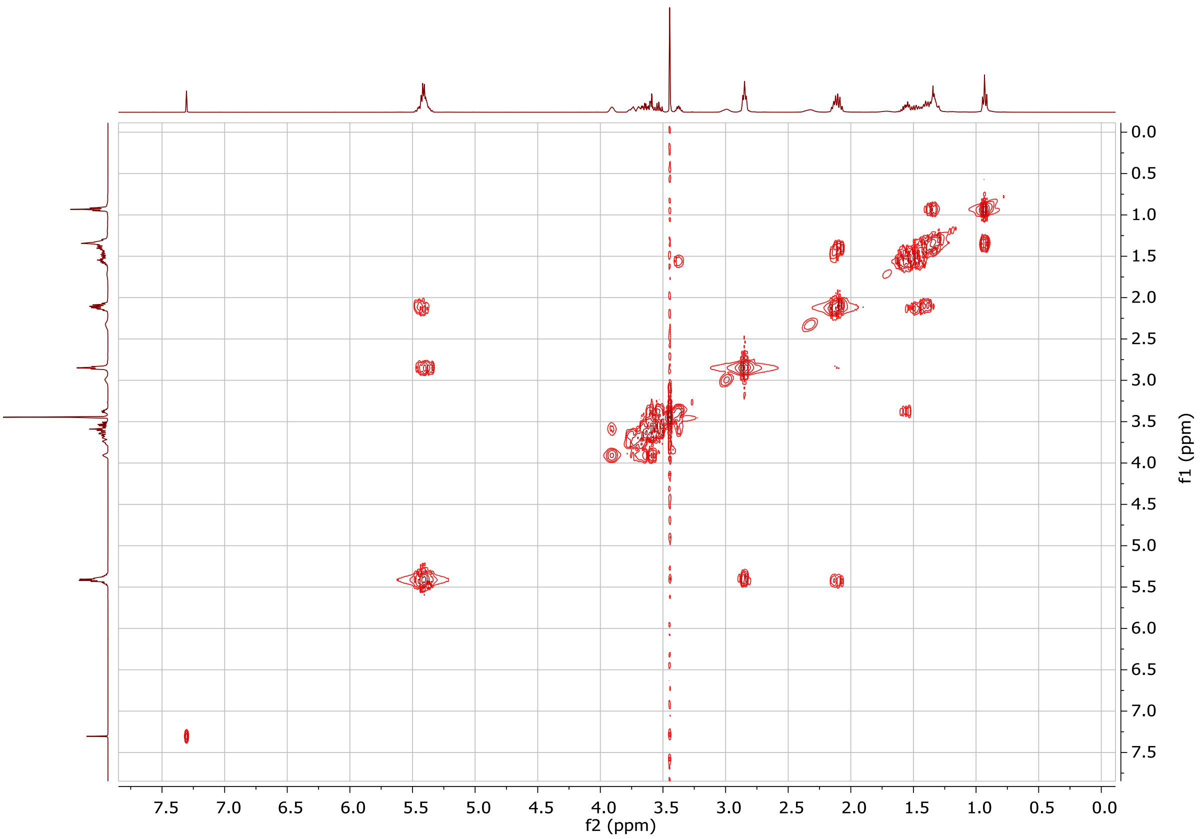

^{13}C-^{1}H HSQC spectrum of MEL **6**

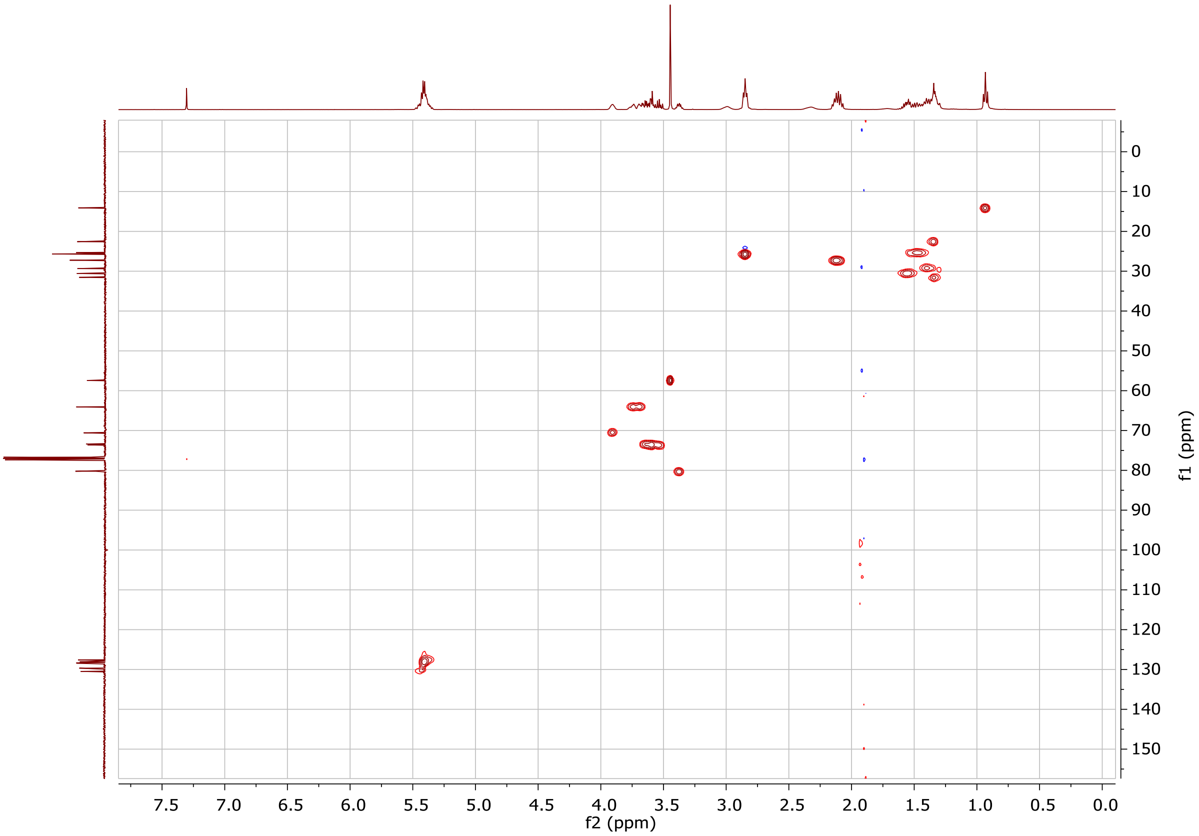

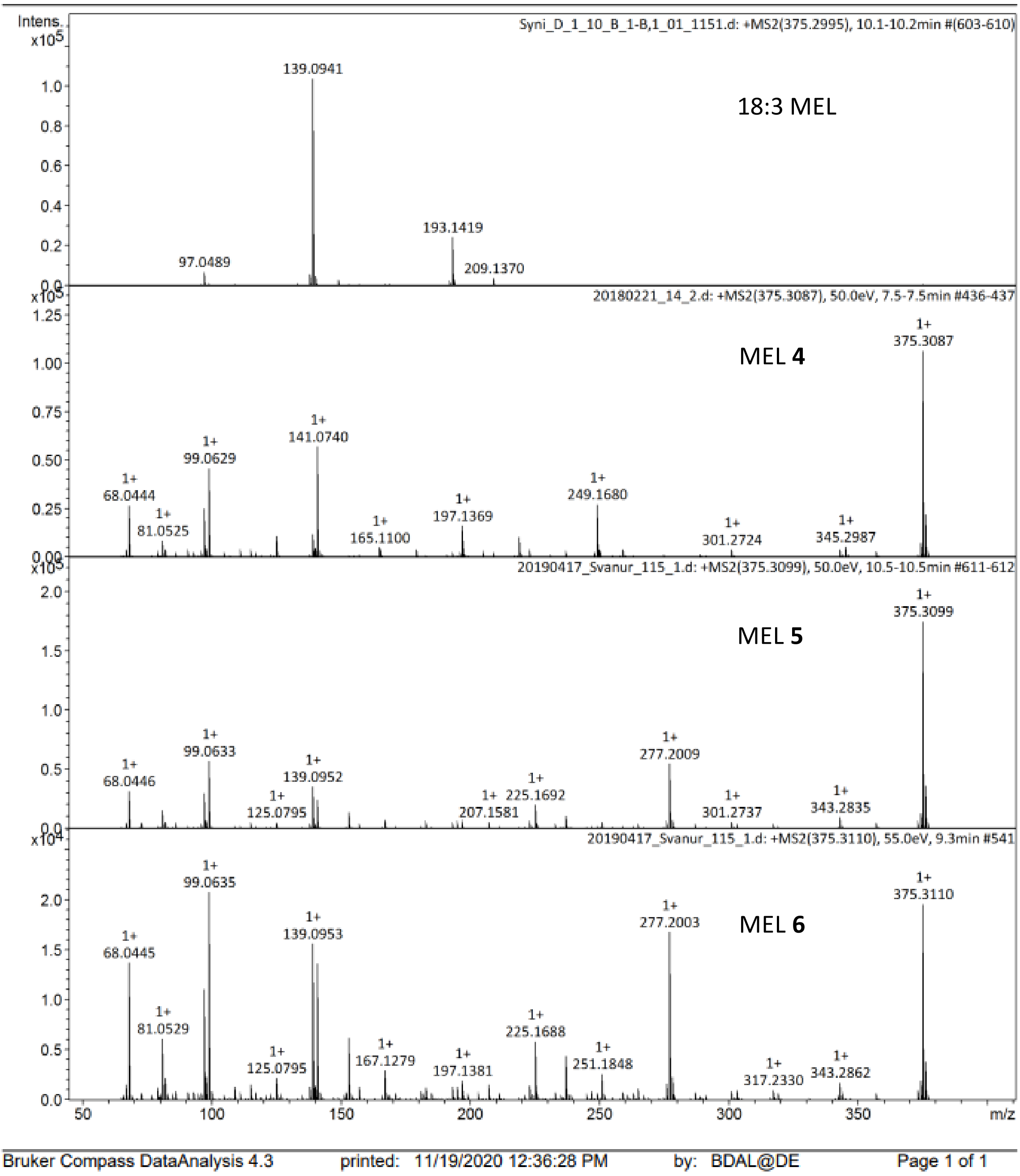

Figure S*1*. Comparison of the $[M+Li]^+$ MS/MS fragmentation spectra of the synthesized MELs **4**, **5**, **6** and the 18:3 MEL found in the shark and dogfish liver oil sample (top).
